# Lipid analogs reveal features critical for hemolysis and diminish granadaene mediated Group B Streptococcus infection

**DOI:** 10.1038/s41467-020-15282-0

**Published:** 2020-03-20

**Authors:** Blair Armistead, Pilar Herrero-Foncubierta, Michelle Coleman, Phoenicia Quach, Christopher Whidbey, Jose Justicia, Ruben Tapia, Raquel Casares, Alba Millán, Ali Haidour, Javier Rodriguez Granger, Jay Vornhagen, Verónica Santana-Ufret, Sean Merillat, Kristina Adams Waldorf, Juan Manuel Cuerva, Lakshmi Rajagopal

**Affiliations:** 1 Department of Global Health, University of Washington, Seattle, WA USA; 2 Center for Global Infectious Disease Research, Seattle Children’s Research Institute, Seattle, WA USA; 3 Department of Organic Chemistry, University of Granada, Granada, Spain; 4 Department of Microbiology, Virgen de las Nieves Hospital, Granada, Spain; 5 Department of Obstetrics and Gynecology, University of Washington School of Medicine, Seattle, WA USA; 6 Center for Innate Immunity and Immune Disease, University of Washington, Seattle, WA USA; 7 Sahlgrenska Academy, University of Gothenburg, Gothenburg, Sweden; 8 Department of Pediatrics, University of Washington School of Medicine, Seattle, WA USA; 9 Present Address: Seattle University, Seattle, WA USA; 10 Present Address: University of Michigan, Ann Arbor, MI USA

**Keywords:** Chemical tools, Lipids, Synthetic biology, Immunology, Bacterial infection

## Abstract

Although certain microbial lipids are toxins, the structural features important for cytotoxicity remain unknown. Increased functional understanding is essential for developing therapeutics against toxic microbial lipids. Group B Streptococci (GBS) are bacteria associated with preterm births, stillbirths, and severe infections in neonates and adults. GBS produce a pigmented, cytotoxic lipid, known as granadaene. Despite its importance to all manifestations of GBS disease, studies towards understanding granadaene’s toxic activity are hindered by its instability and insolubility in purified form. Here, we report the synthesis and screening of lipid derivatives inspired by granadaene, which reveal features central to toxin function, namely the polyene chain length. Furthermore, we show that vaccination with a non-toxic synthetic analog confers the production of antibodies that inhibit granadaene-mediated hemolysis ex vivo and diminish GBS infection in vivo. This work provides unique structural and functional insight into granadaene and a strategy to mitigate GBS infection, which will be relevant to other toxic lipids encoded by human pathogens.

## Abstract (web summary)

Granadaene, produced by Group B *Streptococcus* (GBS), is a long polyene lipid involved in cellular toxicity and hemolytic activity. Here, the authors synthesize and characterize granadaene-like compounds and show that a non-toxic analog diminishes GBS infection in mice when incorporated into a vaccine formulation.

## Introduction

Microbial lipids with hemolytic or cytotoxic activity have been reported from several human bacterial pathogens, including mycolactone from *Mycobacterium ulcerans*^1,2^, rhamnolipids from *Pseudomonas aeruginosa*^3,4^, commendamide from *Bacteroides* spp.^5^, and the pigmented ornithine rhamnopolyene (granadaene) from Group B *Streptococcus* (GBS)^6,7^. However, structure–activity studies demonstrating the features important for cytotoxicity are lacking, hindering the development of therapies or vaccines targeting these compounds. GBS is a β-hemolytic Gram-positive bacterium frequently associated with preterm birth and severe neonatal infections^8–10^. Although clinical trials involving maternal vaccination of a trivalent capsular polysaccharide-based CRM197 conjugate were shown to provide serotype-specific antibody^11^ and a six-valent vaccine is being explored^12^, no Food and Drug Administration-approved vaccine exists to date for GBS prevention. In recent years, the incidence of invasive GBS disease, including bloodstream, skin, soft tissue, and joint infections, has increased in non-pregnant adults, particularly among the elderly and persons with comorbidities^13–15^. These trends, along with concerns regarding the emergence of antibiotic-resistant GBS strains, have increased the urgency for novel preventive and curative treatments^16,17^.

Granadaene produced by GBS confers pigmentation^18^ and hemolytic activity^6,7^ and is a major contributor to all manifestations of GBS disease, making it an attractive therapeutic target. Hemolytic and hyper-hemolytic GBS strains have been isolated from patients with GBS infections, including women in preterm labor and adults with invasive infections^6,19–22^. Furthermore, non-hemolytic GBS strains are typically virulence-attenuated, and hyper-hemolytic GBS strains exhibit increased virulence in various models of infection, including sepsis, meningitis, and preterm birth^6,20,23–25^. In addition, we showed that purified granadaene isolated from GBS is hemolytic and cytotoxic to innate immune cells, including mast cells, macrophages, and neutrophils^6,7,24,26^. Despite these advances, the structural features responsible for hemolysis and cytotoxicity are unknown.

A major barrier for elucidating the functions of GBS ornithine rhamnopolyene is its limited solubility and instability in its purified form, which is typical for compounds containing long polyene chains^27^. Here, we use chemical synthesis to overcome these challenges associated with studying polyenic compounds. Through the synthesis of lipid derivatives featuring key structural components of granadaene, we demonstrate how the polyene chain length and polar head groups influence hemolytic activity. Further, we identify a non-toxic analog, which, unlike granadaene, is tolerated by cells of the adaptive immune system and diminished GBS infection when incorporated into a vaccine formulation. Together, these data advance our chemical, molecular, and biological understanding of a key GBS virulence factor whose mechanism has eluded us thus far. Moreover, our findings provide proof-of-concept for a promising therapeutic strategy to mitigate the effects of this lipid toxin during infection.

## Results

### Polyene chain length is critical for hemolysis

**Fig. 1:**
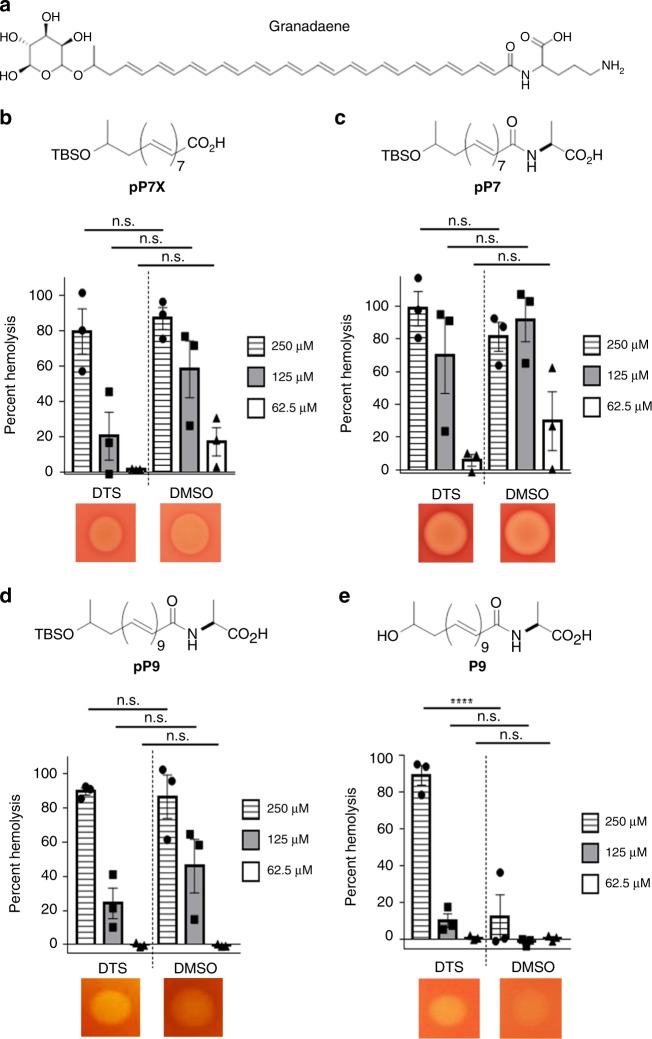
The polyene chain length is important for hemolytic activity. **a** Granadaene contains a terminal rhamnose, a polyene chain consisting of 12 double bonds, and a terminal ornithine. **b**–**e** Synthetic analogs of granadaene (structures shown) were resuspended in DTS (DMSO + trifluoroacetic acid (TFA, 0.1%) + starch (20%)) or DMSO. The analogs were co-incubated with human red blood cells at 250, 125, and 62.5 µM for 1 h at 37 °C, and hemoglobin release in cell supernatants was quantified to determine percent hemolysis relative to Triton X-100 (0.1%)-treated positive controls and PBS-treated negative controls. Mean and standard error from three independent experiments are shown. Differences in hemolysis between DTS resuspension and DMSO resuspension for a given concentration of each compound were analyzed using a two-way ANOVA with Tukey’s post test. **pP7X**: 250 μM, *p* = 0.9959, 125 μM, *p* = 0.2079, 62.5 μM, *p* = 0.9007; **pP7**: 250 μM, *p* = 0.9524, 125 μM, *p* = 0.8823, 62.5 μM, *p* = 0.8375; **pP9**: 250 μM, *p* = 0.9999, 125 μM, *p* = 0.5592, 62.5 μM, *p* > 0.9999; **P9**: 250 μM, *p* < 0.0001, 125 μM, *p* = 0.6957, 62.5 μM, *p* > 0.9999. Additionally, 5 µL of each synthetic analog at 0.02 M concentration was spotted onto red blood agar plates and allowed to incubate at 37 °C overnight. The hemolytic effect of each compound on red blood agar is shown. ****, *p* < 0.0001. n.s., Not significant.

To understand components necessary for granadaene-mediated hemolysis and cytolysis, we identified target compounds for synthesis that contained chemical features analogous to those in granadaene (Fig. 1a) and were sufficiently soluble and stable in vitro. We focused on three relevant moieties: the polyene chain, the terminal amino acid, and the rhamnose. The chemical structure of each target compound that was synthesized is listed in Table 1. The synthetic compounds were designed to contain a varying number of alkenes, ranging from one to nine, denoted in the compound names by “P” and then the number of alkenes in the polyene chain (e.g., **P9**). The rhamnose group found in granadaene (Fig. 1a) was replaced with hydrophobic *tert*-butyldimethylsilyl (TBS)-protecting group (denoted by “p”) in some target compounds, namely **pP7X**, **pP7**, **pP9**, and **pP1**, to ensure reasonable solubility of these derivatives during the synthetic sequence and subsequent biological tests. One compound, **R-P4**, was sufficiently stable, which enabled us to include a terminal rhamnose moiety without compromising solubility. In other compounds, such as **P7** and **P9**, a hydroxyl group was included to test the effect of a hydrophilic head group. Because the l-ornithine residue at the terminus of granadaene was previously observed to lactamize in several synthetic reaction conditions^28^, which could confound interpretation of the biological assays, it was replaced with a simpler l-alanine residue in all synthetic compounds except **pP7X**, which contained a carboxyl group instead; this compound was synthesized to test the effect of the terminal amino acid on hemolytic activity. Each of the target compounds was fully characterized (see Methods and Supplementary Materials) and analyzed by mass spectrometry and proton nuclear magnetic resonance (^1^H NMR) and carbon-13 NMR (^13^C NMR) (see Supplementary Materials).

**Table 1:** The chemical structures and estimated polyene chain lengths and calculated logarithm of its partition coefficient between *n*-octanol and water (cLogP) of synthetic analogs and granadaene.

Compound name	Structure	Length of polyene chain (Å)	cLogP
**pP1**		3.63	2.43
**R-P4**		10.8	0.52
**pP7X**		20.8	6.73
**pP7**		17.9	6.08
**P7**		17.7	2.74
**pP9**		22.6	7.29
**P9**		22.4	3.95
Granadaene		29.7	2.95

**Fig. 2:**
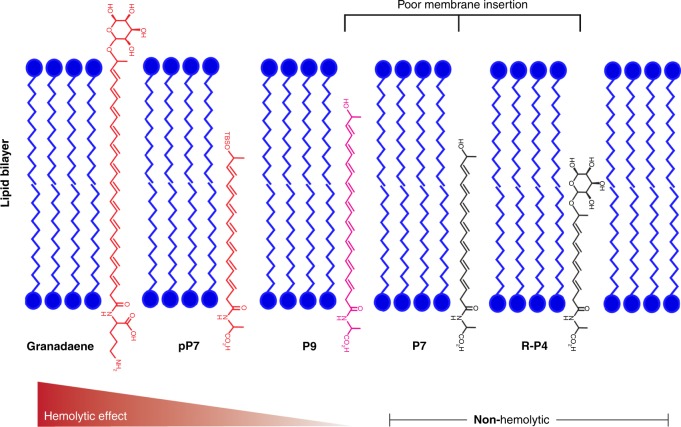
Proposed role of polyenes and polar head groups for granadaene-mediated hemolysis and cytolysis. The polyene chain of granadaene spans the length of the target cell’s lipid bilayer and the two polar head groups, rhamnose and ornithine, allow for stable insertion of the GBS ornithine rhamnopolyene into the cell membrane, leading to membrane disruption and hemolysis or cytolysis. Shorter polyenes such as those containing seven double bonds are hemolytic only when one polar end is replaced with a hydrophobic group (e.g., TBS), as in **pP7**, in which the molecule stably inserts in the membrane due to the compatibility between the hydrophobic lipid bilayer and the TBS group. However, if both polar ends are maintained in compounds with shorter polyenes, as in **P7** or **R-P4**, no hemolytic activity is observed because stable insertion into the cell membrane is not favored. Once the polyene chain reaches a sufficient length, as in **P9**, some hemolytic activity is observed even when both polar head groups are maintained. Together, this suggests that the length of the polyene chain and polar head groups found in granadaene are key to stable membrane disruption of host cells.

To examine if analogs had hemolytic activity, we first examined their ability to lyse red blood cells (RBCs) on red blood agar plates and then quantitatively examined them for lysis of RBCs using methods described^6^. While **pP1**, **R-P4**, and **P7** showed no hemolytic activity (Supplementary Fig. 1), hemolysis was observed in **pP7X**, **pP7**, **pP9**, and **P9** (Fig. 1b–e), which indicates that the length of the polyene chain facilitates hemolysis. In addition, these results suggest that the protecting group is important for the activity of compounds with shorter polyene chains. The proposed role of the polyene chain length and head groups in granadaene-mediated hemolysis is illustrated in Fig. 2. Except for **P9**, no statistically significant difference in hemolysis was observed between compounds resuspended in DTS (DMSO (dimethyl sulfoxide)  +  0.1% TFA (trifluoroacetic acid) + 20% starch) versus DMSO, indicating that starch, which is used to stabilize purified granadaene solutions^6^, may be important to stabilize longer polyene chains that are unprotected. Of note, no hemolysis was observed in RBCs treated with DTS or DMSO alone (Supplementary Fig. 1). Collectively, these findings emphasize the importance of the length of the polyene chain to GBS hemolysis, thus providing insight into the chemical features key to granadaene activity.

### Granadaene is cytotoxic to CD4^+^ T and B cells

In addition to lysis of RBCs, we have described the cytotoxic activity of granadaene to various innate immune cells, including mast cells, neutrophils, and macrophages^6,7,24,26^. To date, the effect of granadaene on adaptive immune cells has not been described, but is critical to determine for vaccine strategies. Furthermore, antibodies specific to this toxin have not been identified. As a few studies have reported that GBS can activate CD4^+^ T cells^29,30^, we explored the effect of granadaene on adaptive immune cells.

**Fig. 3:**
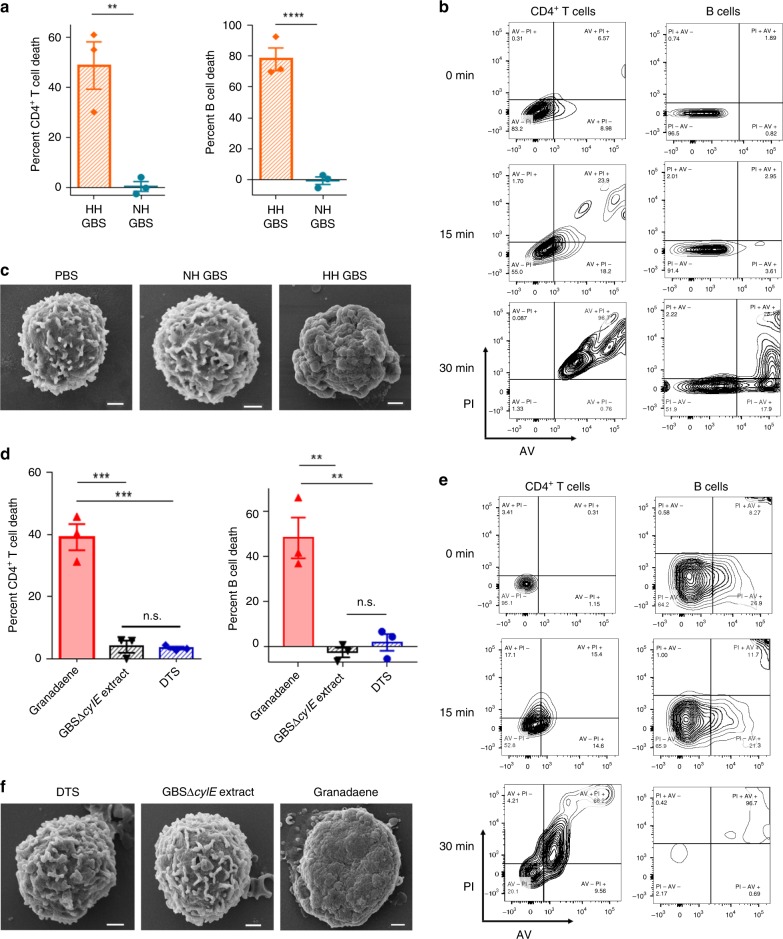
Granadaene is cytolytic to T and B cells. **a** Primary human CD4^+^ T and B cells were incubated with either hyper-hemolytic GBS (HH GBS) or non-hemolytic GBS (NH GBS) at an MOI of 10 for 1 h at 37 °C, and cytotoxicity was measured by the release of lactate dehydrogenase (LDH) into the cell supernatant relative to 100% lysis (Triton X-100 (0.1%)) and 0% lysis (PBS) controls. Mean and standard error from three independent experiments are shown. A two-tailed unpaired *t* test was used to compare groups. CD4^+^ T cells: *p* = 0.0075, B cells: *p* = 0.0005. **b** PI uptake and Annexin V staining were measured using flow cytometry following incubation with HH GBS at an MOI of 10 at the indicated time points. **c** CD4^+^ T cells were imaged using scanning electron microscopy (SEM) following incubation with PBS, NH GBS, or HH GBS (MOI = 10) for 1 h. Images are representative of three experiments. Scale bars are 1 μm. **d** Primary human CD4^+^ T and B cells were incubated with purified granadaene (0.5 µM), equivalent amount of extract from NH GBS (GBSΔ*cylE*), or solvent (DTS), and cell death was measured by LDH release in supernatants, as above. Mean and standard error from three independent experiments are shown. Groups were compared with one-way ANOVA with Tukey’s post test. CD4^+^ T cells: granadaene vs. Δ*cylE* extract: *p* = 0.0002, granadaene vs. DTS: 0.0002, Δ*cylE* extract vs. DTS: *p* = 0.9928. B cells: granadaene vs. Δ*cylE* extract: *p* = 0.0020, granadaene vs. DTS: 0.0032, Δ*cylE* extract vs. DTS: *p* = 0.8513. **e** PI uptake and Annexin V staining were measured using flow cytometry in each cell type following incubation with purified granadaene (0.5 µM) at the indicated time points. **f** CD4^+^ T cells were imaged using SEM following incubation with DTS, GBSΔ*cylE* extract, or granadaene (0.5 µM) for 1 h. Images are representative of three experiments. Scale bars are 1 μm. **, *p* < 0.01. ***, *p* < 0.001. ****, *p* < 0.0001. n.s., Not significant.

First, primary CD4^+^ T or B cells isolated from the blood of healthy adult humans were incubated with GBS that were either hyper-hemolytic (NCTC10/84, serotype V) or non-hemolytic (NCTC10/84Δ*cylE*; the non-hemolytic strain lacks the *cylE* gene, which is necessary for granadaene production^31^) at a multiplicity of infection (MOI) of 10 for 1 h. We noted that the hyper-hemolytic strains induced cell death, which was significantly greater compared to cells treated with non-hemolytic GBS (Fig. 3a). These findings indicate that adaptive immune cells are susceptible to death by hyper-hemolytic GBS strains. Of note, only minimal cytotoxicity (<10%) was observed with modestly hemolytic GBS strains (A909, serotype Ia; and COH1, serotype III), similar to our previous observations with innate immune cells^7,24,26^. Next, to explore the mechanism and kinetics of cell death, we measured propidium iodide (PI) uptake and Annexin V (AV) staining in both cell types at 0, 15, and 30 min following incubation with hyper-hemolytic GBS at an MOI of 10. We found that at 0 min, most cells were negative for both PI and AV, but after just 15 min of incubation, a proportion of each cell type were positive for PI and AV, and by 30 min, more cells became positive for both PI and AV (Fig. 3b). The rapid phenotypic shift of PI positive/AV negative (PI−/AV−) to PI positive/AV positive (PI+/AV+) suggests that CD4^+^ T and B cells underwent a lytic form of cell death^32^. Notably, cells remained PI−/AV− after 30 min of treatment with non-hemolytic GBS (Supplementary Fig. 2). When CD4^+^ T cells were imaged using scanning electron microscopy (SEM) after exposure to hyper-hemolytic GBS, non-hemolytic GBS, or phosphate-buffered saline (PBS), striking changes in cell surface morphology were observed in cells treated with hyper-hemolytic GBS, but not with cells exposed to non-hemolytic GBS or saline controls (PBS) (Fig. 3c).

To determine if granadaene alone was sufficient for induction of these cytolytic effects, the above assays were performed with purified granadaene (0.5 µM), equivalent amount of extract from non-hemolytic GBS (GBSΔ*cylE* extract), or solvent (DTS) alone. Similar to our observations with live bacteria, significantly greater cell death was observed when CD4^+^ T and B cells were exposed to granadaene compared to the controls (GBSΔ*cylE* extract or DTS) (Fig. 3d), and these cells also rapidly switched to PI+/AV+ (Fig. 3e), while the vast majority of controls remained PI−/AV− (Supplementary Fig. 2). Further, SEM revealed that CD4^+^ T cells treated with granadaene exhibited morphological changes on the cell surface, unlike cells treated with GBSΔ*cylE* extract or DTS (Fig. 3f). Together, these findings demonstrate that granadaene and hyper-hemolytic GBS strains are cytotoxic to cells of the adaptive immune system.

### Synthetic analog R-P4 is not toxic to T and B cells

**Fig. 4:**
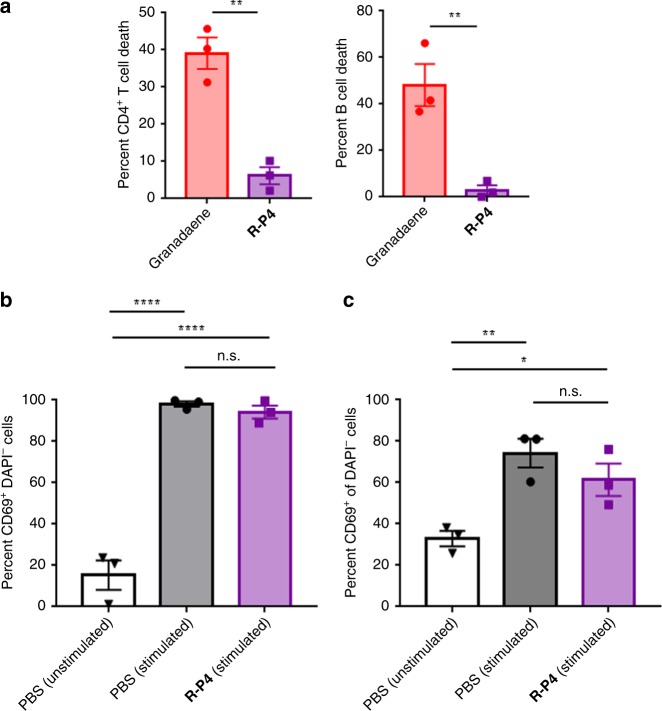
R-P4 is non-toxic to T and B cells. **a** Primary human CD4^+^ T cells (left) and B cells (right) were incubated with **R-P4** (20 μM) or granadaene (0.5 μM) for 1 h at 37 °C, and cytotoxicity was measured by LDH release into cell supernatant relative to 100% lysis (Triton X-100 (0.1%)) and 0% lysis (PBS) controls. Mean and standard error from three independent experiments are shown. Groups were compared with a two-tailed unpaired *t* test. CD4^+^ T cells: *p* = 0.0024, B cells: *p* = 0.0084. **b** Primary human CD4^+^ T cells were treated with PMA (10 ng/mL) and anti-CD3ε (100 μg/mL) (stimulated) or media alone (unstimulated). Then, cells were treated with either PBS or **R-P4** (20 μM) and incubated at 37 °C for 48 h. Cells were stained for CD69, resuspended in DAPI (0.5 μM), and analyzed by flow cytometry. Percent CD69^+^ of DAPI− cells from three independent experiments are represented with mean and standard error. Treatment groups were compared using one-way ANOVA with Tukey’s post test. PBS (unstimulated) vs. PBS (stimulated): *p* < 0.0001, PBS (unstimulated) vs. **R-P4** (stimulated): *p* < 0.0001, PBS (stimulated) vs. **R-P4** (stimulated): *p* = 0.8151. **c** Primary human B cells were treated with IL-4 (20 ng/mL) and anti-CD40 (5 μg/mL) (stimulated) or media alone (unstimulated). Then, cells were treated with either PBS or **R-P4** (20 μM) and incubated at 37 °C for 48 h. Cells were stained for CD69, resuspended in DAPI (0.5 μM), and analyzed by flow cytometry. Percent CD69^+^ of DAPI− cells from three independent experiments are represented with mean and standard error. Treatment groups were compared using one-way ANOVA with Tukey’s post test. PBS (unstimulated) vs. PBS (stimulated): *p* = 0.0091, PBS (unstimulated) vs. **R-P4** (stimulated): *p* = 0.0453, PBS (stimulated) vs. **R-P4** (stimulated): *p* = 0.3893. *, *p* < 0.05. **, *p* < 0.01. ****, *p* < 0.0001. n.s., Not significant.

Our results above indicate that granadaene is likely to be a poor candidate for incorporation into a vaccine for prevention of GBS infection. We reasoned that a non-toxic synthetic analog of the GBS ornithine rhamnopolyene may be better suited in a vaccine formulation. Because **R-P4** was non-hemolytic and contains structural components similar to granadaene of all the synthesized analogs (i.e. terminal rhamnose, polyene chain, and terminal amino acid), we tested its cytotoxicity to CD4^+^ T and B cells. We found that primary human CD4^+^ T or B cells exposed to **R-P4** (20 μM) for 1 h showed minimal cell death (Fig. 4a). Next, we determined whether CD4^+^ T and B cells could respond to activation stimuli in the presence of **R-P4**. To this end, primary human CD4^+^ T cells were stimulated with PMA (phorbol 12-myristate 13-acetate) and αCD3ε, and primary human B cells were stimulated with αCD40 and interleukin-4 (IL-4). Cells were then treated with PBS, **R-P4** (20 μM), or purified granadaene (5 μM). After 48 h, cells were stained for the canonical activation marker CD69^33^, resuspended in DAPI (2-[4-(aminoiminomethyl)phenyl]-1*H*-indole-6-carboximidamide hydrochloride) as a viability stain, and analyzed by flow cytometry (see gating strategy in Supplementary Fig. 3). Compared to unstimulated controls treated with PBS, stimulated CD4^+^ T and B cells exposed to **R-P4** up-regulated CD69 (Fig. 4b, c). Furthermore, CD69 expression in stimulated cells exposed to **R-P4** was no different than stimulated cells exposed to PBS (Fig. 4b, c). Of note, <10% of cells treated with granadaene were viable (DAPI−) (Supplementary Fig. 3), so this group was excluded from the CD69 analysis in both cell types. However, the proportion of DAPI− cells was no different among other treatment groups in either cell type (Supplementary Fig. 3). Collectively, these results indicate that CD4^+^ T and B cells survive and respond to activating stimuli when exposed to **R-P4**.

### Vaccination with R-P4 analog diminished GBS infection

**Fig. 5:**
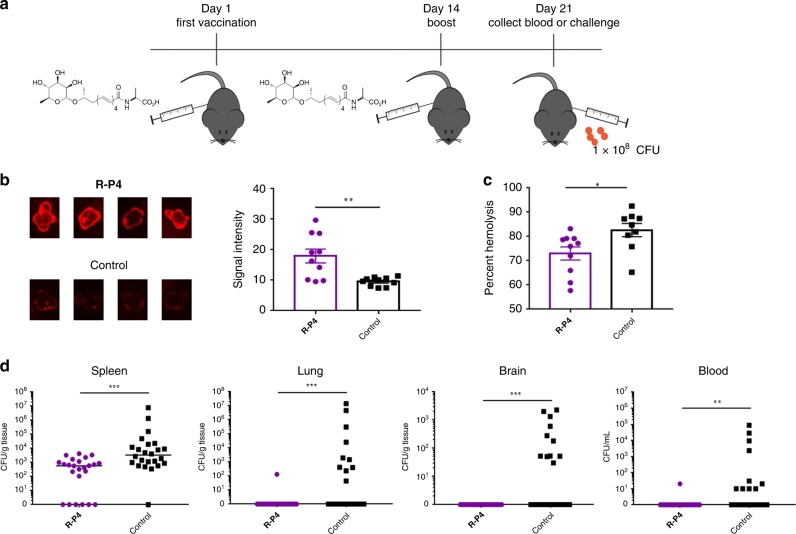
Vaccination with a non-toxic synthetic analog diminished GBS infection. **a** Mice were vaccinated with an emulsion of **R-P4** (10 μM in PBS) in complete Freund’s Adjuvant and boosted 14 days after initial vaccination using **R-P4** in incomplete Freund’s Adjuvant. At 21 days, vaccinated mice were euthanized for blood/plasma collection or were challenged (IP) with ∼1 × 10^8^ CFU of hyper-hemolytic GBS strain NCTC10/84. As controls, mice receiving adjuvant only were tested in parallel with the same schedule. **b** Approximately 4 μL solvent or purified granadaene (25 μM) was spotted on PVDF membranes, which were then blocked and probed with plasma from analog-vaccinated or adjuvant-only mice (*n* = 10/group). Subsequently, the membranes were probed with secondary goat anti-mouse IgG (Alexa Fluor 680) and analyzed on the Odyssey LI-COR Imaging System. Four representative spots from each group are shown. Signal intensity of each spot was determined using the Image J software, and a two-tailed unpaired *t* test was used to compare signal intensity between groups (*p* = 0.0020). Mean and standard error are shown. **c** Plasma (diluted 1:1000) from analog-vaccinated mice or adjuvant-only only mice (*n* = 10/group) was pre-incubated with purified granadaene (0.3 μM) for 1 h and then incubated with human red blood cells for 1 h. Percent inhibition of granadaene hemolysis was calculated (see Methods), and a two-tailed unpaired *t* test was used to compare hemolysis inhibition by **R-P4** plasma vs. control plasma (*p* = 0.0004). Mean and standard error are shown. **d** GBS CFU recovered from the blood, spleen, lung, and brain of mice (*n* = 24) at 24 h post infection were compared using a two-tailed Mann–Whitney test. Blood: *p* = 0.0025, spleen: *p* = 0.0001, lung: *p* = 0.0010, brain: *p* = 0.0002. *, *p* < 0.05. **, *p* < 0.01. ***, *p* < 0.001.

Next, we hypothesized that vaccination with **R-P4** may generate an immune response that antagonizes the effects of granadaene during systemic GBS infection. Given that hyper-hemolytic GBS cause lethal infection in adult mice^34^, we used the adult systemic model of GBS infection to test if **R-P4** could diminish infection with a hyper-hemolytic strain. To this end, mice were immunized (intraperitoneally (IP)) with an emulsion of **R-P4** (10 μM in PBS) in complete Freund’s adjuvant and boosted 14 days later with the same dose of **R-P4** in incomplete Freund’s adjuvant (see Methods). On day 21 after initial vaccination, mice were euthanized for plasma collection or were challenged (IP) with the hyper-hemolytic GBS strain NCTC10/84 (Fig. 5a). As controls, mice were injected with adjuvant alone on the same vaccination schedule.

Immunoblots revealed that plasma from mice vaccinated with **R-P4** contained more immunoglobulin G (IgG) bound to purified granadaene compared to adjuvant-only controls (Fig. 5b). Furthermore, plasma from analog-vaccinated mice inhibited granadaene-mediated hemolysis in contrast to plasma from adjuvant-only controls (Fig. 5c, *n* = 10/group). The amount of granadaene-bound IgG detected in plasma significantly correlated with inhibition of granadaene hemolysis (Supplementary Fig. 4), suggesting that granadaene-reactive IgG partially neutralizes toxin function. At 24 h post-bacterial challenge, mice were euthanized and GBS colony-forming units (CFU) were enumerated in the spleen, lung, brain, and blood. Significantly fewer CFUs were recovered from the tissues and blood of analog-vaccinated mice compared to adjuvant-only control mice (*n* = 24/group) (Fig. 5d), indicating that immunization with **R-P4** limited bacterial dissemination. Together, these data demonstrate that vaccination with a non-toxic granadaene analog generates granadaene-specific antibodies with neutralizing properties and protects against infection with hyper-hemolytic GBS.

## Discussion

Microbial lipids produced by several human pathogens, including mycolactone from *Mycobacterium ulcerans*^1,2^, rhamnolipids from *Pseudomonas aeruginosa*^3,4^, commendamide from *Bacteroides* spp.^5^, and granadaene from GBS^6,7^, contribute to pathogenesis by killing host cells. Yet, structure–function studies demonstrating the underpinnings of cytotoxicity in such compounds are lacking, and no strategies exist to attenuate toxin activity during infection. Here, we use chemical synthesis to understand the characteristics important for hemolytic activity in the ornithine rhamnopolyene produced by GBS. Furthermore, we provide proof of concept for a vaccine that mitigates the effect of this lipid toxin during infection.

Our studies indicate that the polyene chain length is a key factor in the ability of granadaene to lyse RBCs. We show that lipids with one or four alkenes (**pP1** and **R-P4**) are not hemolytic despite having a terminal amino acid and protecting group (Supplementary Fig. 1). Similarly, polyenes containing seven double bonds were non-hemolytic (**P7**) unless the terminal polar group was replaced with a hydrophobic group such as TBS (**pP7X**, **pP7)**. In contrast, lipids with nine alkenes (**P9**, **pP9**) were hemolytic, with or without the TBS-protecting group (Fig. 1). Interestingly, a much greater concentration of each hemolytic analog was required for activity (62.5–250 μM, Fig. 1) compared to that previously observed for granadaene (0.1–1 μM)^6^. We predict that this may be due to the length of the polyene chains; the predicted length of granadaene (>32 Å) is longer when compared to the length of each analog (<24 Å, Table 1). Our data suggest that granadaene’s hydrophobic polyene moiety can span the thickness of the target cell’s plasma membrane (30–40 Å)^7,35–37^, with the terminal ornithine and rhamnose serving as hydrophilic polar head groups that allow for stable insertion. The ability of the polyene moiety of **pP7X**, **pP7, P9**, and **pP9** to span the plasma membrane partially, but not entirely, could explain the observed attenuated hemolytic activity relative to granadaene. Additionally, calculated partition coefficients for more active lipids are in agreement with a dynamic and favorable lipophilic interaction with lipidic cellular domains^38^.

The observation that **P7**, which is similar in length to **pP7**, is non-hemolytic (Supplementary Fig. 1) suggests an additional role for the terminal head groups in hemolytic activity. It is plausible that the terminal, hydrophilic hydroxyl group in **P7** being imbedded within the highly hydrophobic lipid bilayer destabilizes the seven-alkene analog, whereas the hydrophobic TBS-protecting group in **pP7** allows for stable insertion into the cell’s lipid bilayer. Our finding that **P9**, which does not have a TBS group but a terminal hydroxyl group, is hemolytic (Fig. 1e) suggests that a longer polyene chain (i.e., nine alkenes versus seven alkenes) stabilizes insertion of the polyene into the cell membrane. A limitation of our work is the present inability to synthesize compounds with polyene chains containing more than nine alkenes due to rapid denaturation during synthesis. Synthesis of analogs with longer polyenes, such as those with 12 double bonds like granadaene, will be essential in confirming the model we propose.

Recently, microbial lipids have gained appreciation for their importance as antigens in the host response against bacterial infections^39^. Here, we show that granadaene kills cells of the adaptive immune system, including CD4^+^ T and B cells (Fig. 3). Death in these cells was marked by cell membrane permeability and damage, as indicated by rapid PI/AV positivity and SEM imaging. Our findings with adaptive immune cells could at least partially explain why antibodies specific to granadaene have never been isolated from infected patients or produced in the laboratory; the cytotoxic effect of the GBS ornithine rhamnopolyene to the very cells involved in antibody production inhibit their function. As such, granadaene is likely a poor candidate to serve as an antigen in a GBS vaccine formulation.

We hypothesized that a non-toxic analog with structural similarities to granadaene, namely the rhamnose, polyene chain, and amino acid, may serve as an antigen that would prompt an immune response with cross-reactivity to granadaene during GBS infection. We show that compound **R-P4** is non-toxic to CD4^+^ T and B cells and that these cells can respond to activating stimuli when exposed to **R-P4** (Fig. 4). Thus, we selected **R-P4** as the immunizing agent for our vaccination studies. We found that plasma from mice vaccinated and boosted with **R-P4** contained class-switched antibodies that bound specifically to granadaene (Fig. 5b). In addition, plasma from **R-P4**-vaccinated mice inhibited hemolytic activity of granadaene ex vivo more than plasma from control mice (Fig. 5c). Moreover, the amount of granadaene-bound IgG in plasma significantly correlated with granadaene inhibition (Supplementary Fig. 4). These findings suggest that vaccination with **R-P4** generated granadaene-specific antibodies, which had toxin-neutralizing properties. The anti-virulence factor effect of **R-P4** vaccination was further supported by results from infected mice; analog-vaccinated mice infected with hyper-hemolytic GBS experienced less bacteremia and bacterial dissemination to peripheral organs, including the spleen, lungs, and brain compared to adjuvant-only control mice (Fig. 5c). Because granadaene has been shown to promote GBS transmigration of the lung and blood brain barriers^25,40^, our results suggest that vaccination with **R-P4** limited GBS virulence by counteracting the pathogenic effects of this toxin. We hypothesize that the high degree of structural similarity between **R-P4** and granadaene allowed for the production of antibodies with affinity for granadaene that dampened the activity of this toxin during GBS infection. Additional data on the dynamics of the humoral and cell-mediated response during immunization and the course of infection will lend greater insight into the mechanisms of protection conferred by this vaccine formulation and will inform the design of other vaccines that incorporate lipid antigens.

In conclusion, our findings with synthetic analogs reveal the structural components of granadaene that are important for host cell lysis. Additionally, we overcome granadaene-mediated cytotoxicity of T and B cells by creating a vaccine using a non-toxic granadaene analog, which prompted the generation of granadaene-binding antibodies and reduced the harmful effects of this toxin during GBS infection. Collectively, these studies provide proof of concept for a strategy to counteract a cytotoxic microbial polyene that is critical to pathogenesis. These findings have broad application to understanding the biological activity of similar microbial lipid toxins and the development of targeted therapeutics against them.

## Methods

### Ethics statement

Written informed patient consent for donation of human blood was obtained with approval from the Seattle Children’s Research Institute Institutional Review Board (protocol #11117) per the Principles in the WMA Declaration of Helsinki and Department of Health and Human Services Belmont Report. Children under the age of 18 years were not recruited for donation of human blood.

All animal experiments were approved by the Seattle Children’s Research Institutional Animal Care and Use Committee (protocol ID IACUC00036) and performed in strict accordance with the recommendations in the Guide for the Care and Use of Laboratory Animals of the National Institutes of Health (8th Edition).

### Chemicals

DMSO (Fisher Scientific), TFA (Thermo Fisher Scientific), and Difco soluble starch (BD) were used to make DTS. All reagents used in the synthesis of granadaene-inspired lipids were purchased from standard chemical suppliers and used without further purification.

### Bacterial strains

The wild-type (WT) GBS strains used in this study are A909 (serotype Ia) and NCTC10/84 (serotype V); these strains are clinical isolates obtained from infected newborns^41^. Isogenic non-hemolytic Δ*cylE* mutants derived from A909 and NCTC10/84^31^ were used as non-hemolytic controls.

Cultures of GBS were grown in tryptic soy broth (Difco Laboratories) at 37 °C in 5% CO_2_. Culture growth for all bacterial strains was measured at 600 nm, and bacterial strains were washed twice in PBS before being used in experiments. Photographs of bacterial strains on blood agar (Remel) or Granada medium (Hardy Diagnostics) were captured with an SLR camera (EOS Rebel XSi 12.2MP; Cannon) with an 18–55 mm zoom lens and processed using Photoshop CC (Adobe).

### Isolation and purification of granadaene from GBS

Granadaene was isolated from WT GBS A909 as described^6,18,42^. Approximately 500 mL cultures of WT GBS A909 cultures were grown in Granada medium at 37 °C, in 5% CO_2_. Bacterial cells were pelleted, washed three times with distilled water, and twice with DMSO. The cell pellet was then resuspended in DMSO:0.1% TFA (Sigma-Aldrich) overnight to extract granadaene, cell debris was pelleted, and the supernatant containing grandaene was saved. The above process was repeated until the supernatant obtained from GBS cells was clear. Granadaene was then precipitated by the addition of 25% NH_4_OH (Scientific Products) to a final concentration of 0.25%. Precipitated granadaene was washed three times with high pressure liquid chromatography (HPLC) grade water and twice in DMSO before redissolved in DMSO:0.1%TFA. Granadaene was then column purified using HPLC with a Vydac 214TP C4 column. Purified fractions were pooled, precipitated with NH_4_OH, washed twice with HPLC grade water, and then twice with DMSO, and lyophilized to dryness. Lyophilized granadaene was stored at −80 °C, and working granadaene solutions were dissolved in DMSO + 0.1% TFA + 20% starch (DTS) as needed. The purification and isolation procedure were also performed on GBS A909Δ*cylE* (non-pigmented/non-hemolytic isogenic mutant of WT A909). Extract from A909Δ*cylE* was used as a control for granadaene in all experiments, along with the DTS solvent. ^1^H NMR spectrum of purified samples was used as a criteria of purity and identity.

### Synthesis of GBS granadaene-inspired lipids

The entire procedure for the synthesis of granadaene-inspired lipids is provided in Supplementary Materials.

General information: The synthetic approach was based on an iterative sequence of Horner–Wadsworth–Emmons olefination reactions. Although many other olefination reactions are known, this sequence was chosen for its preference toward *E* stereoisomers, which is desirable for synthesis of the target compounds. The complexity of the analysis of naturally occurring granadaene precluded an exact analysis of the stereochemistry of the polyene chain, which was postulated to be *E*. The stereochemical purity in such a long polyene is compromised and therefore many diastereoisomers could contribute to the hemolytic activity. Given this, we produced a mixture of diastereoisomers composed by the all *E*-diastereoisomer and minor amounts of other *Z*-containing diastereoisomers, thus more precisely mimicking natural granadaene.

Tetrahydrofuran (THF) was freshly distilled from Na. CH_2_Cl_2_ was freshly distilled from P_2_O_5_. Thin layer chromatography was performed on aluminum-backed plates coated with silica gel 60 (230–240 mesh) with F_254_ indicator. The spots were visualized with ultraviolet light (254 nm). All chromatography purifications were performed with silica gel 60 (40–60 μm). NMR spectra were measured at room temperature. ^1^H NMR spectra were recorded at 400, 500, and 600 MHz. Chemical shifts are reported in p.p.m. using residual solvent peak as reference (CHCl_3_: δ = 7.26 p.p.m., CH_3_OH: δ = 3.31 p.p.m., DMSO: δ = 2.50 p.p.m.). ^13^C NMR spectra were recorded at 100, 125, and 150 MHz using broadband proton decoupling, and chemical shifts are reported in p.p.m. using residual solvent peaks as reference (CHCl_3_: δ = 77.16 p.p.m., CH_3_OH: δ = 49.0 p.p.m., DMSO: δ = 39.51 p.p.m.). Carbon multiplicities were assigned by DEPT (distortionless enhancement of polarisation transfer) techniques. High-resolution mass spectra were recorded on a mass spectrometer using electron ionization at 70 eV. Racemic ethyl 3-hydroxybutanoate was used in the synthesis of all granadaene derivatives, except for **R-P4** owing to the presence of the d-rhamnose subunit, which can generate different diastereosiomers. In this case, (*3R*)-ethyl 3-hydroxybutanoate was used as the starting material. The known compound ethyl 3-((*tert*-butyldimethylsilyl)oxy)butanoate was isolated as pure sample and showed NMR spectra matching those of previously reported^43–45^.

General procedure for Horner–Wadsworth–Emmonds reaction (GP-1): A mixture of the corresponding phosphonate (2.5 mmol), and NaH (2.5 mmol, 60% purity) in THF (15 mL) was stirred at 0 °C for 10 min Then, the corresponding aldehyde (1 mmol) was added dropwise for 10 min, and the mixture was stirred at room temperature for 0.5–1.5 h. Then, saturated aqueous NH_4_Cl was added, and THF was removed under reduced pressure. The residue was solved in EtOAc, and the mixture was washed with saturated aqueous NH_4_Cl and brine, dried over anhydrous Na_2_SO_4_, and the solvent was removed. Products were purified by flash chromatography on silica gel (EtOAc/Hexane mixtures) and characterized by spectroscopic techniques.

General procedure for DIBAL-H reduction of esters to alcohols (GP-2): To a solution of the corresponding ester (1 mmol) in THF (5 mL), DIBAL-H (2.5 mmol, 1 M in THF) was added, and the mixture was stirred at 0 °C for 1–2 h. Then, the reaction was quenched with H_2_O, diluted with EtOAc, and washed with 10% solution of HCl and brine. The mixture was dried over anhydrous Na_2_SO_4_ and the solvent removed. Products were purified by flash chromatography on silica gel (EtOAc/hexane mixtures) and characterized by spectroscopic techniques.

General procedure for oxidation with Dess–Martin periodinane (GP-3): To a solution of the corresponding alcohol (1 mmol) in CH_2_Cl_2_ (15 mL) at 0 °C, Dess–Martin periodinane (1.5 mmol) was added, and the mixture was stirred at room temperature for 1–2 h. Then, the solvent was removed, and EtOAc was added. The organic layer was washed with a 1:1 solution of saturated NaHCO_3_ and 10% Na_2_S_2_O_3_, dried over anhydrous Na_2_SO_4_, and the solvent removed. Products were purified by flash chromatography on silica gel (EtOAc/hexane mixtures) and characterized by spectroscopic techniques.

General procedure for basic deprotection of esters (GP-4): To a solution of the corresponding ester (1 mmol) in wet MeOH (30 mL), a 2 M solution of KOH in MeOH (8–12 mmol) was added, and the mixture was stirred at room temperature for 4–6 h. Then, a few drops of water were added, and the mixture was neutralized by the addition of amberlyst 15 (110 mg, previously washed with CH_2_Cl_2_ and MeOH). Products were purified by flash chromatography on silica gel (CH_2_Cl_2_/MeOH) and characterized by spectroscopic techniques.

The ^1^H and ^13^C NMR spectra of synthetic lipids and precursors are shown in Supplementary Fig. 5.

### Modeling granadaene and synthetic lipids

All compounds were drawn with the ChemDraw software (PerkinElmer). Optimized structures were obtained using Chem3D software (MM2 level) (PerkinElmer), and were visualized as .mol files using Mercury software (The Cambridge Crystallographic Data Center). Here, the distance between the first carbon to the last carbon of the last double bond in the polyene chain was measured to calculate the length of the polyene chain for each compound.

### Hemolytic assays

Synthetic analogs (Table 1) were resuspended in DTS or DMSO to achieve a concentration of 0.02 M. To test for hemolysis, 3–5 µL of synthetic analogs were spotted onto a Red blood agar plate (TSA plate containing 5% sheep’s blood, Remel). After allowing the spots to dry for about 10 min at room temperature, the blood agar plates incubated overnight at 37 °C in 5% CO_2_. Blood agar plates were placed on a light box, and photographs were captured with the digital SLR camera described above and processed using Photoshop CC (Adobe). Synthetic analogs were co-incubated with human RBCs (1%) in PBS in a final volume of 100 µL at 250, 125, and 62.5 µM for 1 h at 37 °C, after which the samples were spun for 4 min at 3000 × *g* to pellet unlysed RBCs. Hemoglobin release was measured by recording the absorbance at 420 nm of the supernatants obtained from above. Percent hemolysis relative to Triton X-100 (0.1%)-treated positive controls and PBS-treated negative controls was calculated.

### Isolation of T and B cells from human adult blood

Approximately 15 mL of blood was collected from healthy human adults into EDTA tubes (BD Biosciences). Immediately following collection, CD4^+^ or B cells were isolated from the blood using the appropriate RosetteSep Enrichment Cocktail (StemCell) along with SepMate tubes (StemCell), as per the manufacturer’s instructions. Cells were then pelleted, and any residual RBCs were removed by re-suspending the cell pellet in RBC lysis solution (150 mM NH_4_Cl, 1 mM NaHCO_3_) for 15 min at room temperature. Following RBC lysis, cells were washed with Roswell Park Memorial Institute 1640 tissue culture medium containing l-glutamine (Corning; hereafter referred to as RPMI-G). Cell purity was assessed by examining the proportion of cells positive for the appropriate markers using flow cytometry. Briefly, ~1 × 10^6^ cells from the purification preparation or 1 × 10^6^ cells from whole blood (following two RBC lysis steps, as described above) were incubated with Fc receptor block (1:200; BD Biosciences) for 15 min at room temperature. Then, immunofluorescent antibodies were added to the cells and cells were incubated for 30 min at room temperature. B cell preparations were stained with CD19-PerCP/Cy5.5 (3:100; BioLegend); CD4^+^ T cell preparations were stained with CD3-FITC (1:10; BD Biosciences), CD4-V450 (1:10; BD Biosciences), and CD8-PerCP/Cy5.5 (1:10; BioLegend). Stained cells were washed twice in FACS (fluorescence-activated cell sorting) buffer (1 mM EDTA, 25 mM HEPES, 1% bovine serum albumin (w/v) in PBS) and were analyzed immediately on an LSR II flow cytometer (BD Biosciences). Single-stained fluorochrome-reactive AbC beads (Thermo Fisher) and unstained cells were used for compensation. Data were analyzed using FlowJo v. 10.1 (FlowJo, LLC).

### Analysis for cytotoxicity

T and B cells were isolated as described above. T or B cells were seeded with 2.5 × 10^6^ cells in each well in 90 µL RPMI-G. Hyper-hemolytic GBS (WT NCTC10/84) and non-hemolytic GBS (NCTC10/84Δ*cylE*) were grown to mid-exponential growth phase (OD_600_ = 0.3), washed twice in sterile PBS, and added to cells at a multiplicity of infection (MOI) of 10. MOIs were confirmed by dilution plating. Additionally, granadaene in DTS from WT GBS was added to cells at a final concentration of 0.5 µM, and **R-P4** was added to cells at a final concentration of 20 μM. As positive and negative controls for all assays, cells were incubated in 0.1% Triton X-100 (Sigma-Aldrich) or sterile PBS, respectively. After 1 h incubation at 37 °C, cells were analyzed for cytotoxicity by the presence of cytoplasmic lactate dehydrogenase (LDH) in cell supernatants using the Colorimetric LDH kit (Clontech), as per the manufacturer’s instructions. Percent cytotoxicity was calculated by normalizing to PBS-treated cells (0% cell death) and Triton X-100-treated cells (100% cell death), as described^6,7,24^.

### Uptake of PI and AV

PI uptake and AV staining were measured concurrently as described^24^. To this end, isolated T and B cells from above were washed in PBS and resuspended in AV binding buffer (10 mM HEPES, 140 mM NaCl, 2.5 mM CaCl_2_ in PBS) at a concentration of ~3.33 × 10^6^ cells/mL. Cells were then incubated with AV-Alexa Fluor 488 (1:20; Invitrogen) and PI (12.5 µg/mL; Life Technologies) for 15 min at room temperature, protected from light. Cells were then diluted by a factor of 5 in AV binding buffer into FACS tubes (BD Biosciences) and treated with hyper-hemolytic GBS or non-hemolytic GBS at an MOI of 10 or granadaene (0.5 µM) or an equivalent volume of GBSΔ*cyl*E extract. PI uptake and AV staining were measured using an LSR II flow cytometer (BD Biosciences) immediately following inoculation (0 min time point) and various times after inoculation (15, 30 min). Gates for PI+ and AV+ cells were determined using unstained and single-stained controls.

### Scanning electron microscopy

Samples were processed for SEM by the Electron Microscopy Core at Fred Hutch Cancer Research Center as before^6,24,26^. To this end, cells were washed in PBS and resuspended in one volume of 1/2 Karnovsky’s fixative (2% paraformaldehyde, 2.5% glutaraldehyde, 2.5 mM CaCl_2_ in 0.1 M cacodylate buffer, pH 7.2) and then were laid down on a poly-l-lysine coverslip for 30 min The coverslip with cells were washed in 0.1 M cacodylate buffer three times for 10 min each. The cells were post fixed with OsO_4_ for 2 h at 4 °C and then washed in 0.1 M cacodylate buffer three times for 10 min each. Samples were dehydrated using EtOH at 50%, 70%, and 95% for 25 min each, followed by two changes of 100% EtOH for 20 min. The cells were then treated with 1/2 volume 100% EtOH and 1/2 volume HMDS (hexamethyldisilazane) for 20 min twice and 100% HMDS twice for 20 min each. Using fresh HMDS, the coverslips were slightly covered and allowed to dry overnight or until the HMDS had evaporated. Specimens were mounted onto a stub containing carbon conductive tabs and sputter coated with a gold-palladium target, and images were obtained using a JEOL JSM 6610LV with JEOL Acquisition System software.

### Stimulation of CD4^+^ T and B cells

Primary human CD4^+^ T and B cells were isolated from the blood of healthy human adults, as described above. Cells were seeded at ~1 × 10^6^ cells/mL in RPMI-G on a TC-treated 96-well plate (180 μL in each well). CD4^+^ T cells were stimulated with immobilized anti-human CD3ε (0.5 μg; BD Biosciences) and PMA (10 ng/mL; Sigma), as described^46,47^. B cells were stimulated with human IL-4 (20 ng/mL; Sigma) and anti-human CD40 monoclonal antibody (5 μg/mL; Enzo, clone mAb 89), as described^48^. Immediately following stimulation, cells were treated with either PBS or **R-P4** (20 μM) in technical triplicate. As controls, a group of CD4^+^ T and B cells received no stimulus and no treatment (designated as “PBS (unstimulated)”). After incubating for 48 h at 37 °C, cells were treated with human Fc block (1:200; BD Biosciences), stained with anti-CD69-PE/Cy7 (10 μL/test; BD Biosciences, clone FN50), washed, and resuspended in DAPI (0.5 μM; Thermo). Cells were run on an LSR II flow cytometer (BD Biosciences). Unstained cells, single-stained DAPI+ cells, and single-stained PE/Cy7+ beads were used as compensation controls. Data were analyzed using the FlowJo software, and gates for DAPI− and CD69+ events were determined using fluorescence minus one or unstained controls. For the gating strategy used to calculate percent CD69^+^ cells of single cells, see Supplementary Fig. 5.

### Vaccination

The first vaccine emulsion was prepared by mixing Complete Freund’s Adjuvant (Invivogen) and **R-P4** (20 μM dissolved in sterile PBS) at a ratio of 1:1. For the first vaccination, 100 μL of the emulsion was injected (IP) into male (*n* = 12) and female (*n* = 12) C57BL6/J mice (obtained from Jackson Laboratories). Fourteen days after initial vaccination, mice were injected with 100 μL of freshly prepared vaccine emulsion in Incomplete Freund’s Adjuvant (Invivogen). Adjuvant-only emulsions were made by mixing the appropriate adjuvant in sterile PBS at a ratio of 1:1. Adjuvant-only control mice were injected (IP) in male (*n* = 12) and female (*n* = 12) mice on the same schedule as analog-vaccinated mice.

### Immunoblots

Blood was collected from 10 vaccinated mice and 10 adjuvant-only control C57BL6/J mice via cardiac puncture 21 days after initial vaccination. Blood was centrifuged at 3000 x *g* for 15 min at 4 °C, and unpelleted plasma was collected and stored at −80 °C.

Granadaene (dissolved in DMSO + 0.1% trifluoroacetic acid) was diluted to 25 μM in PBS and was pipetted (4 μL) onto pre-cut squares of Immoblion-FL PVDF membrane (Sigma-Aldrich). After drying for ~1 h, membranes were treated with Odyssey blocking buffer in PBS (LI-COR) for 1 h while shaking at room temperature. Blocking buffer was removed, and each membrane was probed with an aliquot of plasma from an analog-vaccinated or adjuvant-only mouse diluted 1:250 in buffer solution (1:1 Odyssey blocking buffer and PBS with 0.02% Tween-20). After shaking overnight at 4 °C, membranes were washed three times in wash solution (TBS + 0.02% Tween-20), and then probed with Alexa Fluor 680 goat anti-mouse IgG (Invitrogen) (1:2500 in buffer solution). Membranes incubated with secondary antibody for 45 min at room temperature were protected from light. Then, membranes were washed three times in wash solution and twice in PBS and imaged using the LI-COR Odyssey Infrared Imaging System. Signal intensity of each spot was determined using the Image J software.

### Granadaene ex vivo inhibition assay

Diluted plasma (1:1000) from analog-vaccinated mice or adjuvant-control mice (*n* = 10/group) were pre-incubated with purified granadaene (0.3 μM in DTS) for 1 h at room temperature. The samples were then incubated with 100 µL of EDTA-treated human red blood cells (1% in PBS) for 1 h at 37 °C. As described above, hemoglobin release in cell supernatants was measured using the SpectraMax i3x plate reader (absorbance at 420 nm), and percent hemolysis relative to Triton X-100 (0.1%)/granadaene-treated positive controls (100% hemolysis) and PBS-treated negative controls (0% hemolysis) was calculated.

### Murine model of GBS infection

Twenty-one days after initial vaccination, analog-vaccinated (*n* = 24) and adjuvant-only mice (*n* = 24) were injected (IP) with~1 × 10^8^ CFU of GBS strain NCTC10/84 suspended in sterile PBS (100 μL injected). At 24 h, all mice were euthanized. Blood was collected via cardiac puncture into heparin tubes (BD). Organs (brain, lung, and spleen) were collected in 1 mL sterile PBS and homogenized. GBS CFU in the blood and each organ was determined by dilution plating on tryptic soy agar (Difco Laboratories). All uninfected mice were housed in specific pathogen-free barrier facilities in the SCRI vivarium, and all GBS-infected mice were housed in ABSL-2 facilities in the SCRI vivarium, as per the Guide for the Care and Use of Laboratory Animals of the National Institutes of Health (8th Edition).

### Statistical analysis

A value of *p* < 0.05 was considered significant. Non-significant *p* values are denoted as “n.s.” in the figures. For in vitro assays, an unpaired, two-tailed Student’s *t* test or one-way analysis of variance (ANOVA) with Tukey’s post test was employed as appropriate to analyze differences between treatment groups, unless otherwise noted. All in vitro experiments were performed three times (unless otherwise noted) in technical triplicate. The correlation between IgG signal intensity and granadaene inhibition was analyzed using a Pearson’s correlation test. Differences in CFU in blood and peripheral organs of vaccinated and unvaccinated mice were determined using Mann–Whitney test, as Gaussian distribution was not assumed in these datasets. GraphPad Prism (version 7.03) was used to compute all statistical tests.

### Reporting summary

Further information on research design is available in the Nature Research Reporting Summary linked to this article.

## Supplementary information

Supplementary Information

Reporting Summary

## Data Availability

The source data underlying Fig. 1b, c, d, e, Fig. 3a, d, Fig. 4a, b, c, and Fig. 5b, c, d, and Supplementary Fig. 3b, c and Supplementary Fig. 4 are provided as a Source Data file. All other relevant data supporting the key findings of this study are available within the article and its Supplementary Information file or from the corresponding authors upon request.

## Source Data

Source Data

## References

[1] George KM, Pascopella L, Welty DM, Small PL (2000). A *Mycobacterium ulcerans* toxin, mycolactone, causes apoptosis in guinea pig ulcers and tissue culture cells. Infect. Immun..

[2] Nitenberg M (2018). The potent effect of mycolactone on lipid membranes. PLoS Pathog..

[3] Zulianello L (2006). Rhamnolipids are virulence factors that promote early infiltration of primary human airway epithelia by Pseudomonas aeruginosa. Infect. Immun..

[4] McClure CD, Schiller NL (1992). Effects of *Pseudomonas aeruginosa* rhamnolipids on human monocyte-derived macrophages. J. Leukoc. Biol..

[5] Lynch A (2017). The Bacteroidales produce an *N*-acylated derivative of glycine with both cholesterol-solubilising and hemolytic activity. Sci. Rep..

[6] Whidbey C (2013). A hemolytic pigment of Group B *Streptococcus* allows bacterial penetration of human placenta. J. Exp. Med..

[7] Whidbey C (2015). A streptococcal lipid toxin induces membrane permeabilization and pyroptosis leading to fetal injury. EMBO Mol. Med..

[8] Bianchi-Jassir F (2017). Preterm birth associated with Group B *Streptococcus* maternal colonization worldwide: systematic review and meta-analyses. Clin. Infect. Dis..

[9] Schrag SJ (2000). Group B streptococcal disease in the era of intrapartum antibiotic prophylaxis. N. Engl. J. Med..

[10] Seale AC (2017). Estimates of the burden of Group B Streptococcal disease worldwide for pregnant women, stillbirths, and children. Clin. Infect. Dis..

[11] Leroux-Roels, G. et al. Safety and immunogenicity of a second dose of an investigational maternal trivalent Group B streptococcus vaccine in non-pregnant women 4–6 years after a first dose: results from a phase 2 trial. *Clin. Infect. Dis.*10.1093/cid/ciz737 (2019). 10.1093/cid/ciz737 PMC7286364 31394574

[12] Edwards, M. S. & Baker, C. J. Group B. Streptococcal disease: interim prevention at 50 years and counting. *Clin. Infect. Dis.*10.1093/cid/ciz738 (2019). 10.1093/cid/ciz738 31394571

[13] Skoff TH (2009). Increasing burden of invasive group B streptococcal disease in nonpregnant adults, 1990–2007. Clin. Infect. Dis..

[14] Ballard MS (2016). The changing epidemiology of group B streptococcus bloodstream infection: a multi-national population-based assessment. Infect. Dis. (Lond.).

[15] Sendi P, Johansson L, Norrby-Teglund A (2008). Invasive group B Streptococcal disease in non-pregnant adults: a review with emphasis on skin and soft-tissue infections. Infection.

[16] Khan MA, Faiz A, Ashshi AM (2015). Maternal colonization of group B streptococcus: prevalence, associated factors and antimicrobial resistance. Ann. Saudi Med..

[17] Castor ML (2008). Antibiotic resistance patterns in invasive group B streptococcal isolates. Infect. Dis. Obstet. Gynecol..

[18] Rosa-Fraile M, Rodriguez-Granger J, Haidour-Benamin A, Cuerva JM, Sampedro A (2006). Granadaene: proposed structure of the group B Streptococcus polyenic pigment. Appl. Environ. Microbiol..

[19] Lupo A, Ruppen C, Hemphill A, Spellerberg B, Sendi P (2014). Phenotypic and molecular characterization of hyperpigmented group B Streptococci. Int. J. Med. Microbiol..

[20] Whidbey, C. et al. A Hyperhemolytic/hyperpigmented group B Streptococcus strain with a CovR mutation isolated from an adolescent patient with sore throat. *Clin. Res. Infect. Dis.* **2**, 3 (2015). PMC4762654 26913295

[21] Sendi P (2009). Bacterial phenotype variants in group B streptococcal toxic shock syndrome. Emerg. Infect. Dis..

[22] Siemens, N. et al. Prothrombotic and proinflammatory activities of the beta-hemolytic group B Streptococcal pigment. *J. Innate Immun.*10.1159/000504002, 1–13 (2019). 10.1159/000504002 PMC7383282 31743913

[23] Randis TM (2014). Group B Streptococcus beta-hemolysin/cytolysin breaches maternal–fetal barriers to cause preterm birth and intrauterine fetal demise in vivo. J. Infect. Dis..

[24] Boldenow, E. et al. Group B Streptococcus circumvents neutrophils and neutrophil extracellular traps during amniotic cavity invasion and preterm labor. *Sci. Immunol.* **1**, 10.1126/sciimmunol.aah4576 (2016). 10.1126/sciimmunol.aah4576 PMC5089172 27819066

[25] Doran KS, Liu GY, Nizet V (2003). Group B streptococcal beta-hemolysin/cytolysin activates neutrophil signaling pathways in brain endothelium and contributes to development of meningitis. J. Clin. Invest..

[26] Gendrin C (2015). Mast cell degranulation by a hemolytic lipid toxin decreases GBS colonization and infection. Sci. Adv..

[27] Madden KS, Mosa FA, Whiting A (2014). Non-isoprenoid polyene natural products–structures and synthetic strategies. Org. Biomol. Chem..

[28] Paradas M (2012). Clarifying the structure of granadaene: total synthesis of related analogue [2]-granadaene and confirmation of its absolute stereochemistry. Bioorg. Med. Chem..

[29] Clarke D (2016). Group B Streptococcus induces a robust IFN-gamma response by CD4(+) T cells in an in vitro and in vivo model. J. Immunol. Res..

[30] Patras KA, Rosler B, Thoman ML, Doran KS (2015). Characterization of host immunity during persistent vaginal colonization by Group B Streptococcus. Mucosal Immunol..

[31] Pritzlaff CA (2001). Genetic basis for the beta-haemolytic/cytolytic activity of group B Streptococcus. Mol. Microbiol..

[32] Pietkiewicz S, Schmidt JH, Lavrik IN (2015). Quantification of apoptosis and necroptosis at the single cell level by a combination of imaging flow cytometry with classical annexin V/propidium iodide staining. J. Immunol. Methods.

[33] Cibrian D, Sanchez-Madrid F (2017). CD69: from activation marker to metabolic gatekeeper. Eur. J. Immunol..

[34] Lembo A (2010). Regulation of CovR expression in Group B Streptococcus impacts blood–brain barrier penetration. Mol. Microbiol..

[35] Andersen OS, Koeppe RE (2007). Bilayer thickness and membrane protein function: an energetic perspective. Annu. Rev. Biophys. Biomol. Struct..

[36] Mitra K, Ubarretxena-Belandia I, Taguchi T, Warren G, Engelman DM (2004). Modulation of the bilayer thickness of exocytic pathway membranes by membrane proteins rather than cholesterol. Proc. Natl. Acad. Sci. USA.

[37] Nagle JF, Tristram-Nagle S (2000). Structure of lipid bilayers. Biochim. Biophys. Acta.

[38] Horobin RW (2014). Where do dyes go inside living cells? Predicting uptake, intracellular localisation, and accumulation using QSAR models. Color Technol..

[39] Gras S, Van Rhijn I, Shahine A, Le Nours J (2018). Molecular recognition of microbial lipid-based antigens by T cells. Cell Mol. Life Sci..

[40] Doran KS, Chang JC, Benoit VM, Eckmann L, Nizet V (2002). Group B streptococcal beta-hemolysin/cytolysin promotes invasion of human lung epithelial cells and the release of interleukin-8. J. Infect. Dis..

[41] Wilkinson HW (1977). Nontypable group B streptococci isolated from human sources. J. Clin. Microbiol..

[42] Armistead B (2019). The cyl genes reveal the biosynthetic and evolutionary origins of the group B Streptococcus hemolytic lipid, granadaene. Front. Microbiol..

[43] Fortunati T, D'Acunto M, Caruso T, Spinella A (2015). Chemoenzymatic preparation of musky macrolactones. Tetrahedron.

[44] Sivasubramanian K, Kaanumalle LS, Uppili S, Ramamurthy V (2007). Value of zeolites in asymmetric induction during photocyclization of pyridones, cyclohexadienones and naphthalenones. Org. Biomol. Chem..

[45] Wang G, Huang Z, Negishi E (2009). Highly stereoselective and efficient synthesis of ω-heterofunctional di- and trienoic esters for Horner–Wadsworth–Emmons reaction via alkyne hydrozirconation and Pd-catalyzed alkenylation. Tetrahedron Lett..

[46] Dumont FJ, Staruch MJ, Fischer P, DaSilva C, Camacho R (1998). Inhibition of T cell activation by pharmacologic disruption of the MEK1/ERK MAP kinase or calcineurin signaling pathways results in differential modulation of cytokine production. J. Immunol..

[47] Bjorndahl JM, Sung SS, Hansen JA, Fu SM (1989). Human T cell activation: differential response to anti-CD28 as compared to anti-CD3 monoclonal antibodies. Eur. J. Immunol..

[48] Van Belle K (2016). Comparative in vitro immune stimulation analysis of primary human B cells and B cell lines. J. Immunol. Res..

