# Supplementary material for: Lipid analogs reveal features critical for hemolysis and diminish granadaene mediated Group B Streptococcus infection
Source: Nat Commun. 2020 Mar 20;11:1502. doi: 10.1038/s41467-020-15282-0 (PMC7083881; doi:10.1038/s41467-020-15282-0)
Supplement: Supplementary file 1 — Supplementary Information [file 41467_2020_15282_MOESM1_ESM.pdf]

Lipid analogs reveal features critical for hemolysis and diminish granadaene mediated Group B Streptococcus infection

Armistead, et al.

Supplementary Information

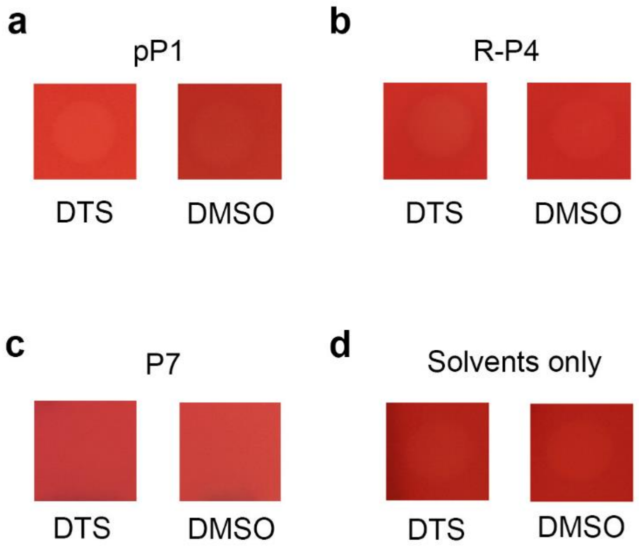

Supplementary Figure 1. Synthetic compounds pP1, R-P4, P7, and the solvents DMSO and DTS are not hemolytic. (**a-c**) Each compound was resuspended in either DTS or DMSO at 0.02 M, and 5 µL was spotted onto red blood agar and incubated at 37 °C overnight. (**d**) 5 µL of the solvent (DTS or DMSO) was spotted onto red blood agar and incubated at 37 °C overnight.

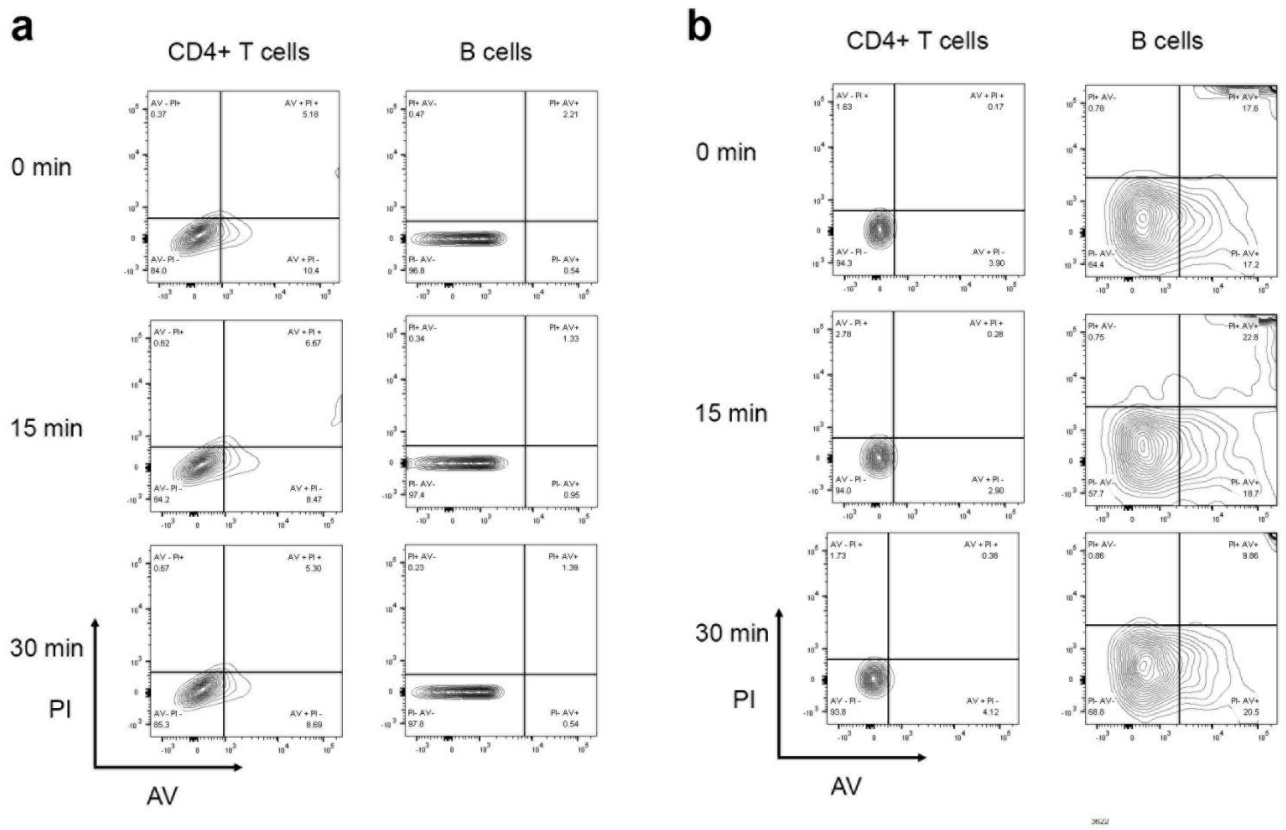

Supplementary Figure 2. Non-hemolytic GBS and its extract do not induce cell lysis. PI uptake and Annexin V staining were measured using flow cytometry in CD4+ T cells and B cells following incubation with (**a**) non-hemolytic GBS at an MOI of 10 or with (**b**) GBSΔ*cylE* extract at the indicated time points.

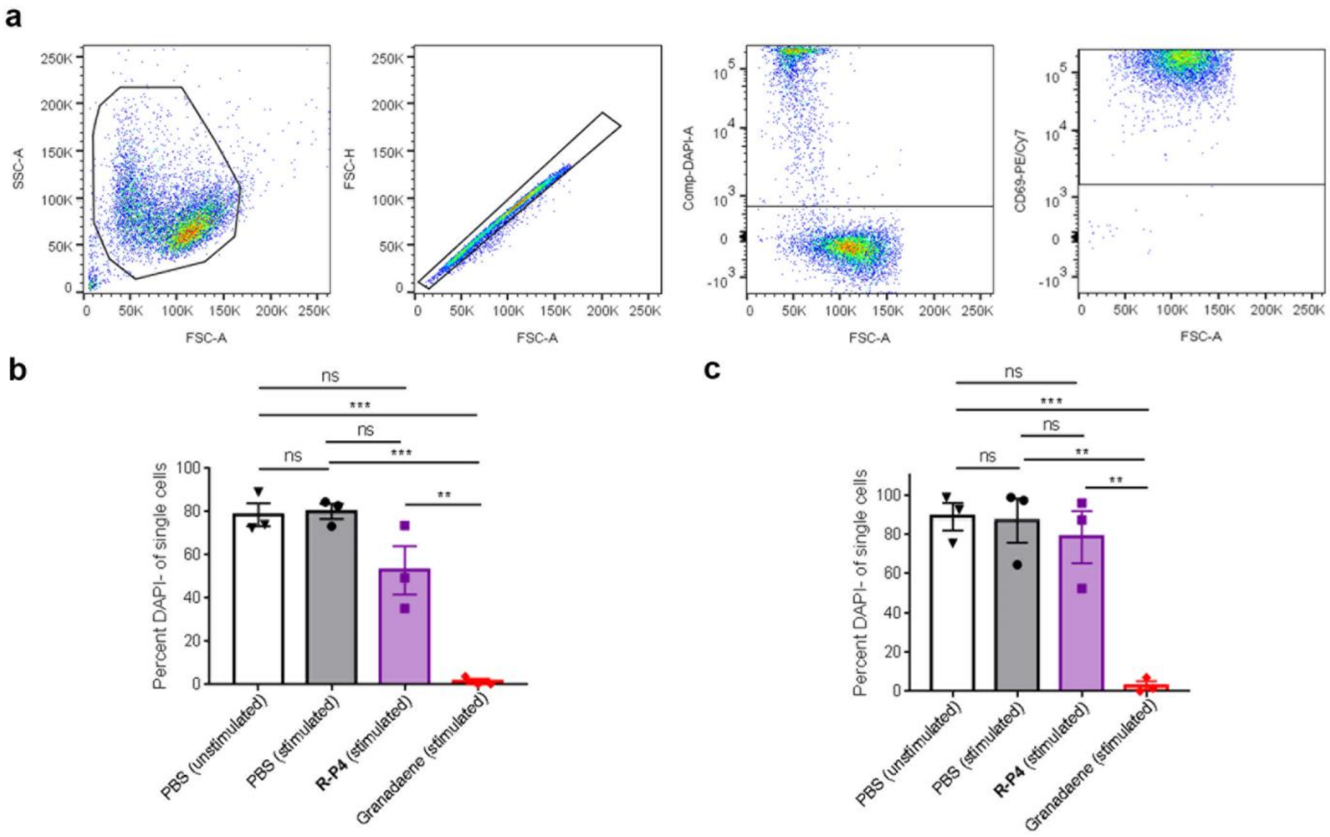

Supplementary Figure 3. Role of R-P4 and granadaene on activation of CD4+ T cells and B cells. (**a**) The sequential gating strategy for CD4+ T cell and B cell activation experiments is shown (left to right) with example data from the PBS (stimulated) group in a CD4+ T cell experiment. Light scatter was used to include events based on size, then singlets. Then, live (DAPI-) events were analyzed for CD69. DAPI and CD69 gates were defined using fluorescence minus one or unstained controls. (**b**) Differences in the percent of DAPI- cells among each treatment group in CD4+ T cell experiments were determined using one-way ANOVA with Tukey's post-test. PBS (unstimulated) vs. PBS (stimulated): $p = 0.9978$, PBS (unstimulated) vs. R-P4 (stimulated): $p = 0.0861$, PBS (unstimulated) vs. granadaene: $p = 0.0001$, PBS (stimulated) vs. R-P4 (stimulated): $p = 0.0669$, PBS (stimulated) vs. granadaene (stimulated): $p = 0.0001$, R-P4 (stimulated) vs. granadaene: $p = 0.0022$. Data are representative of three independent experiments. Mean and SEM are shown. (**c**) Differences in the percent of DAPI- cells among each treatment group in B cell experiments were

determined using one-way ANOVA with Tukey's post-test. PBS (unstimulated) vs. PBS (stimulated): $p = 0.9986$, PBS (unstimulated) vs. R-P4 (stimulated): $p = 0.8612$, PBS (unstimulated) vs. granadaene: $p = 0.0009$, PBS (stimulated) vs. R-P4 (stimulated): $p = 0.9210$, PBS (stimulated) vs. granadaene (stimulated): $p = 0.0011$, R-P4 (stimulated) vs. granadaene: $p = 0.0021$. Source data for panels b and c are provided in the Source data file. Data are representative of three independent experiments. Mean and SEM are shown.

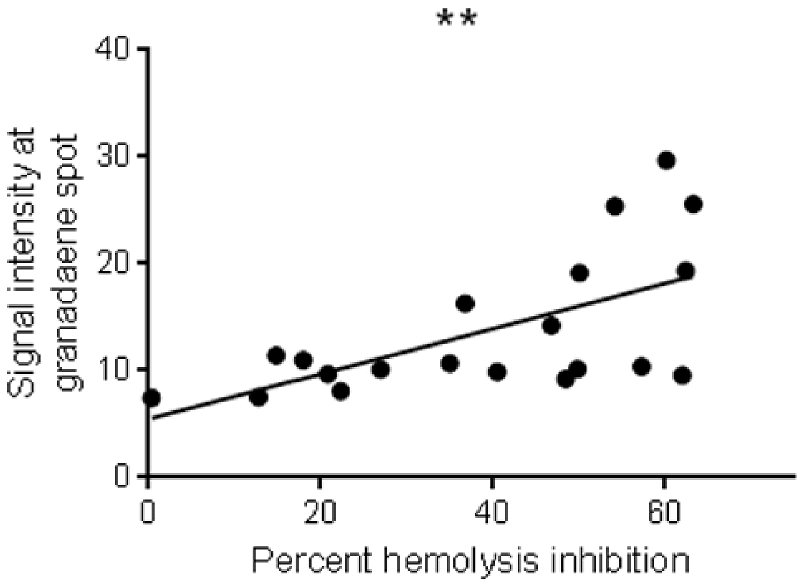

Supplementary Figure 4. The amount of granadaene-bound IgG in mouse plasma is correlated with inhibition of granadaene activity *ex vivo*. Immunoblots and inhibition of granadaene hemolysis using plasma samples from analog-vaccinated and adjuvant-only mice are shown in Fig 5b and c. A correlation analysis on these data was performed using the Pearson's correlation test ($p = 0.0040$). Source data are provided in the Source data file.

Supplementary Figure 5. ^{1}H and ^{13}C NMR spectra of synthetic lipids and precursors

^{1}H NMR **3** (400 MHz, $CDCl_3$)

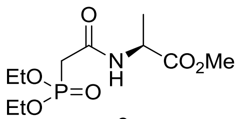

3

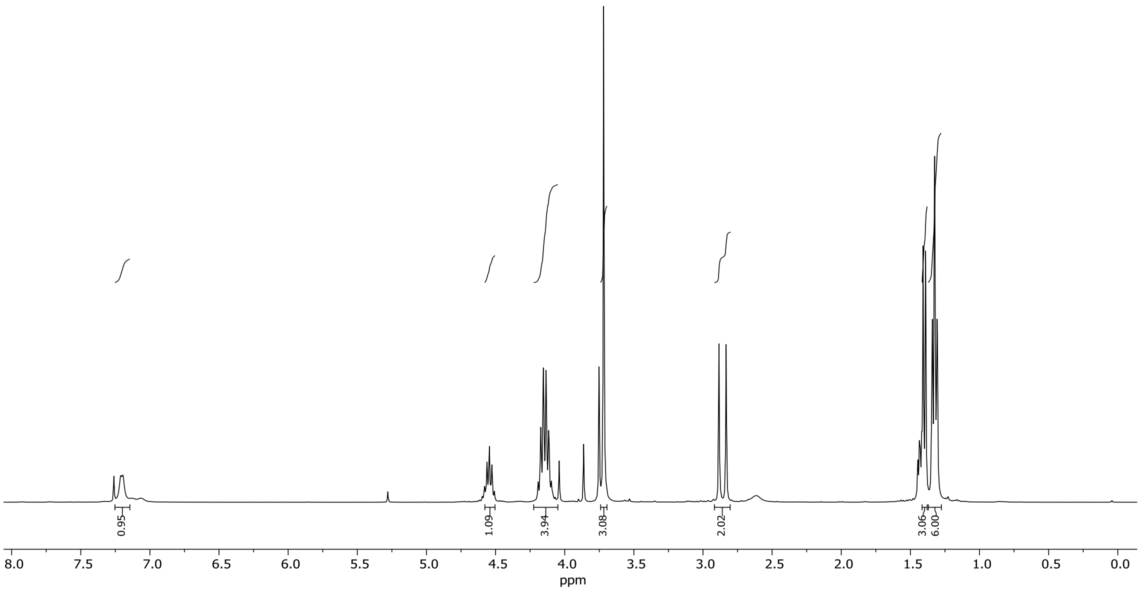

^{13}C NMR **3** (101 MHz, $CDCl_3$)

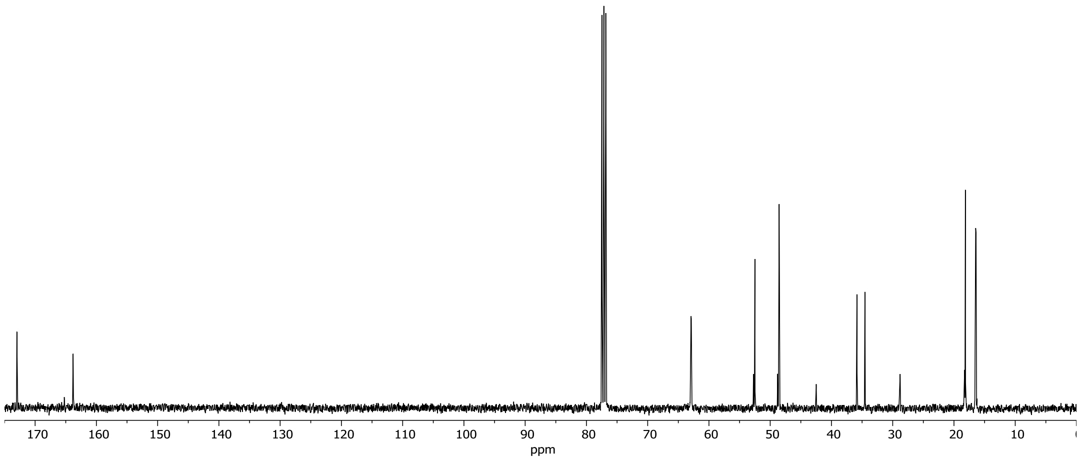

^{31}P NMR **3** (202 MHz, $CDCl_3$)

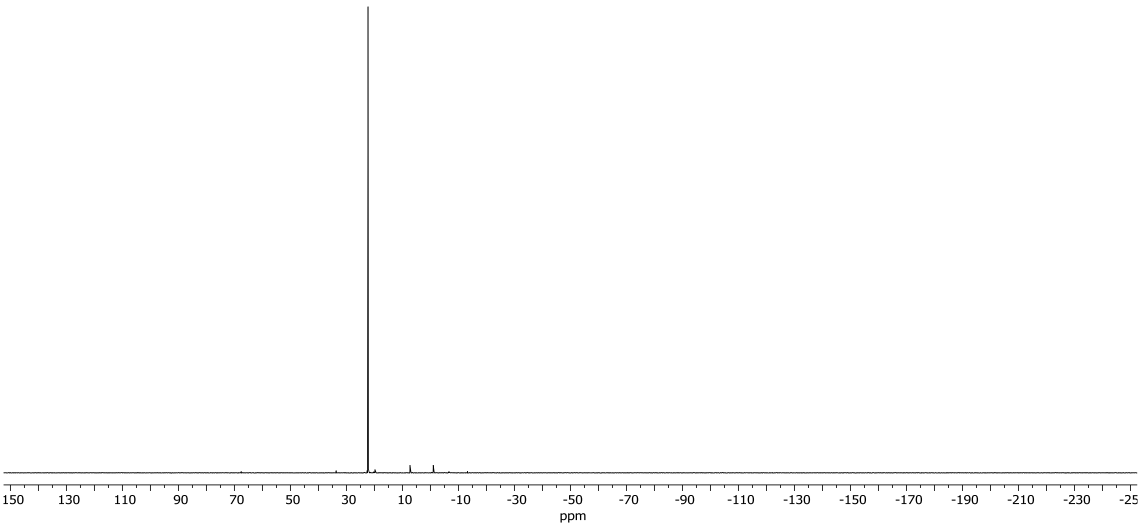

^{1}H NMR **7** (500 MHz, $CDCl_3$)

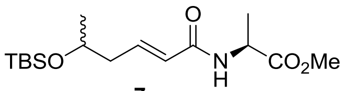

7

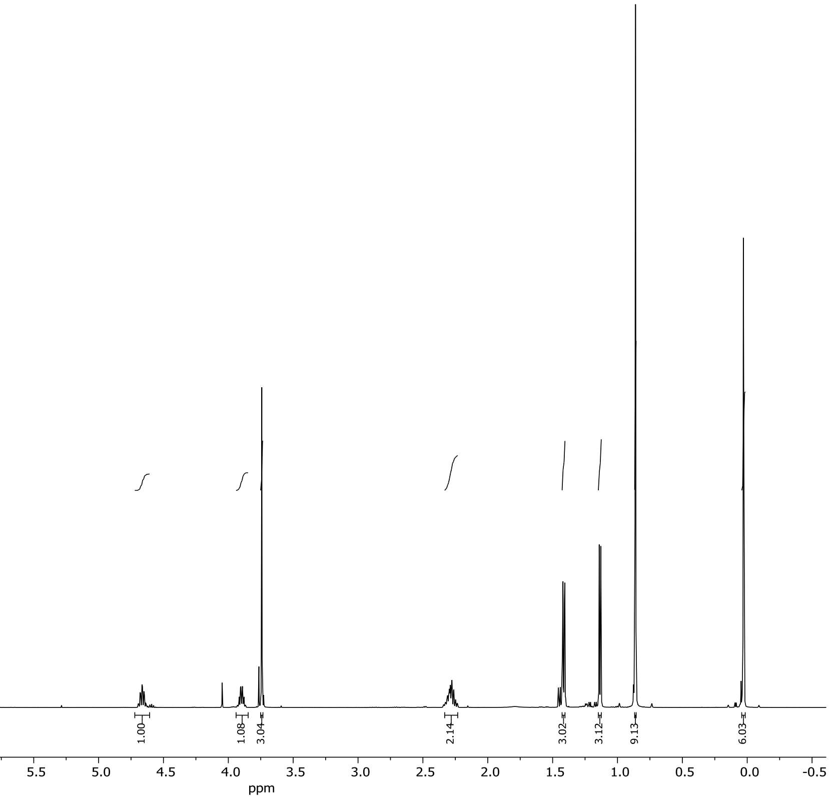

^{13}C NMR **7** (126 MHz, $CDCl_3$)

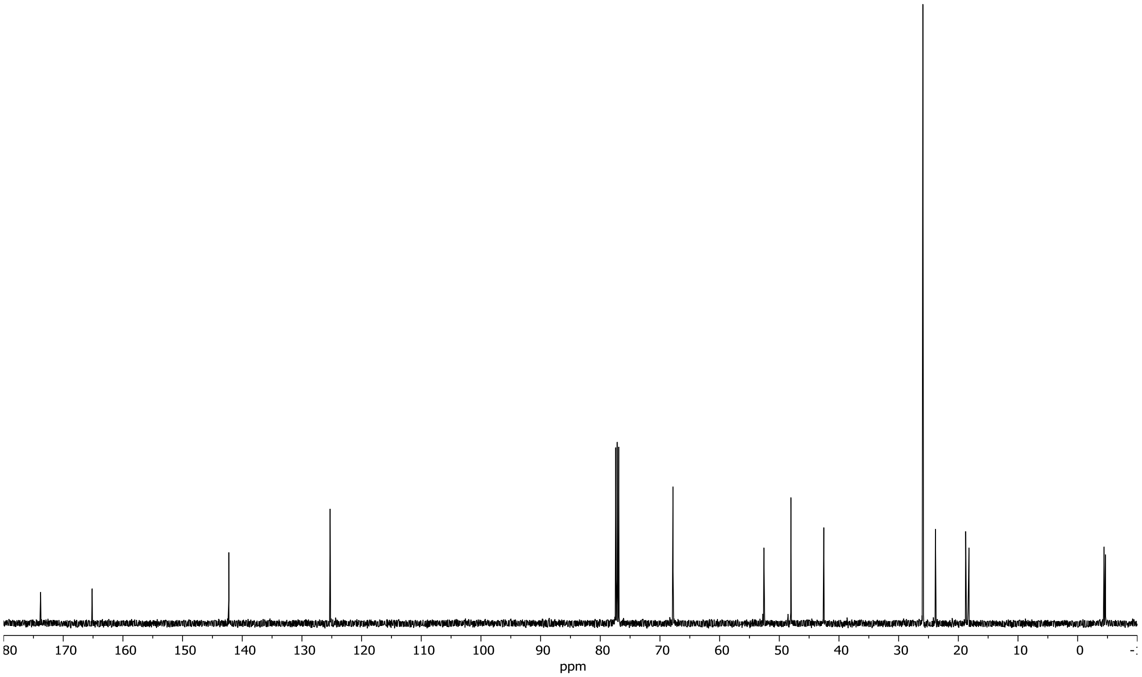

^{1}H NMR **pP1** (500 MHz, $CDCl_3$)

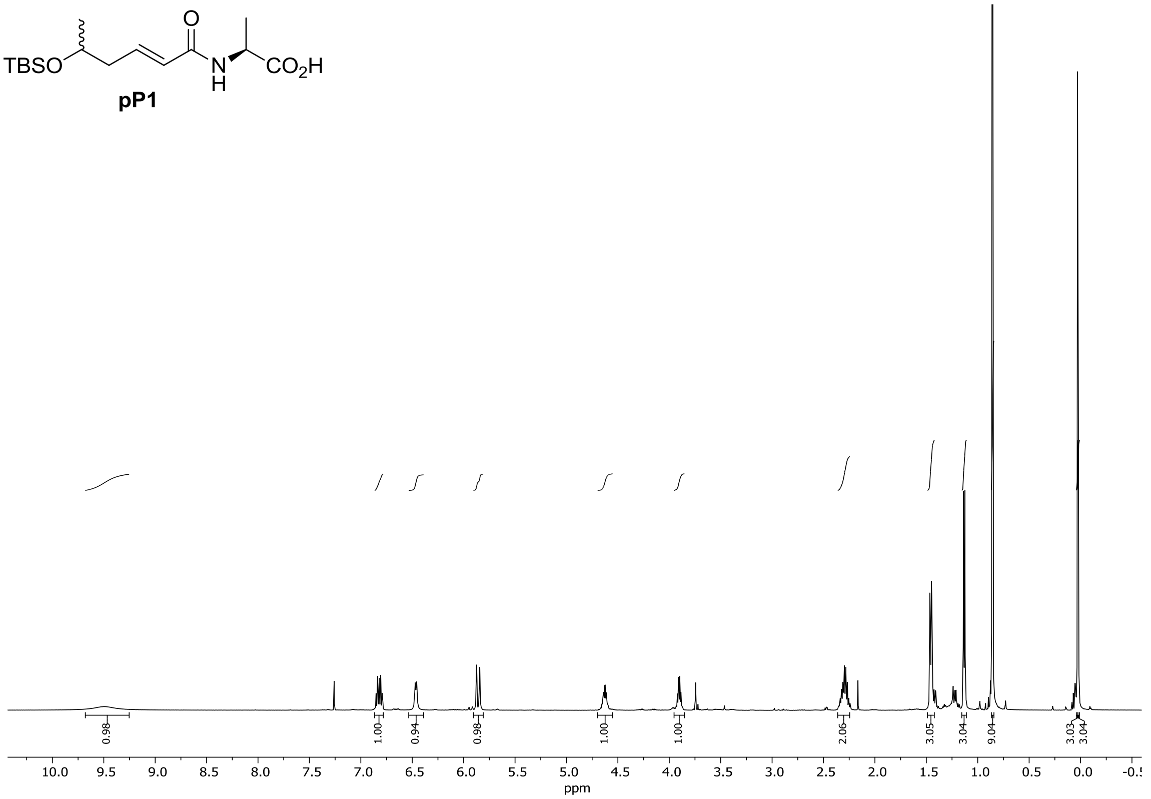

^{13}C NMR **pP1** (126 MHz, $CDCl_3$)

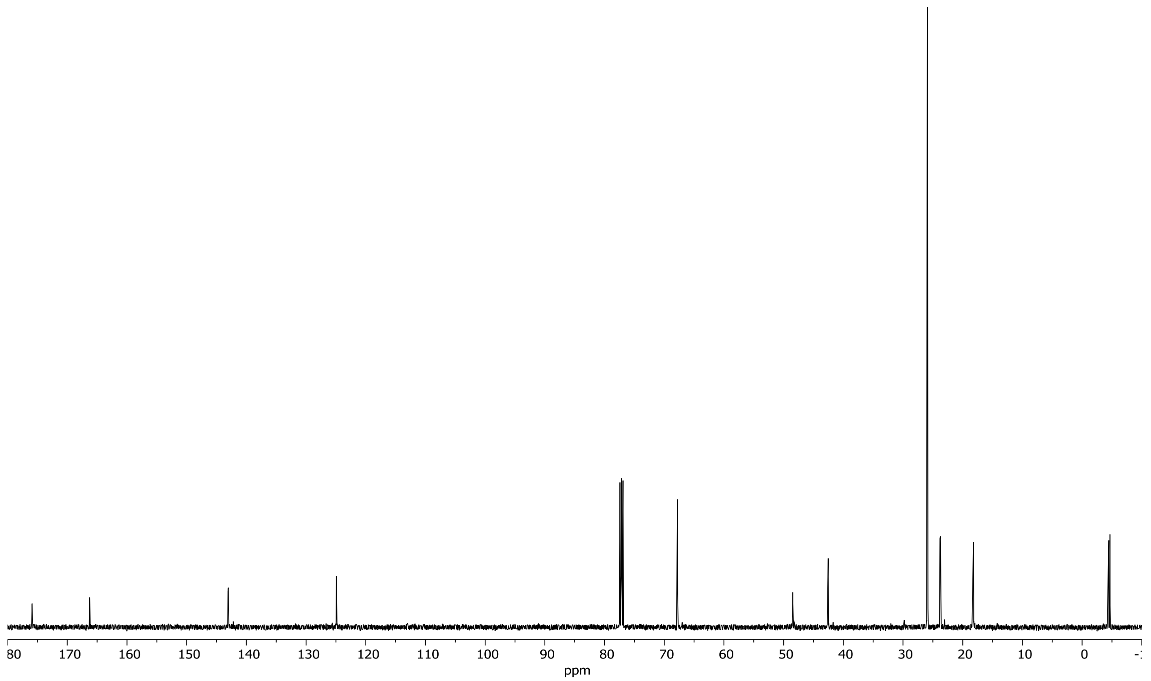

^{1}H NMR **9** major isomer (*E*,*E*,*E*-isomer) (500 MHz, $CDCl_3$)

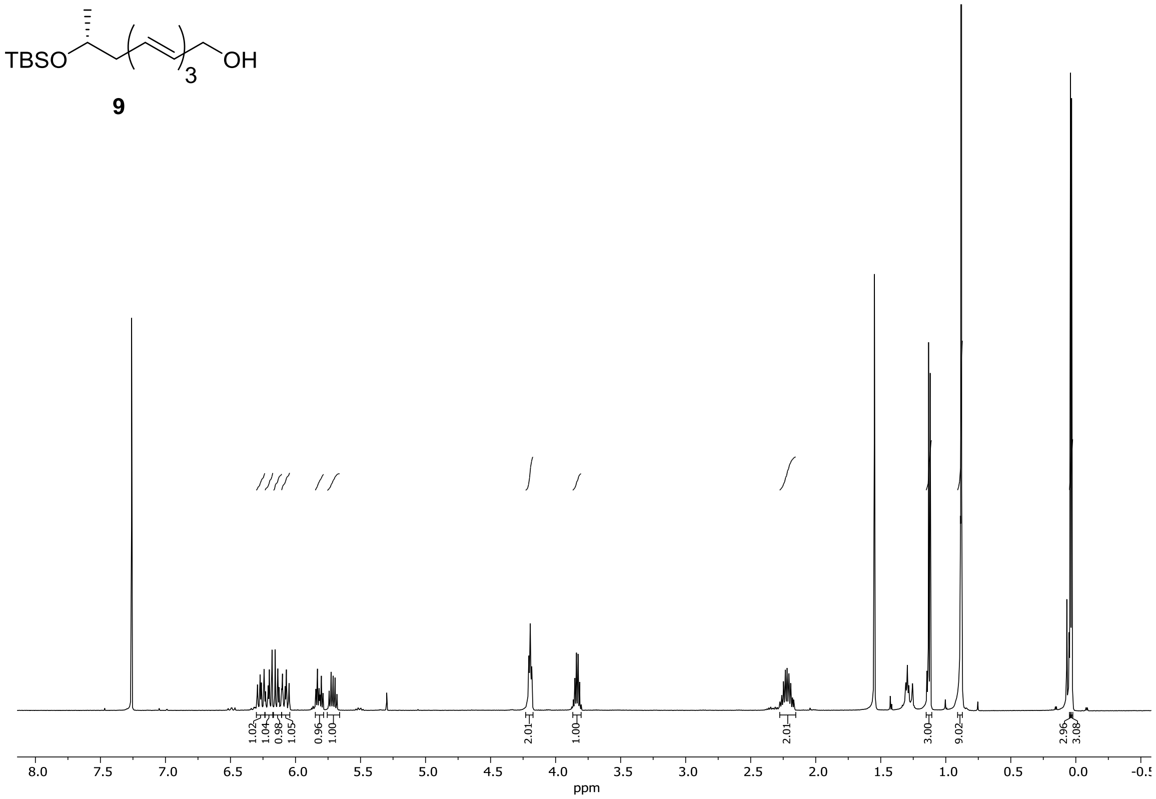

^{13}C NMR **9** major isomer (*E*,*E*,*E*-isomer) (126 MHz, $CDCl_3$)

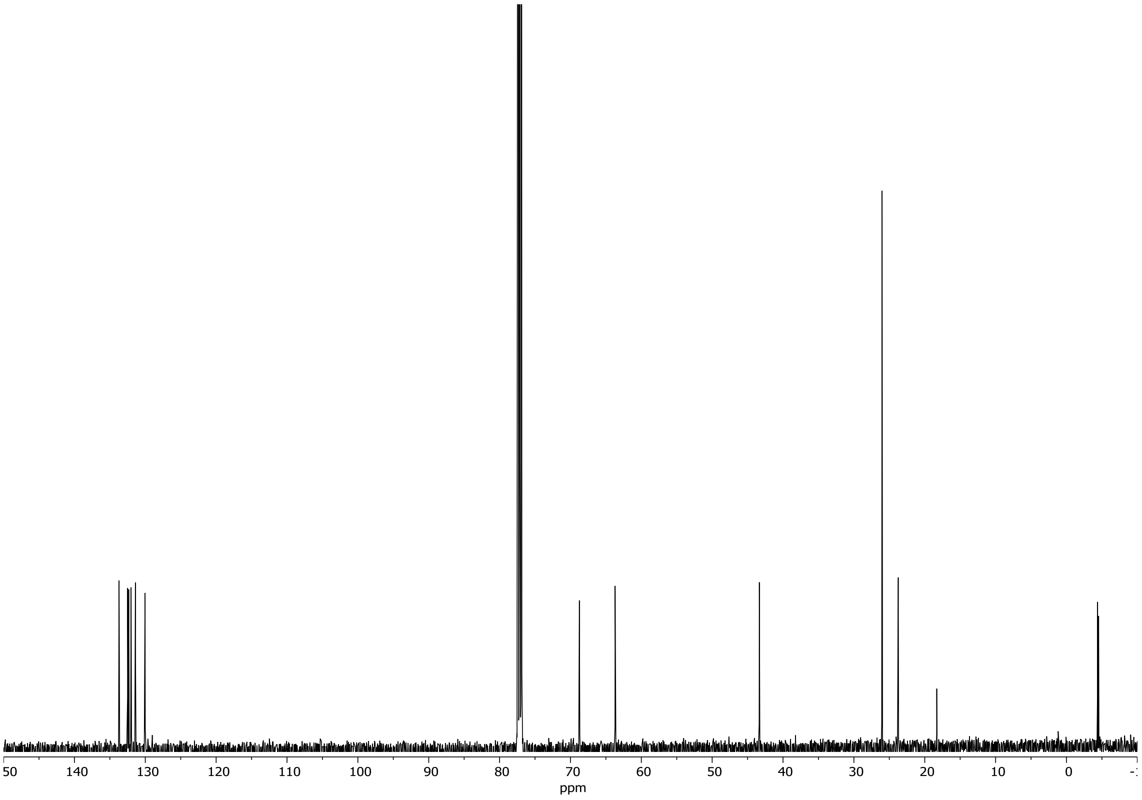

^{1}H NMR **9** minor isomer (*E*,*Z*,*E*-isomer) (500 MHz, $CDCl_3$)

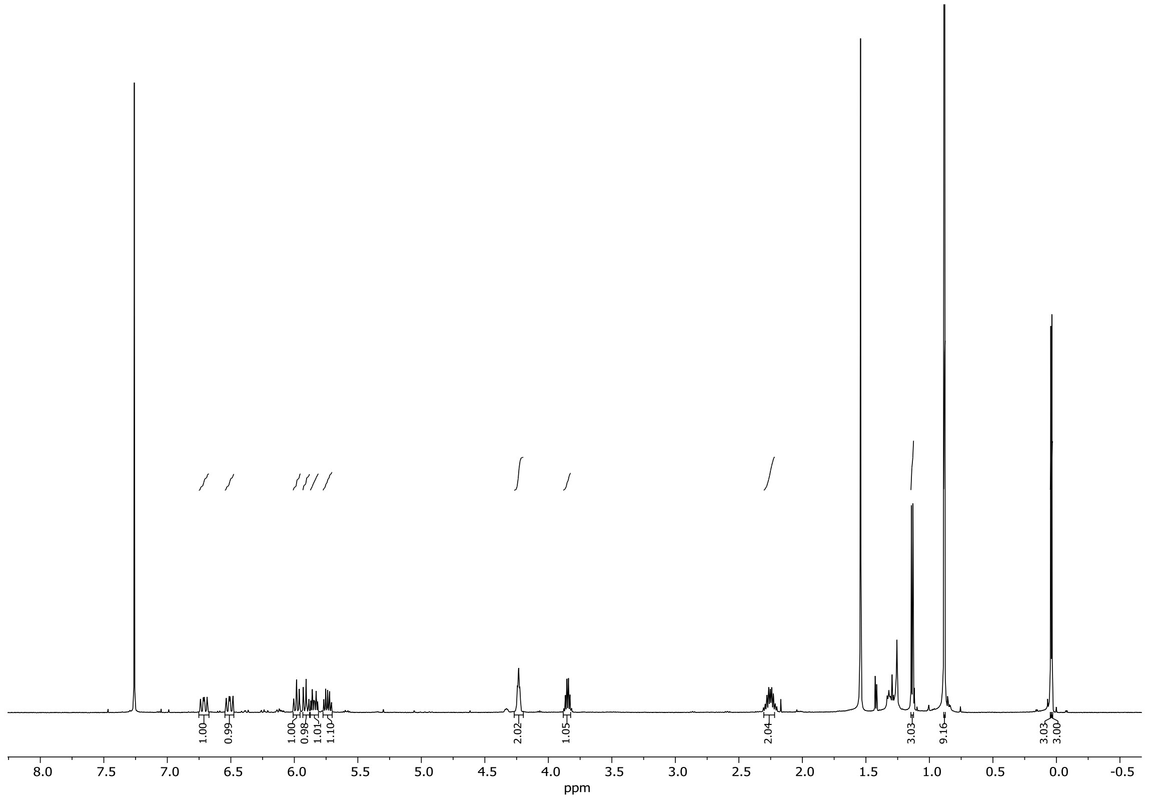

HSQC

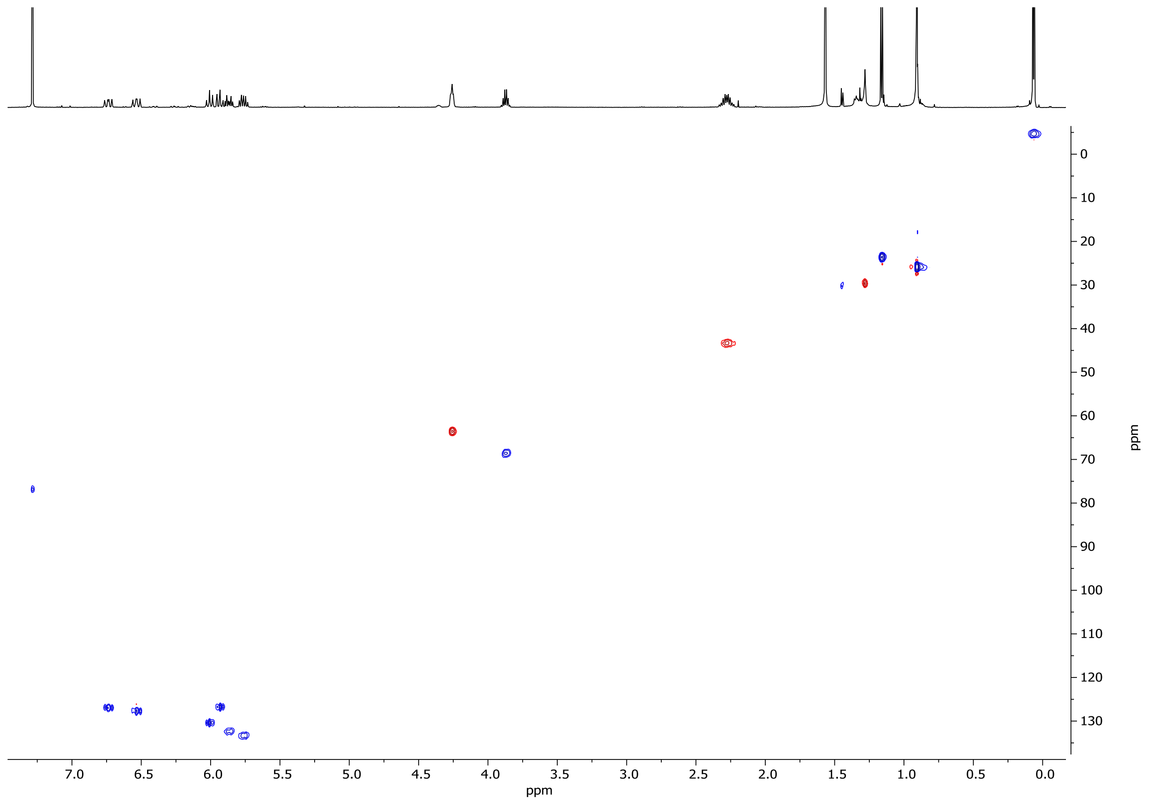

^{1}H NMR **9** minor isomer (*Z*,*E*,*E*-isomer) (500 MHz, $CDCl_3$)

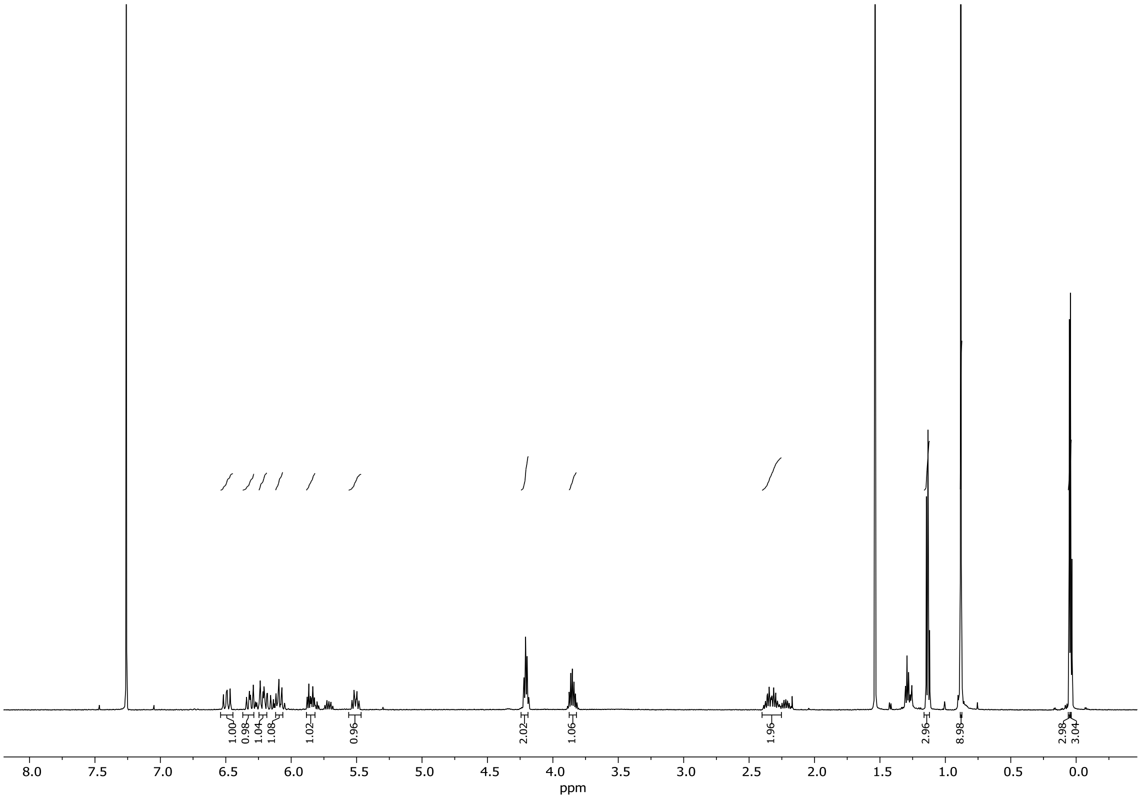

HSQC

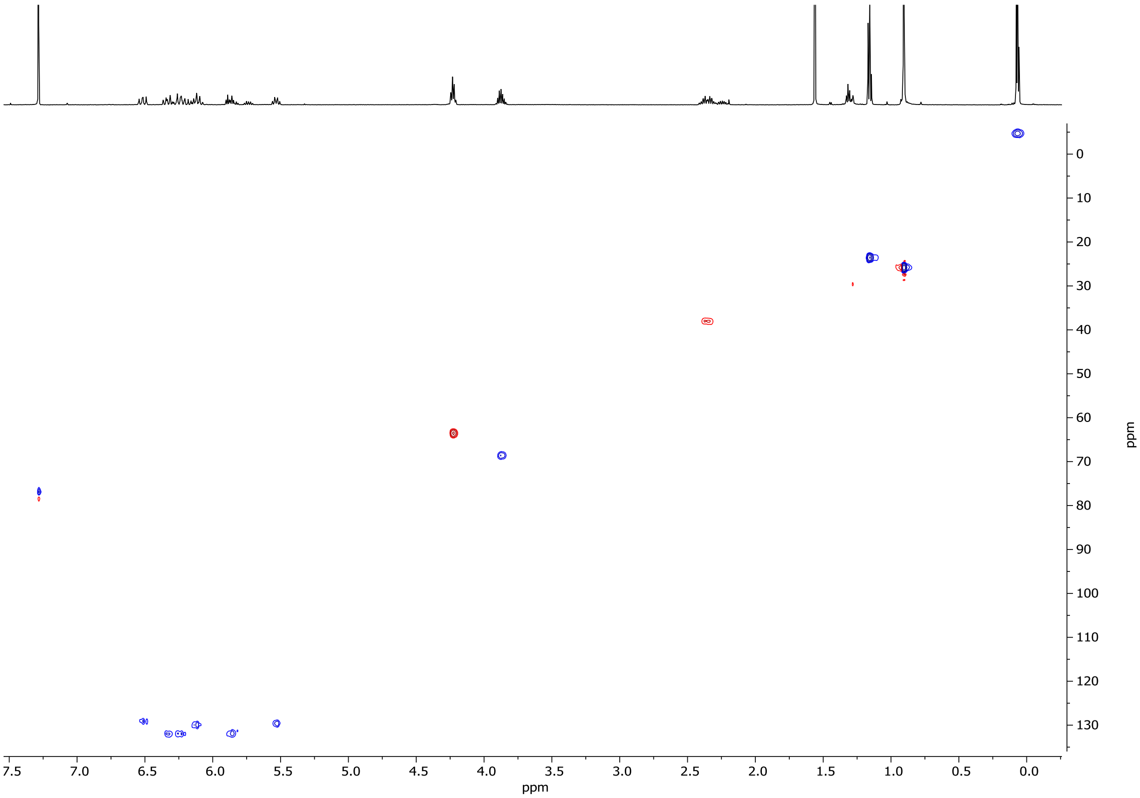

^{1}H NMR **10** *E*,*E*,*E*-isomer (400 MHz, $CDCl_3$)

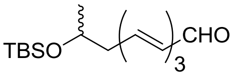

10

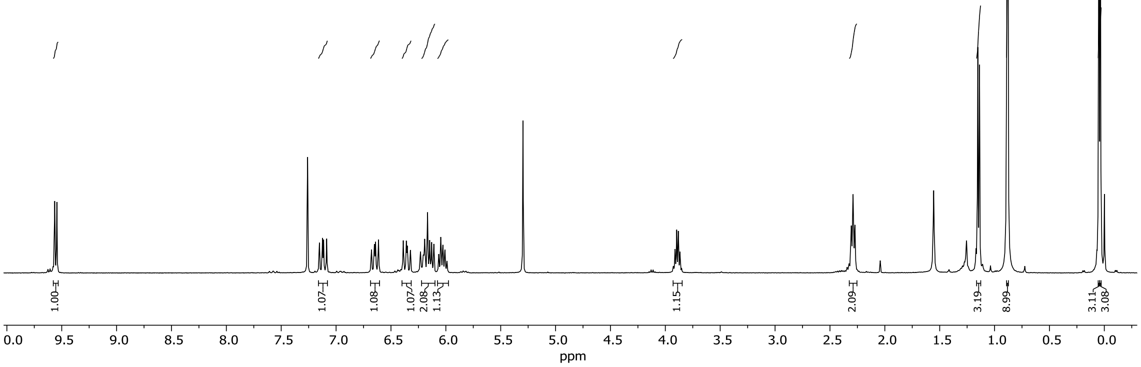

^{13}C NMR **10** *E*,*E*,*E*-isomer (101 MHz, $CDCl_3$)

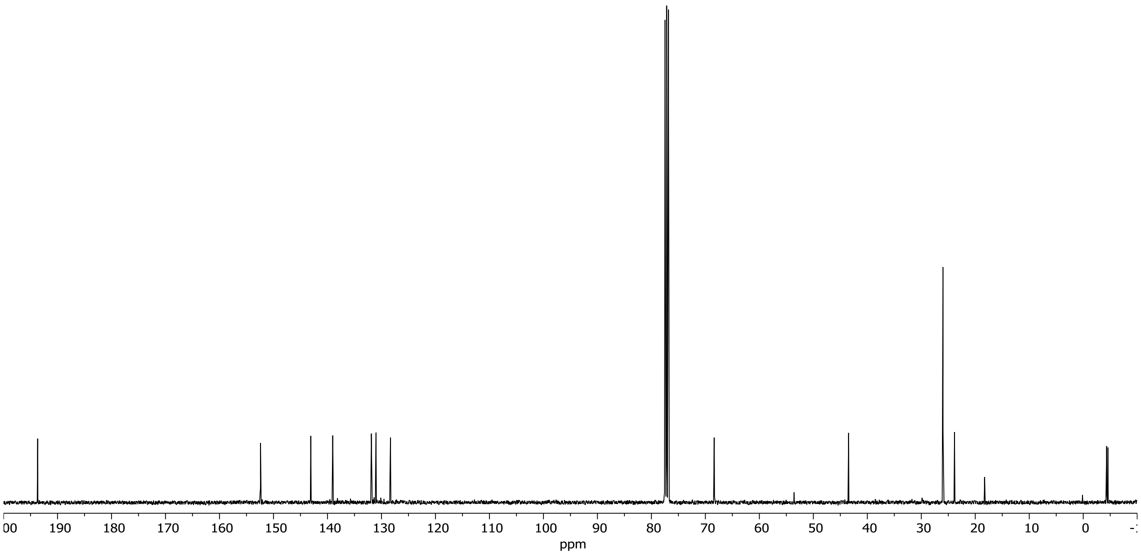

^{1}H NMR **11** *E*,*E*,*E*,*E*-isomer (500 MHz, $CDCl_3$)

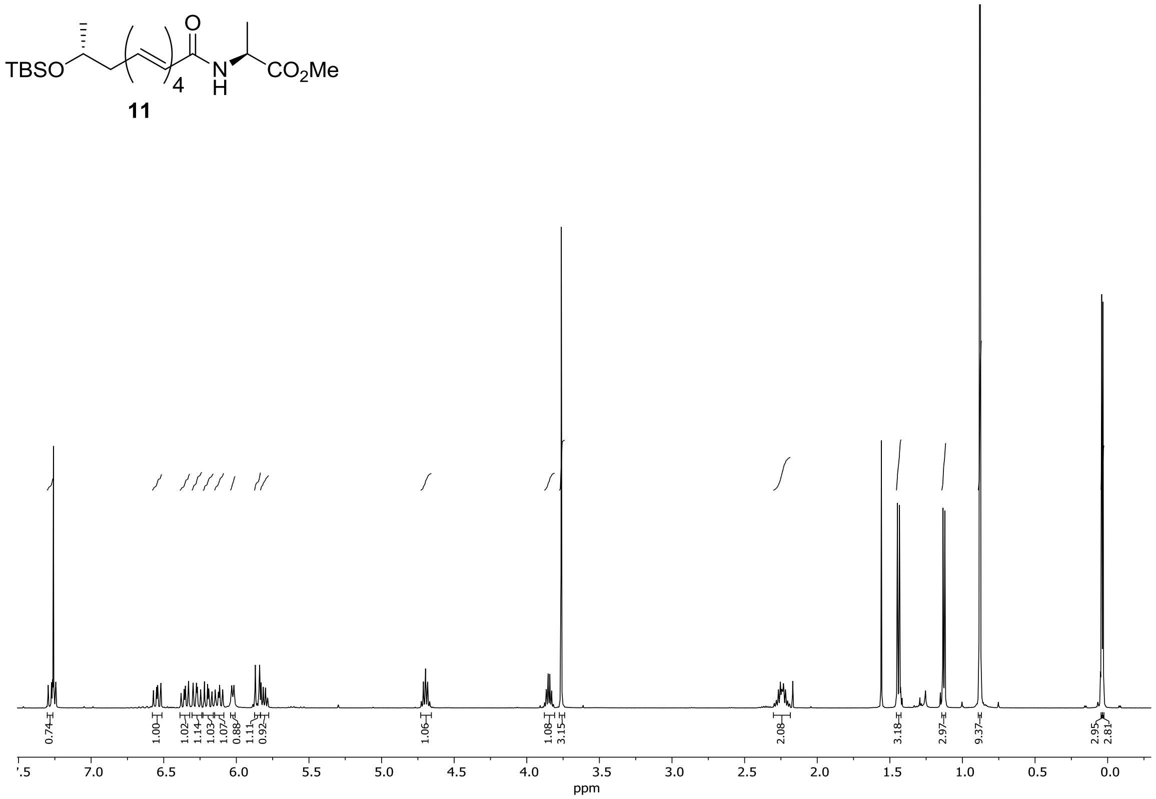

^{13}C NMR **11** *E*,*E*,*E*,*E*-isomer (126 MHz, $CDCl_3$)

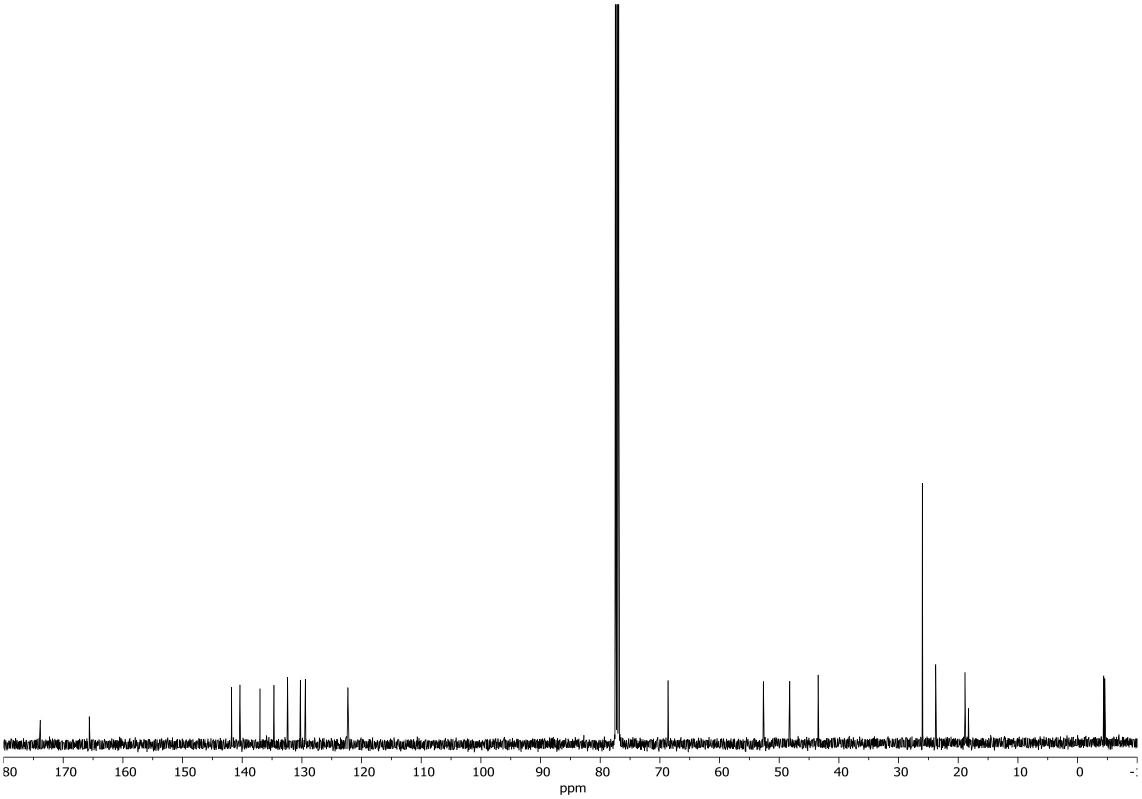

^{1}H NMR **12** *E*,*E*,*E*,*E*-isomer (500 MHz, $CDCl_3$)

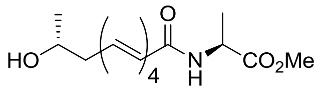

12

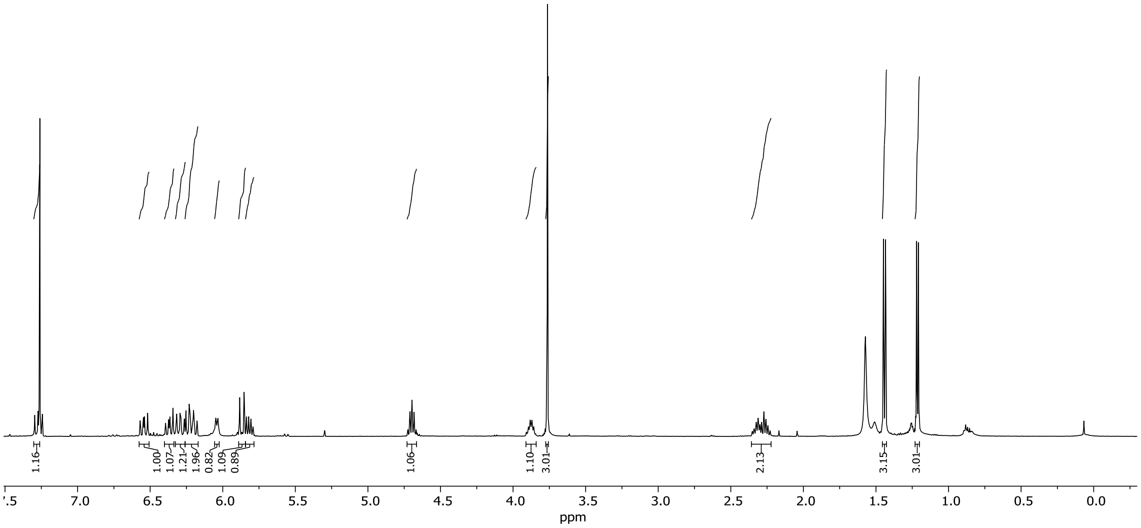

^{13}C NMR **12** *E*,*E*,*E*,*E*-isomer (126 MHz, $CDCl_3$)

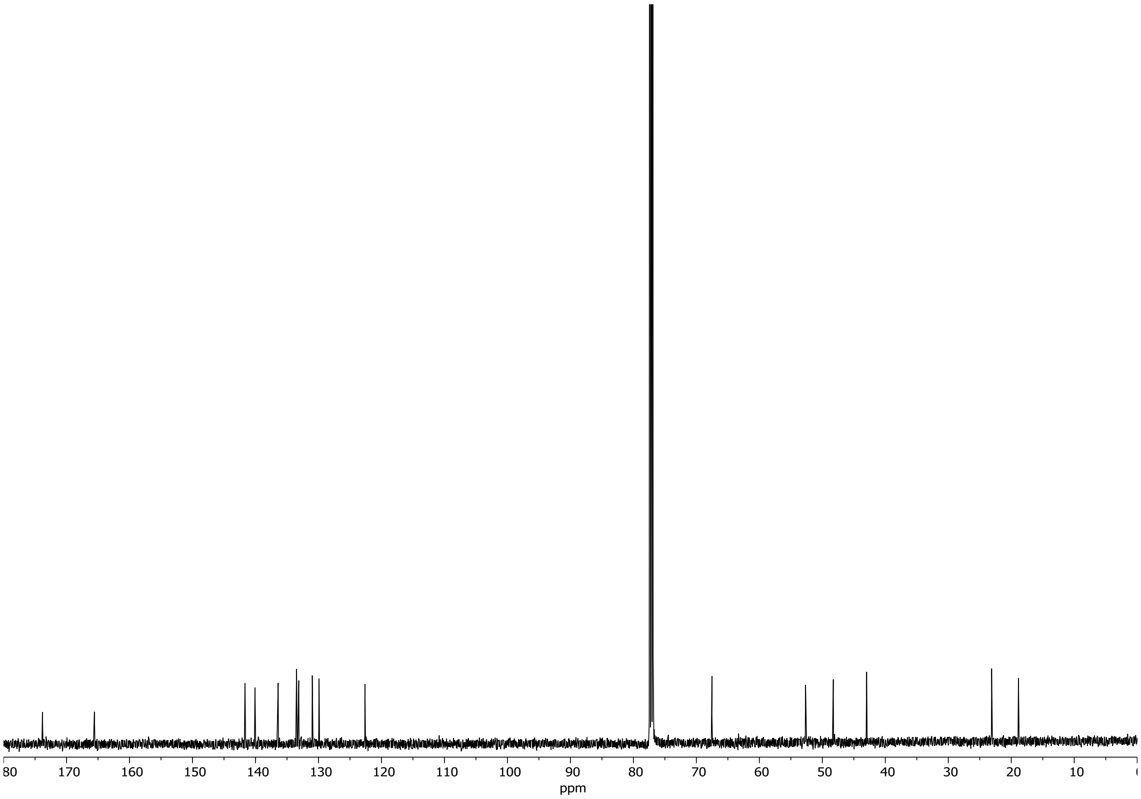

^{1}H NMR **14** *E*,*E*,*E*,*E*-isomer (500 MHz, $CDCl_3$)

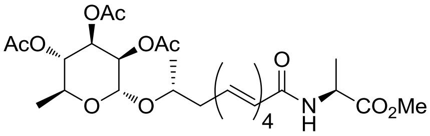

14

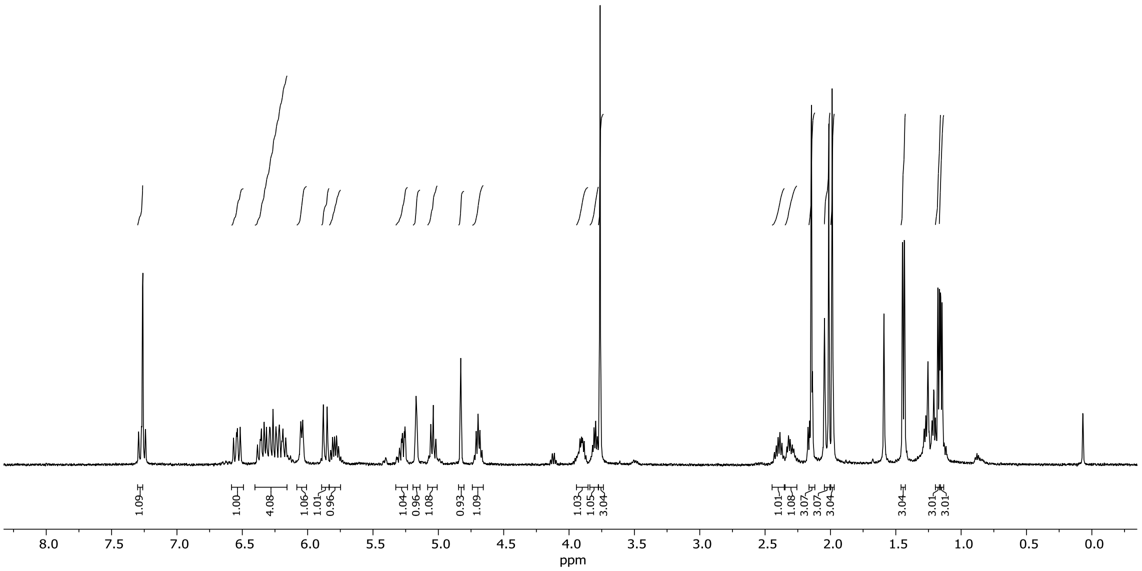

^{13}C NMR **14** *E*,*E*,*E*,*E*-isomer (126 MHz, $CDCl_3$)

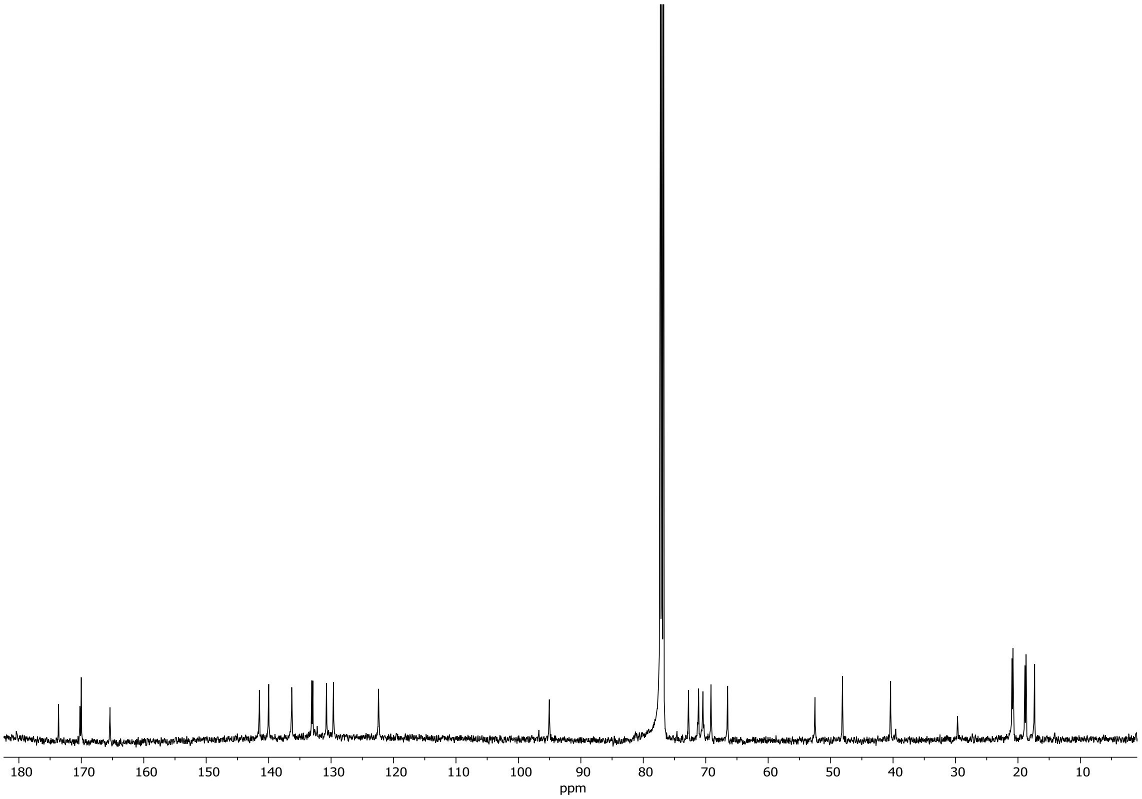

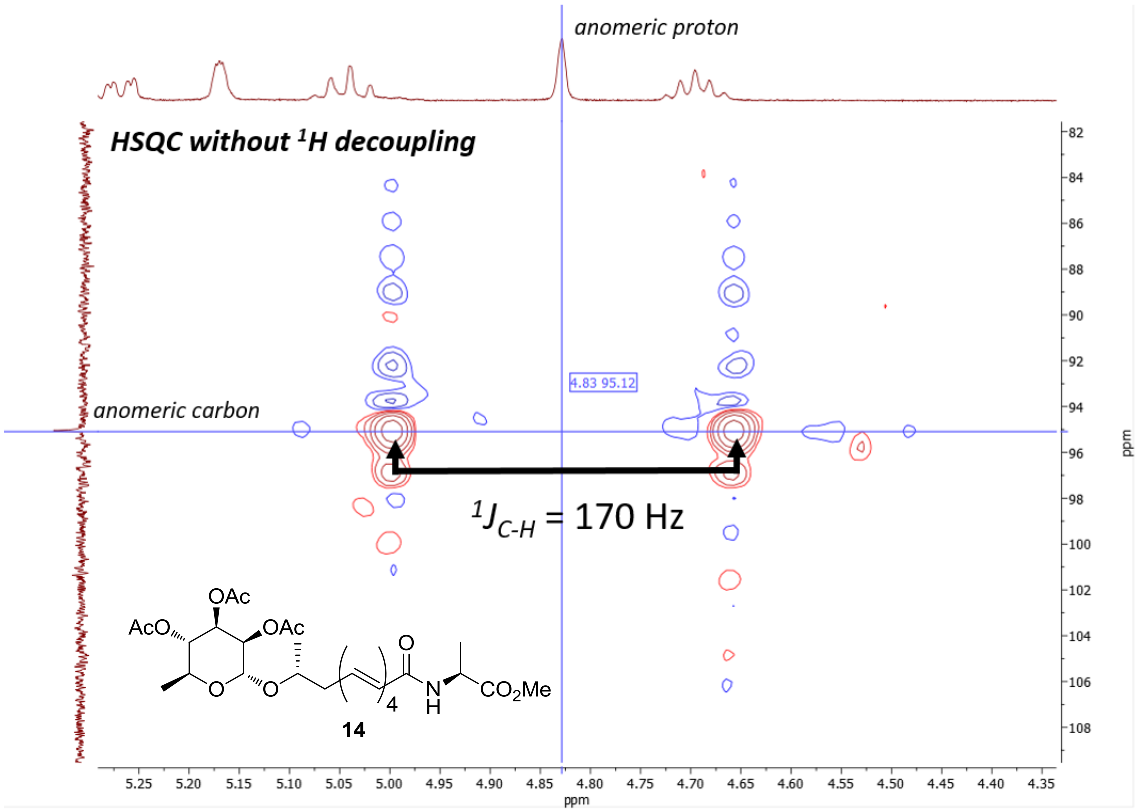

^{1}H NMR **14** (mixture of isomers) (500 MHz, $CDCl_3$)

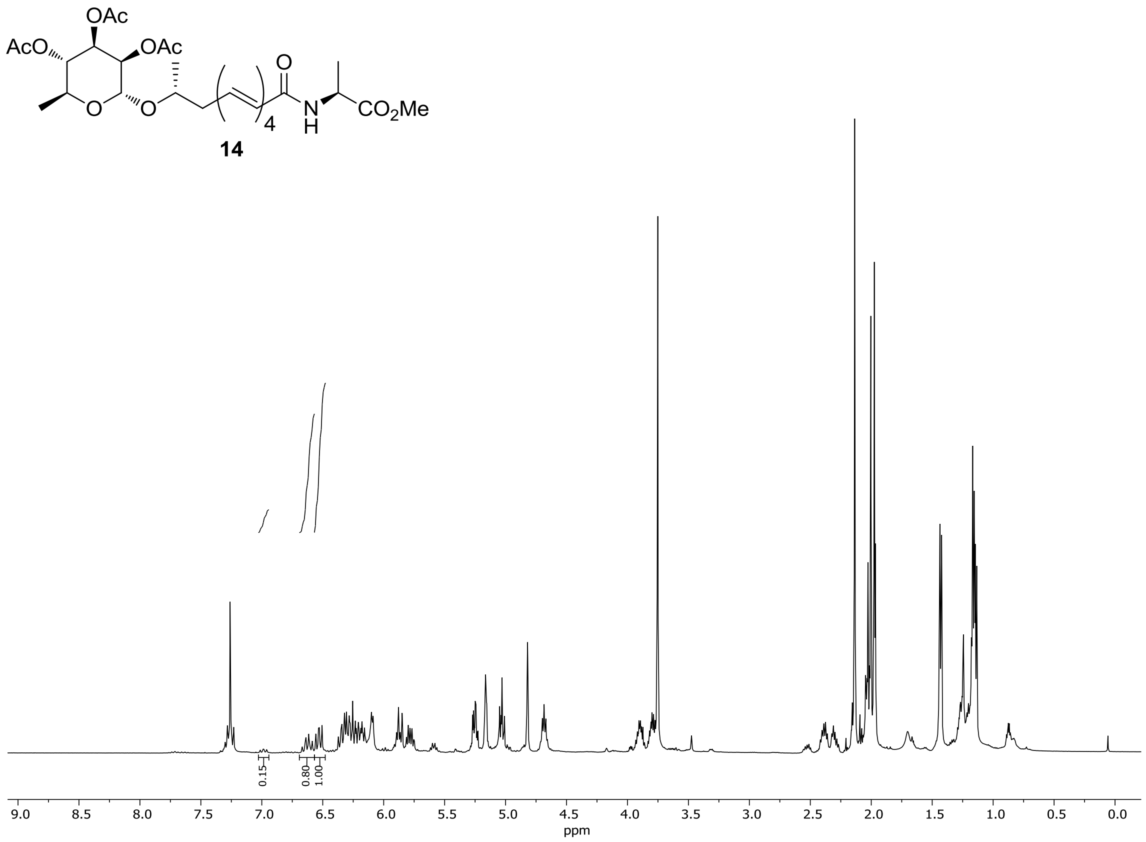

^{1}H NMR **R-P4** vaccine isomer (500 MHz, Methanol-*d*$_4$)

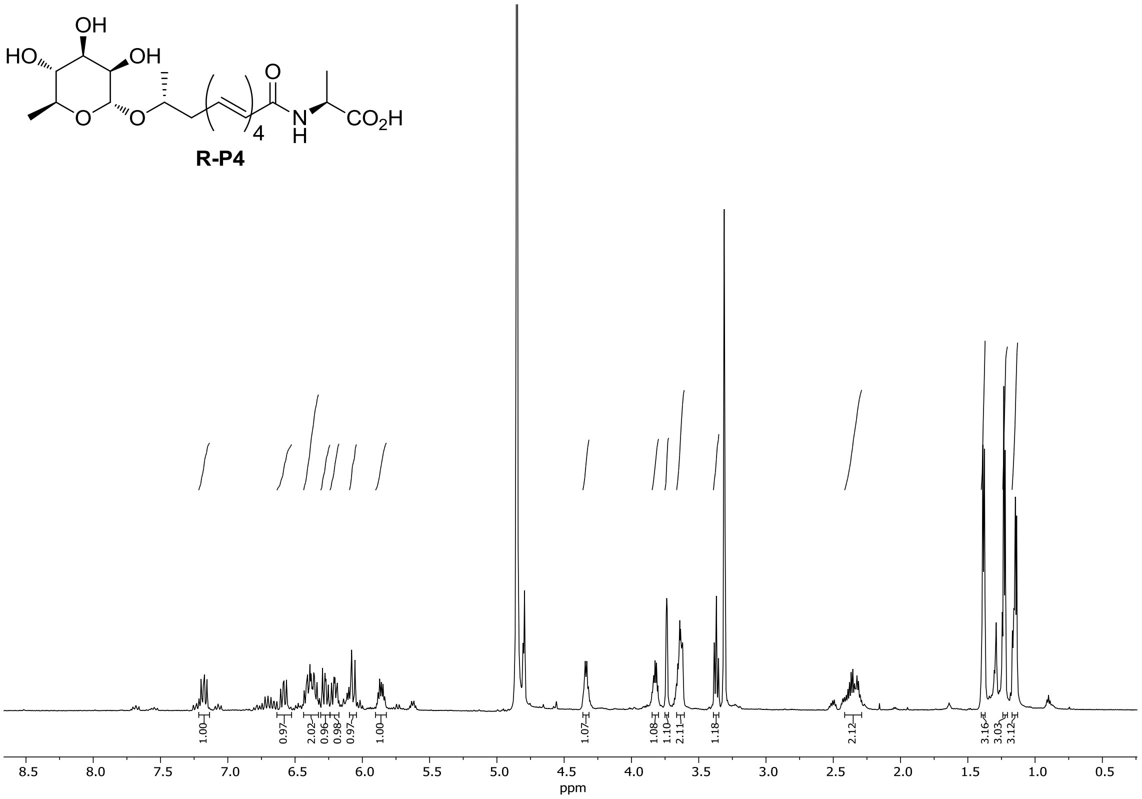

^{13}C NMR **R-P4** (126 MHz, Methanol-*d*$_4$)

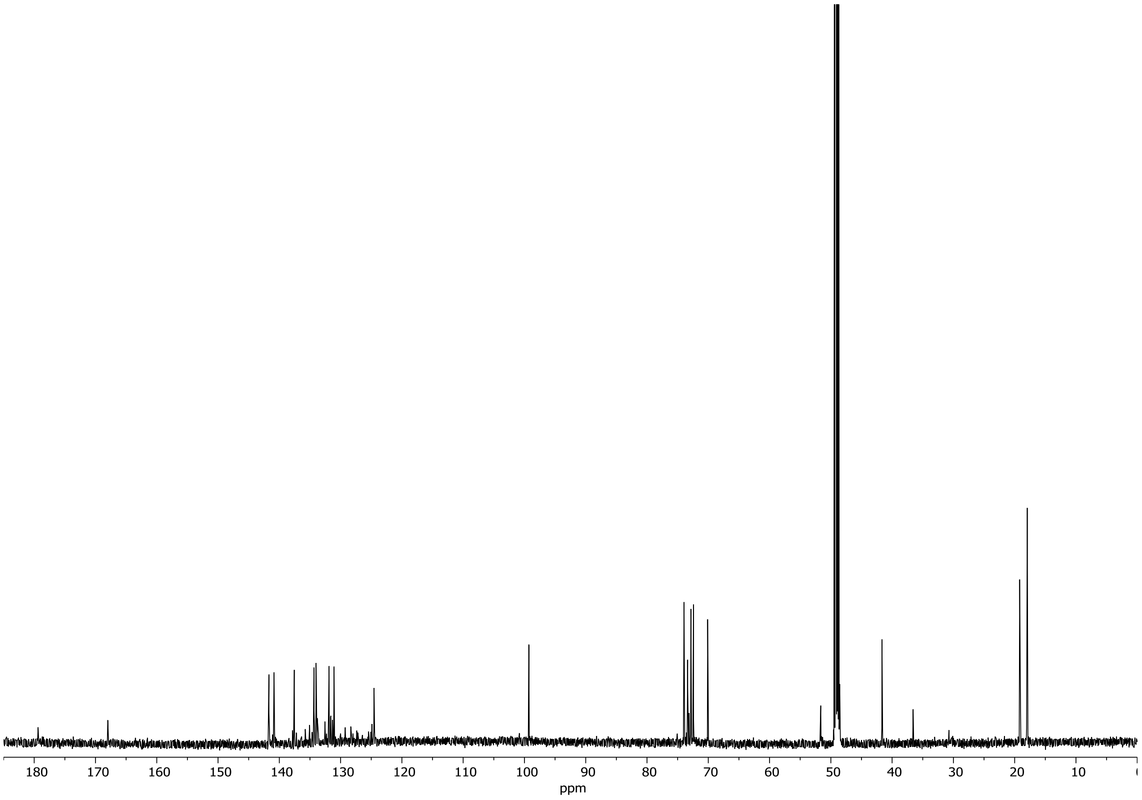

^{1}H NMR *all-E* **15** (500 MHz, $CDCl_3$)

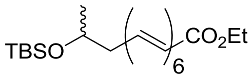

15

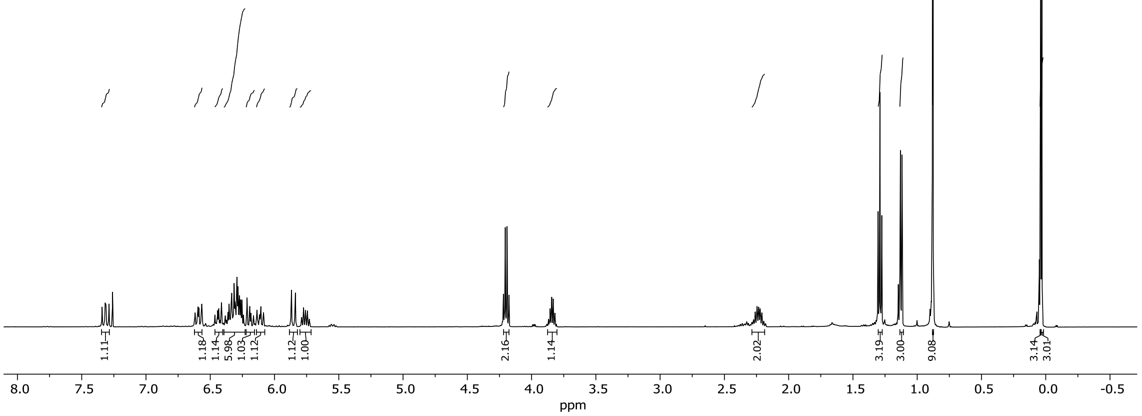

^{13}C NMR *all-E* **15** (126 MHz, $CDCl_3$)

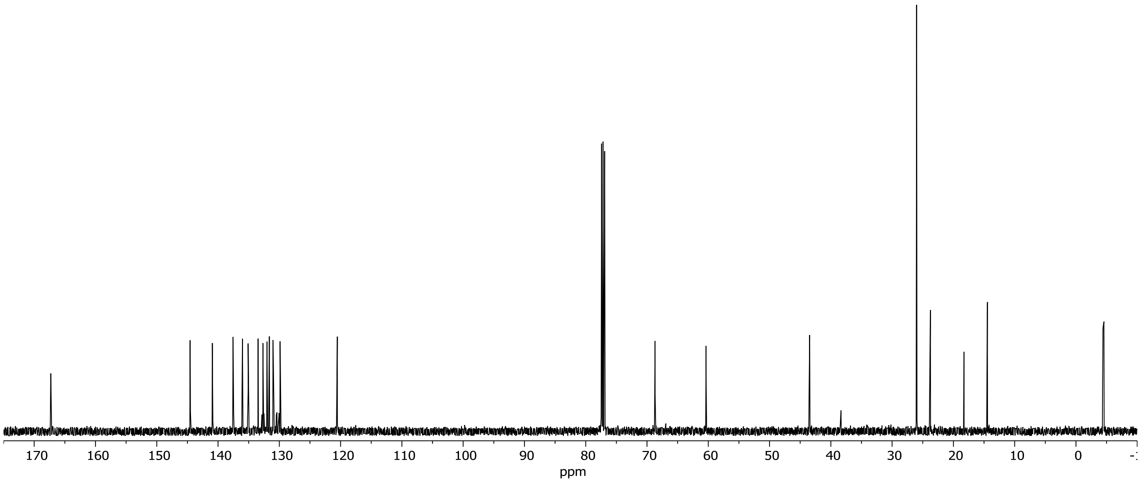

^{1}H NMR **16** (400 MHz, $CDCl_3$)

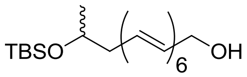

16

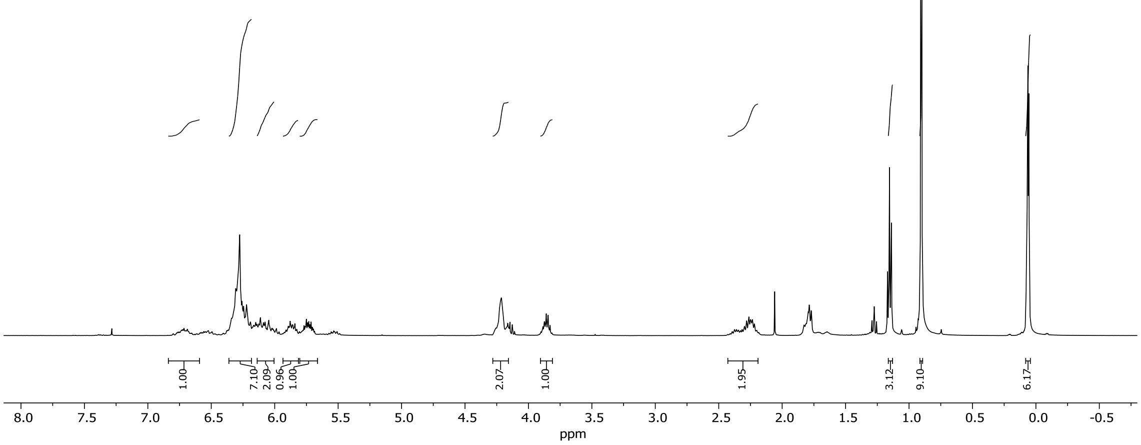

^{13}C NMR **16** (101 MHz, $CDCl_3$)

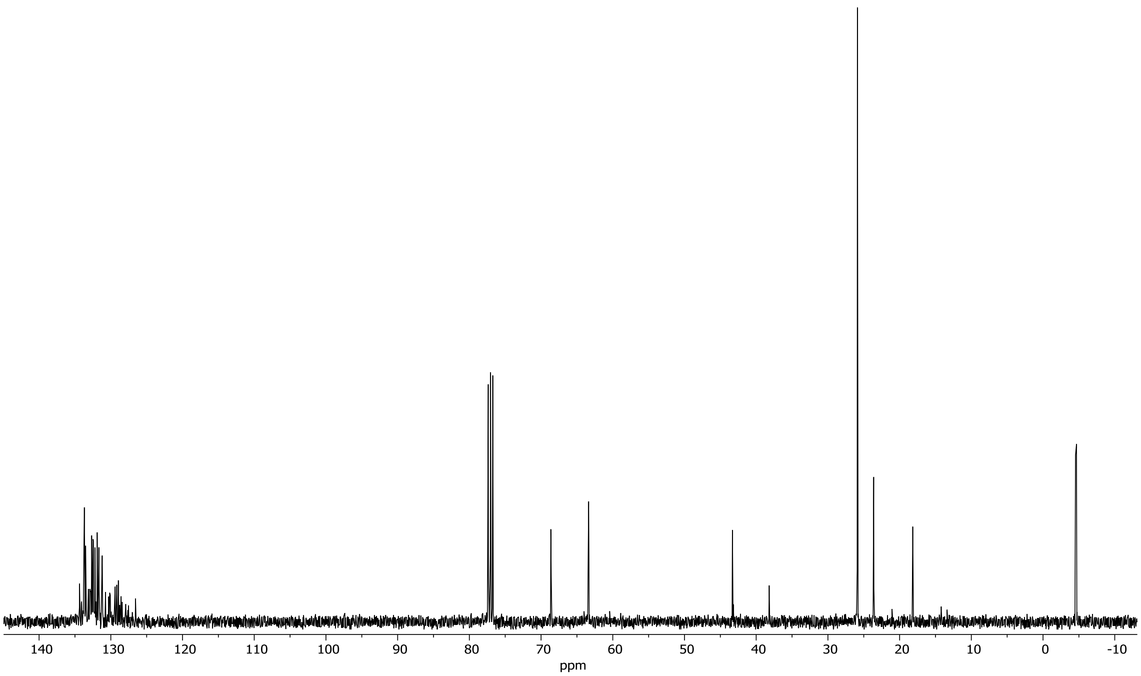

^{1}H NMR *all-E* **17** (500 MHz, $CDCl_3$)

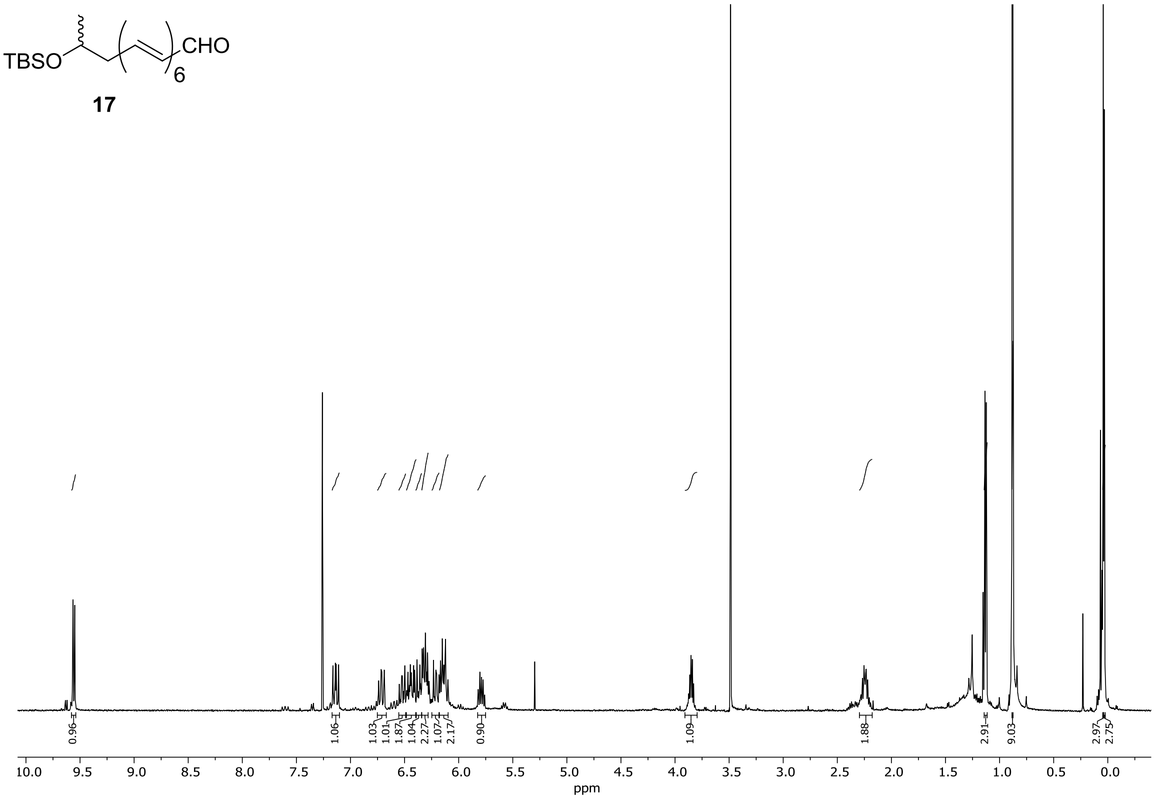

^{13}C NMR *all-E* **17** (126 MHz, $CDCl_3$)

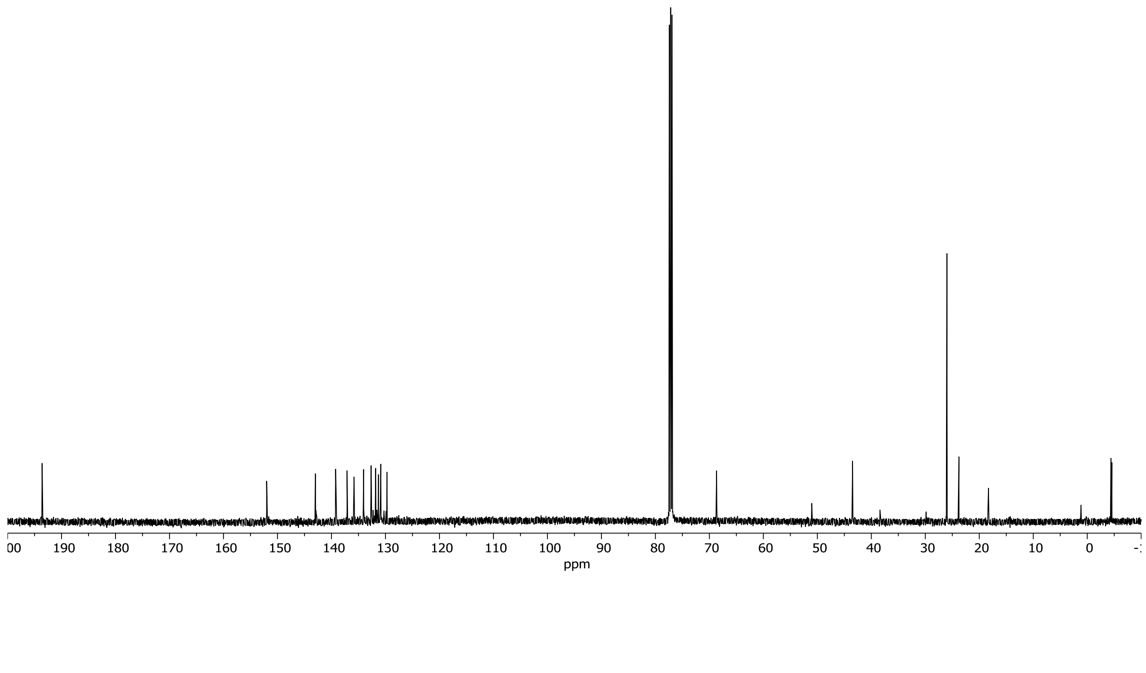

^{1}H NMR (500 MHz, $CDCl_3$) *all-E* isomer

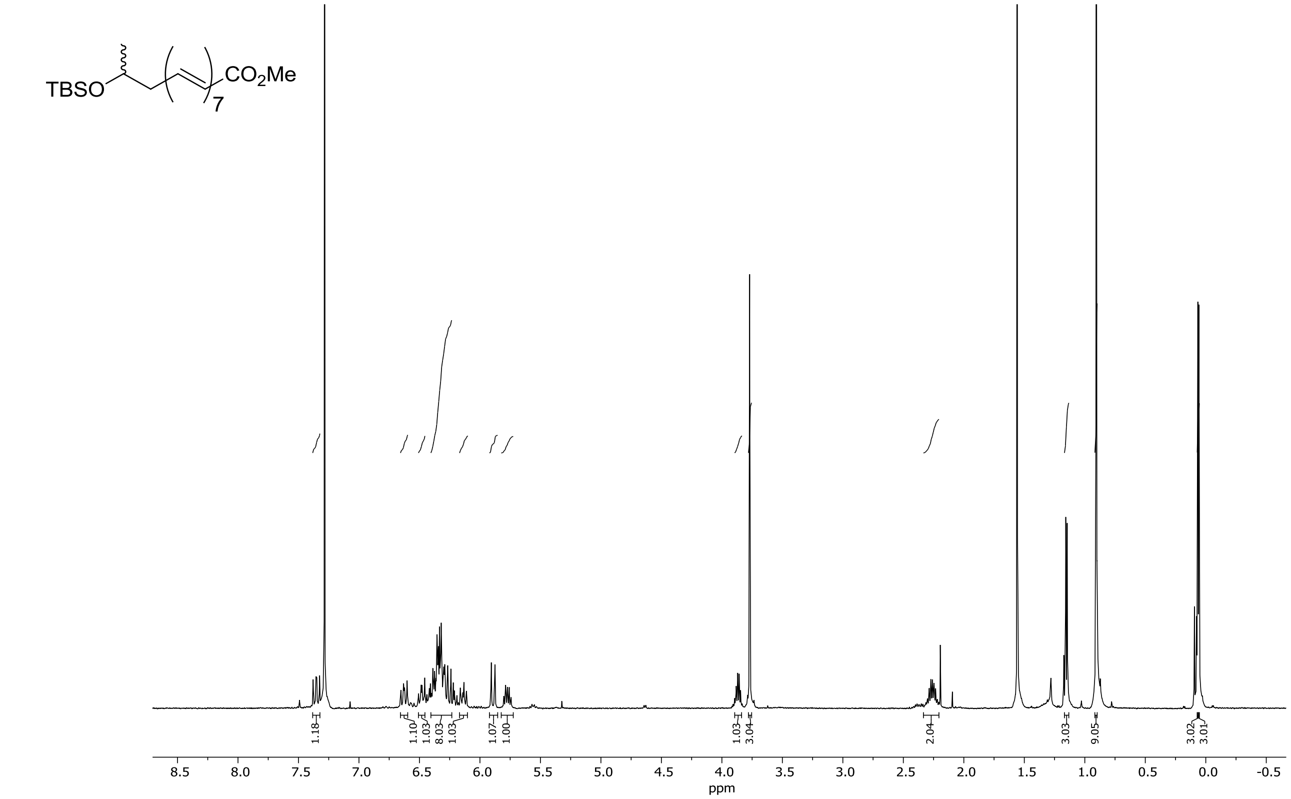

^{13}C NMR (126 MHz, $CDCl_3$) *all-E* isomer

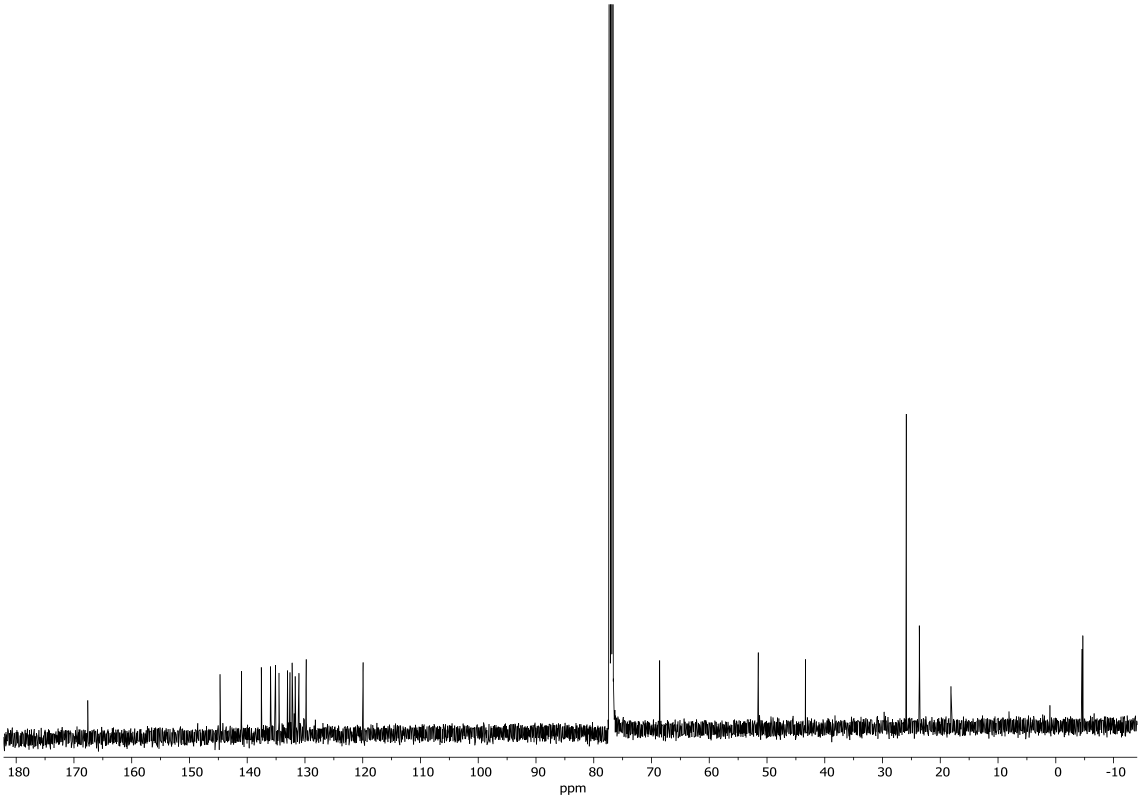

^{1}H NMR **pP7X** (500 MHz, Methanol-d_4)

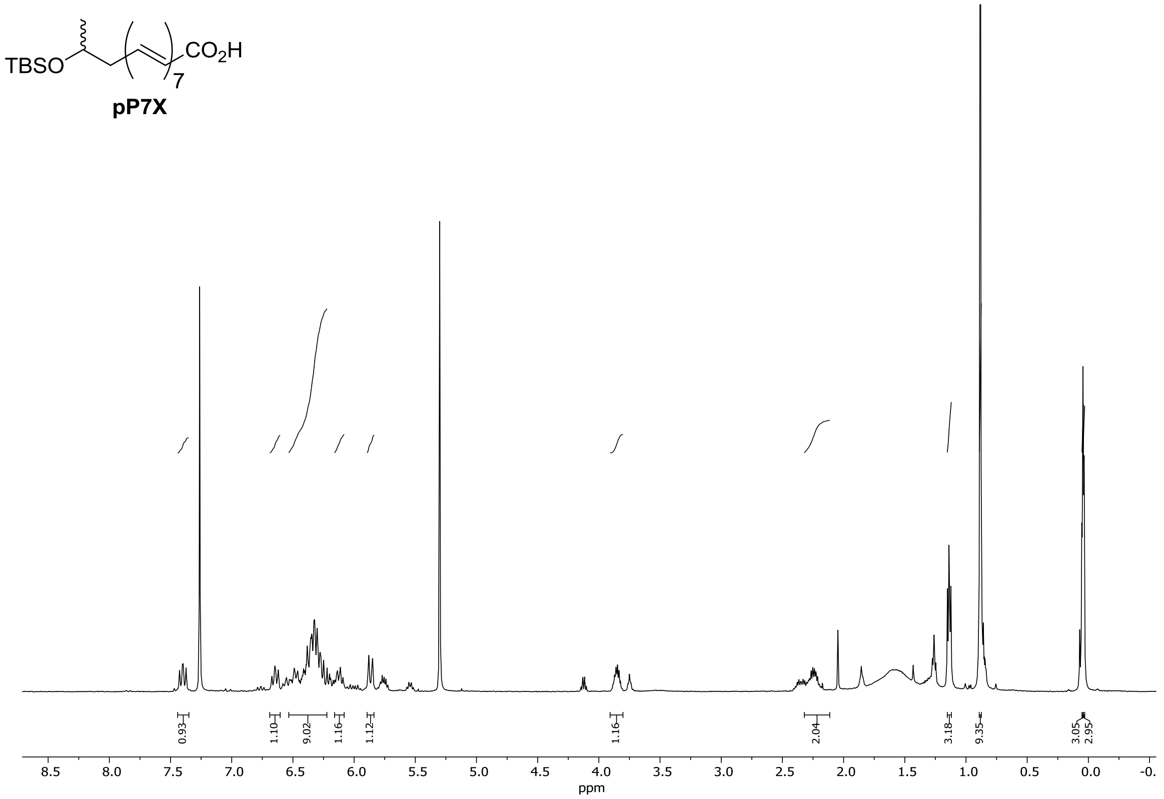

^{13}C NMR **pP7X** (126 MHz, Methanol-d_4)

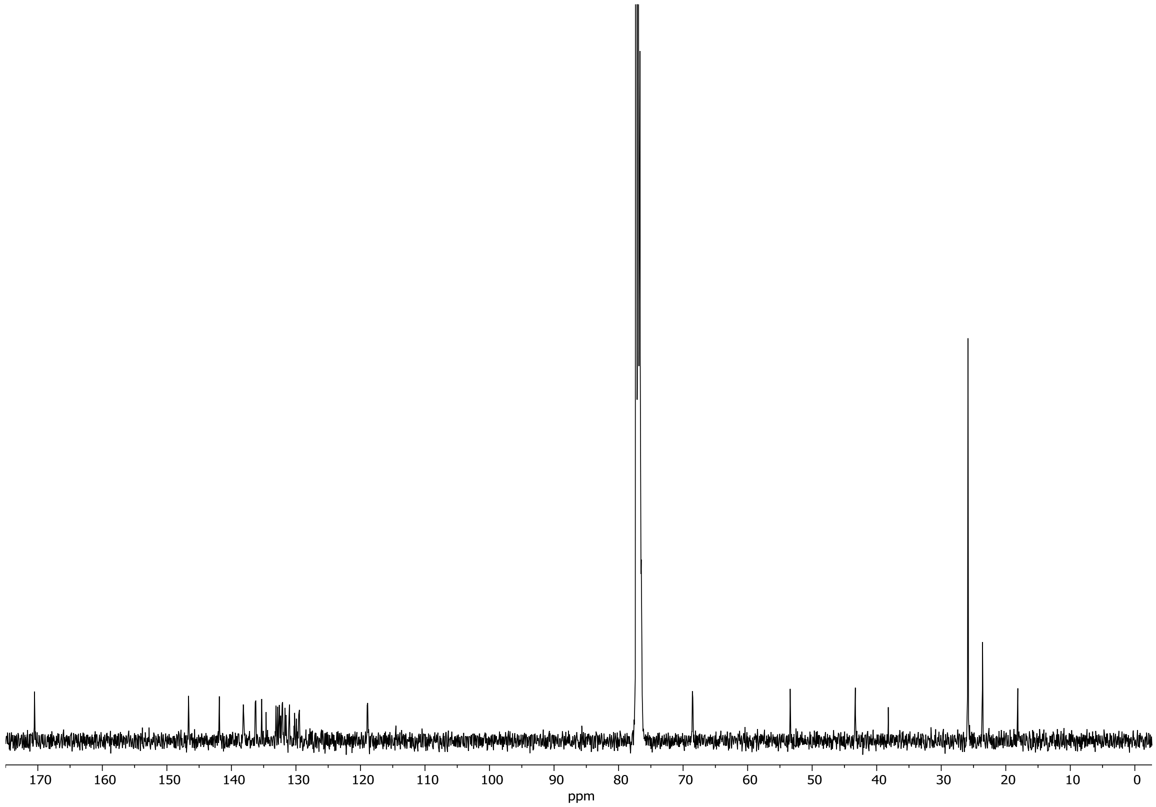

^{1}H NMR **18** (500 MHz, $CDCl_3$)

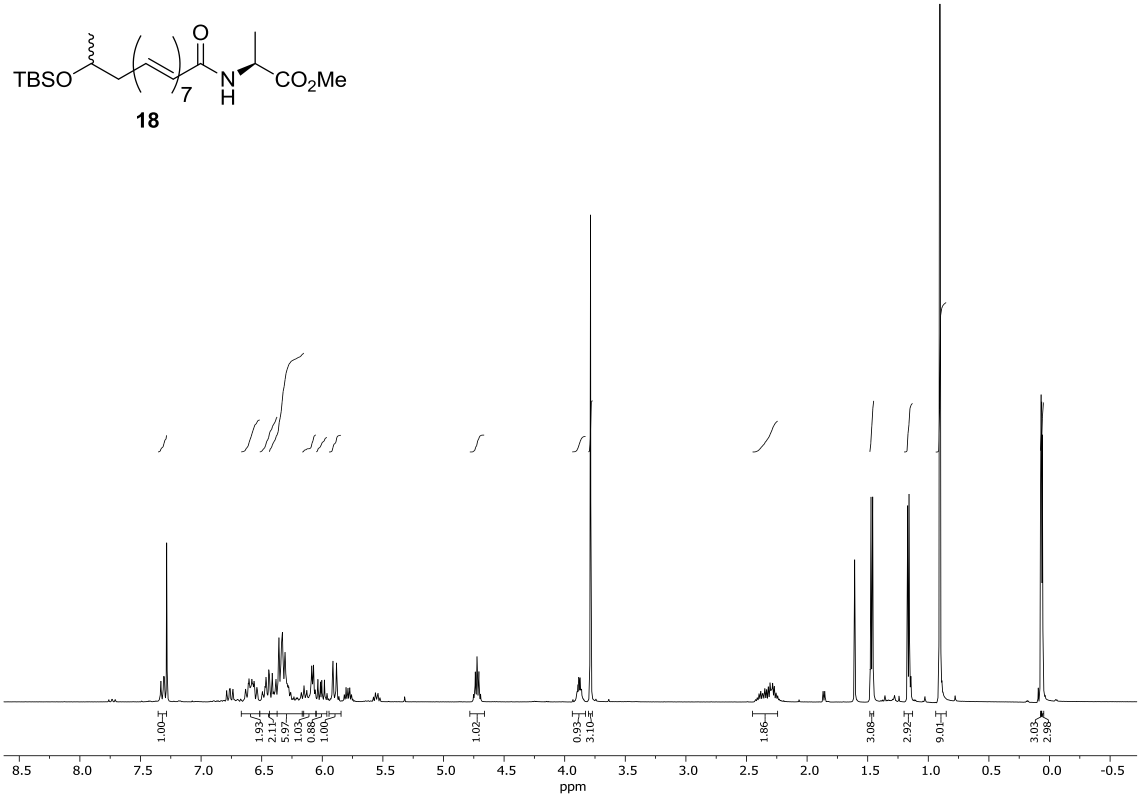

^{13}C NMR **18** (126 MHz, $CDCl_3$)

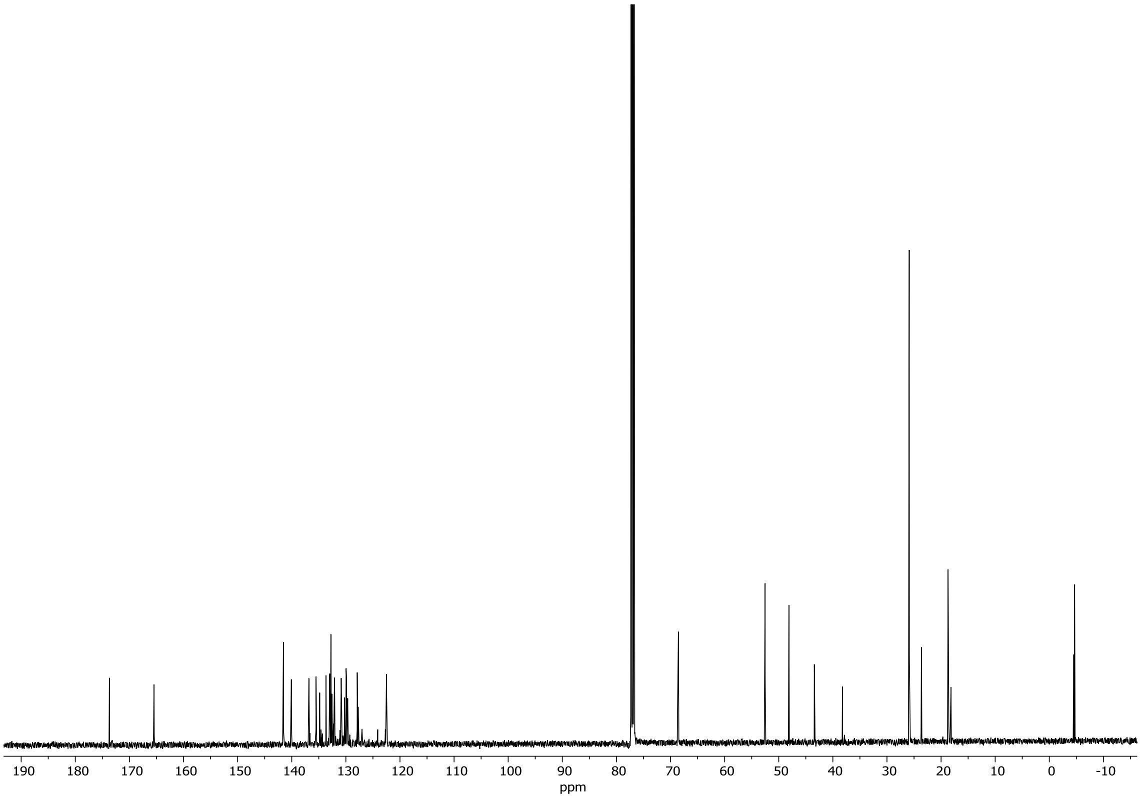

^{1}H NMR **pP7** (500 MHz, Methanol-d_4)

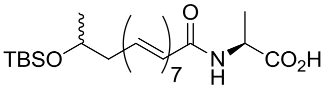

pP7

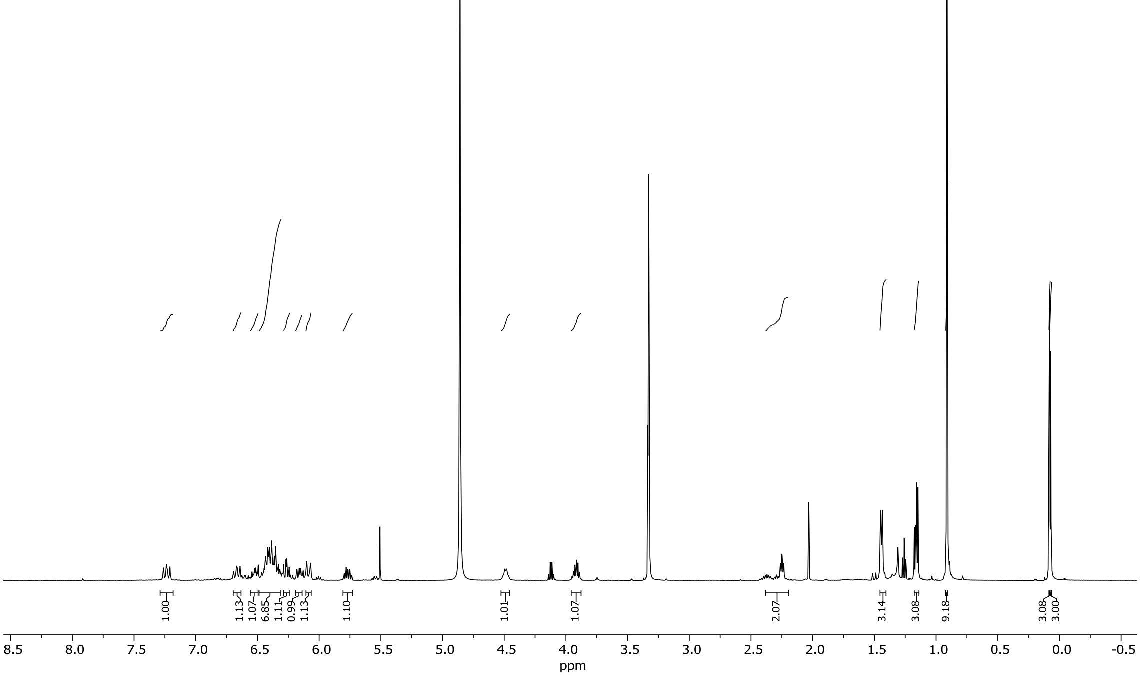

^{13}C NMR **pP7** (126 MHz, Methanol-d_4)

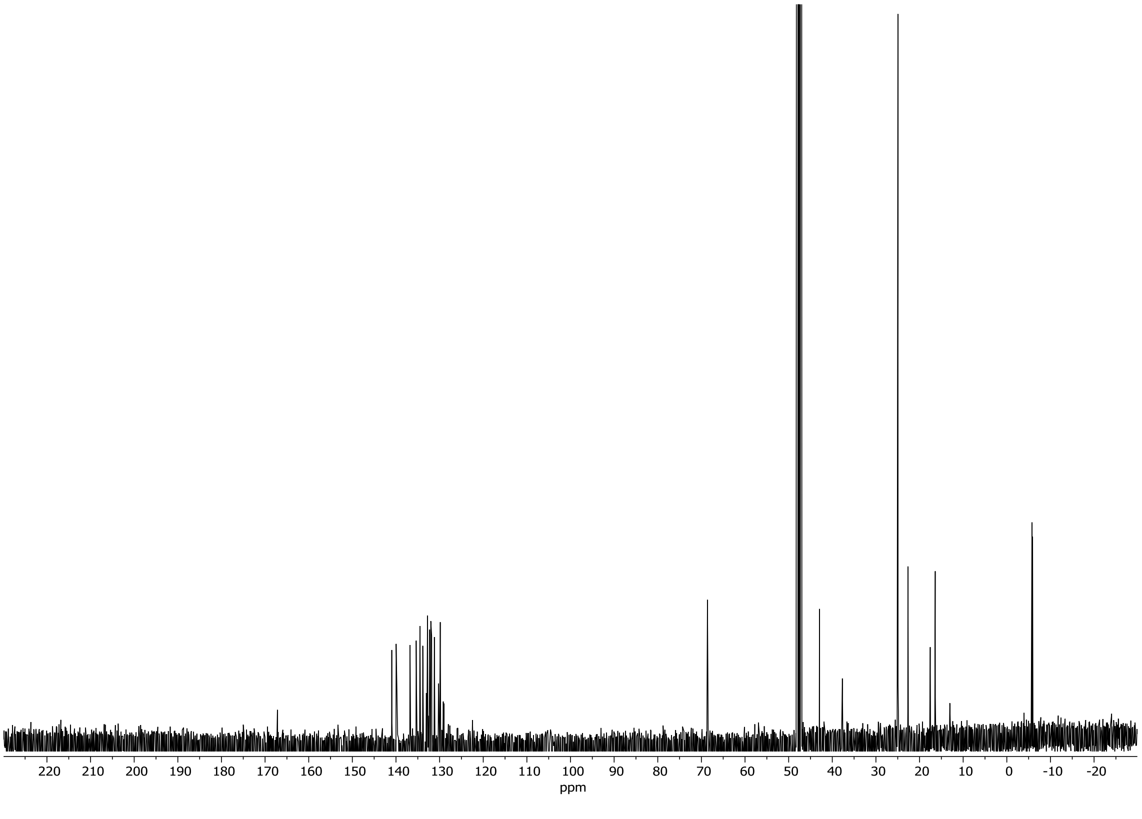

^1H NMR **P7** (500 MHz, Methanol-d_4)

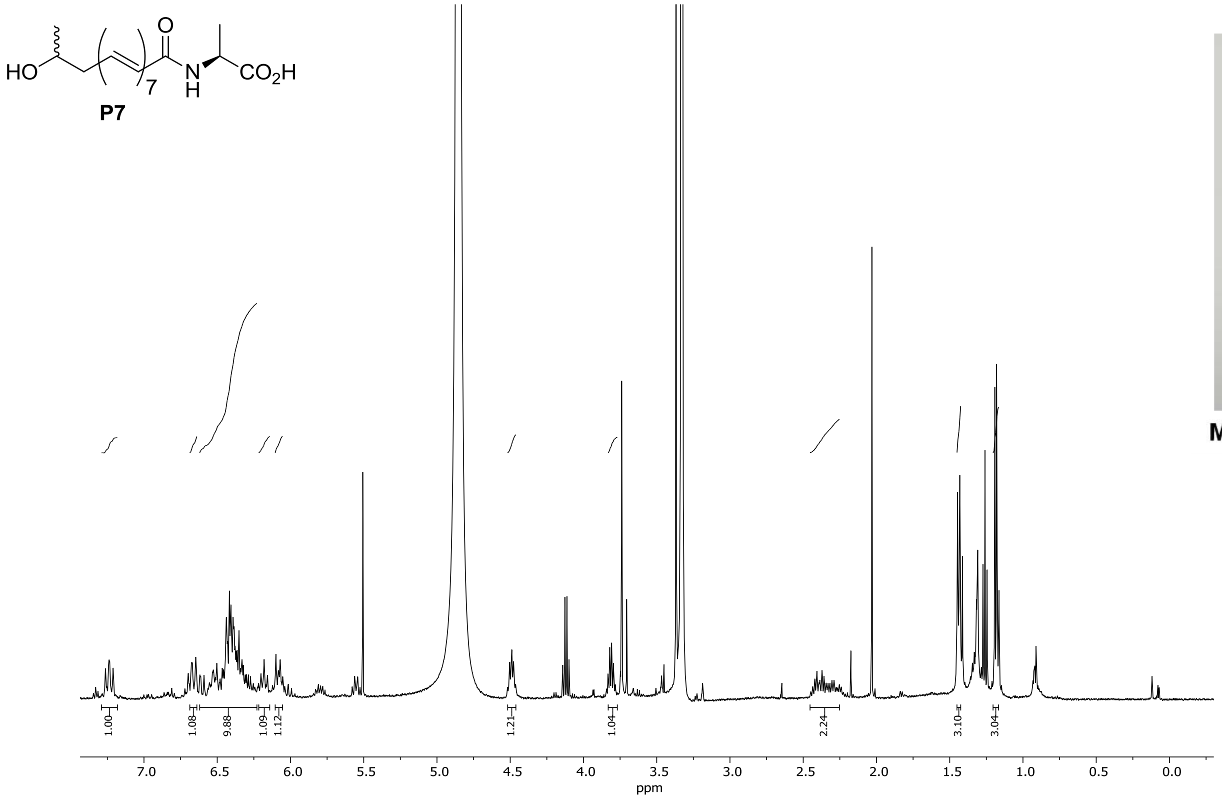

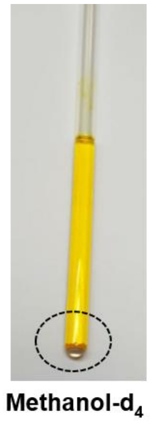

^{13}C NMR **P7** (500 MHz, Methanol-d_4)

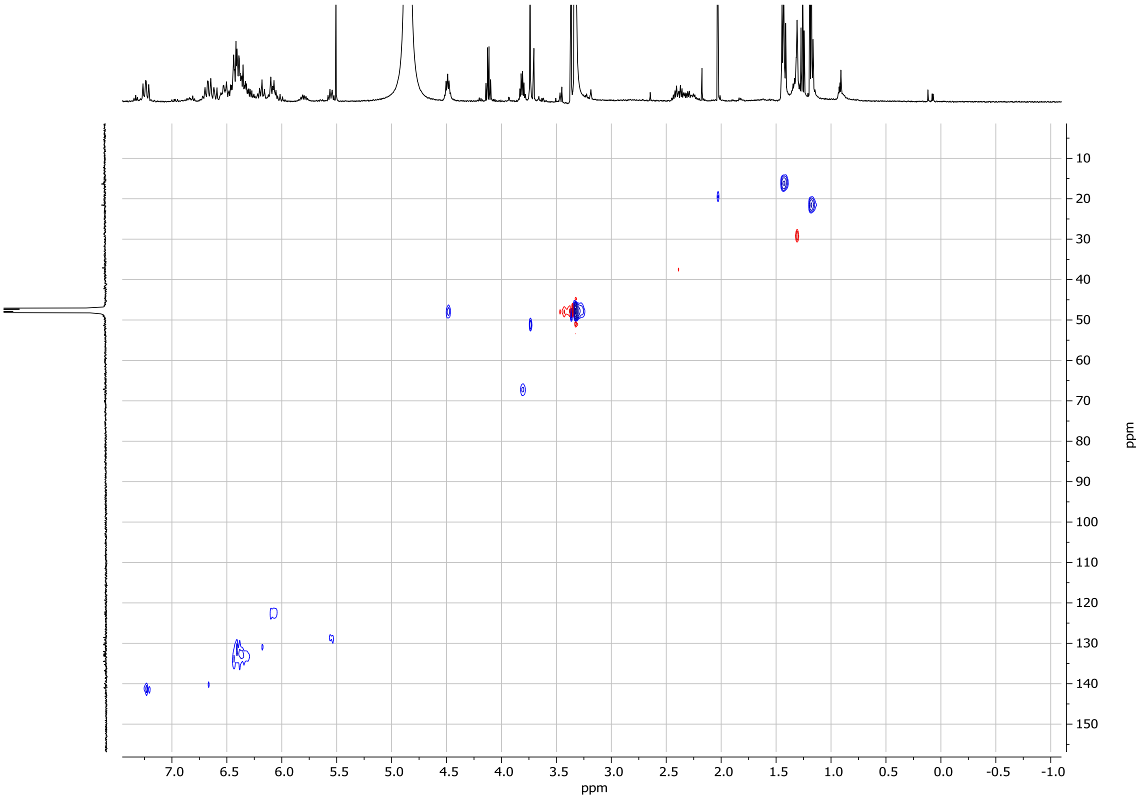

^{1}H NMR *all-E* **19** (500 MHz, $CDCl_3$)

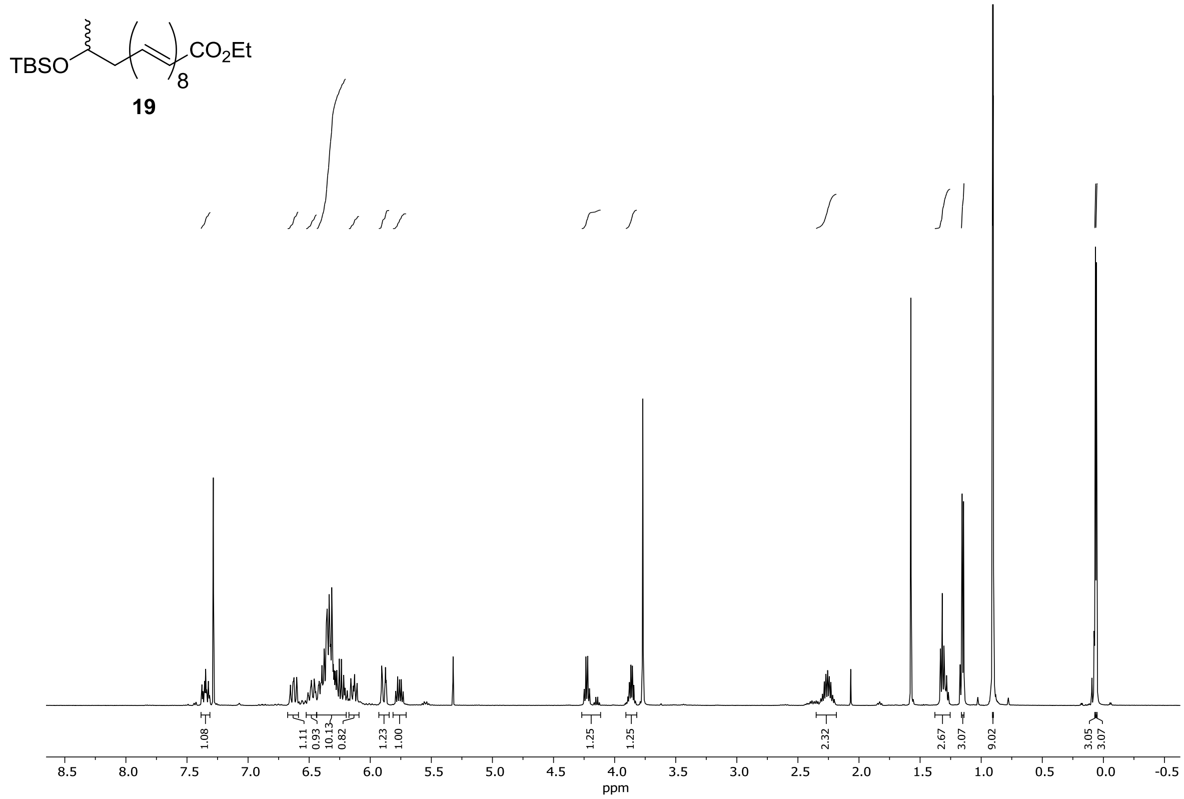

^{13}C NMR *all-E* **19** (126 MHz, $CDCl_3$)

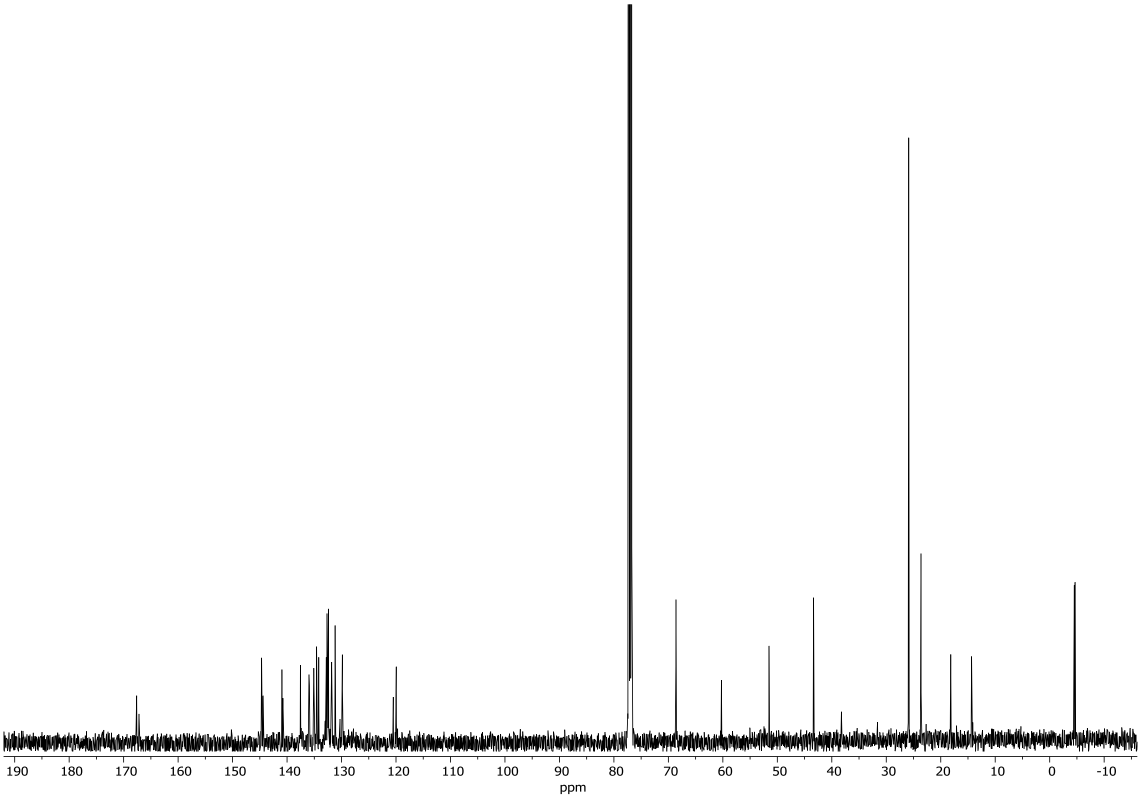

^{1}H NMR **21** (500 MHz, $CDCl_3$)

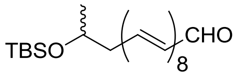

21

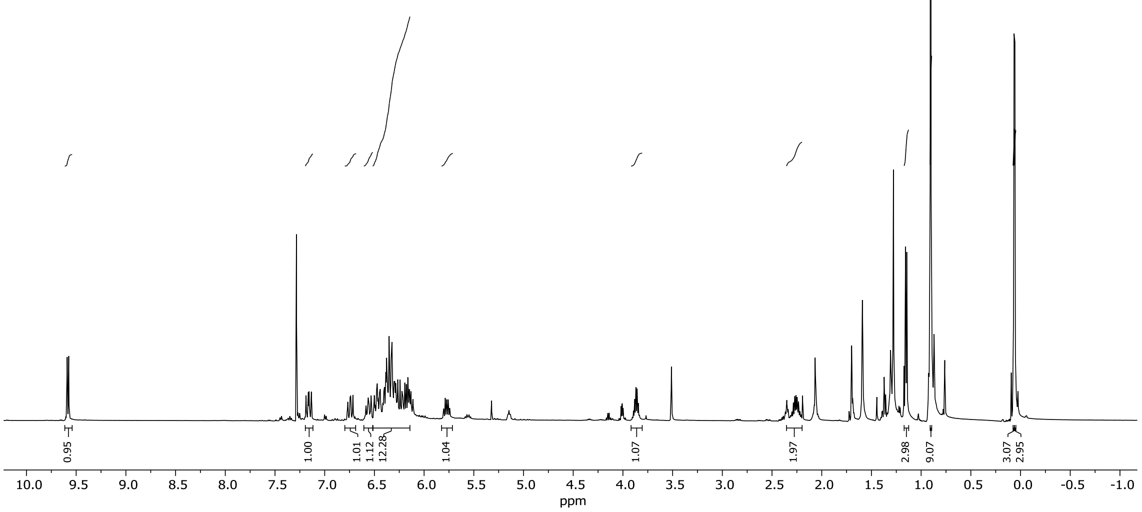

^{13}C NMR **21** (126 MHz, $CDCl_3$)

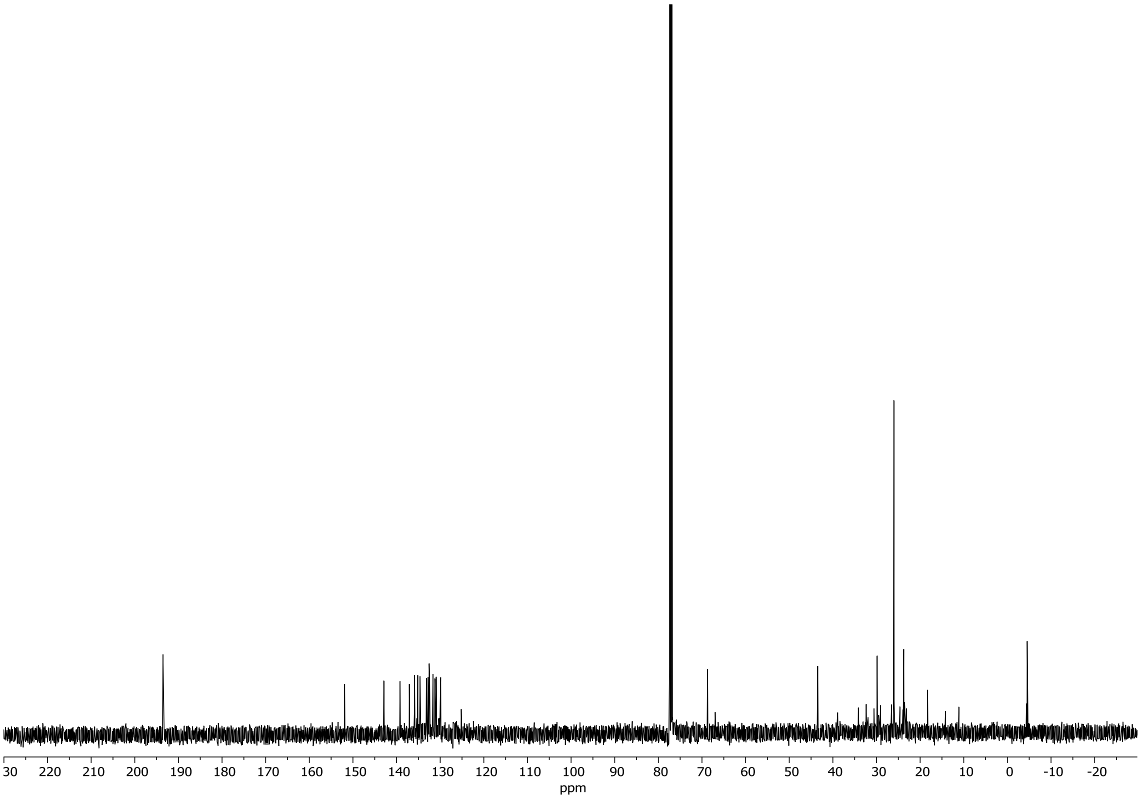

^{1}H NMR **22** (500 MHz, $CDCl_3$)

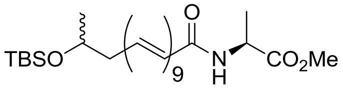

22

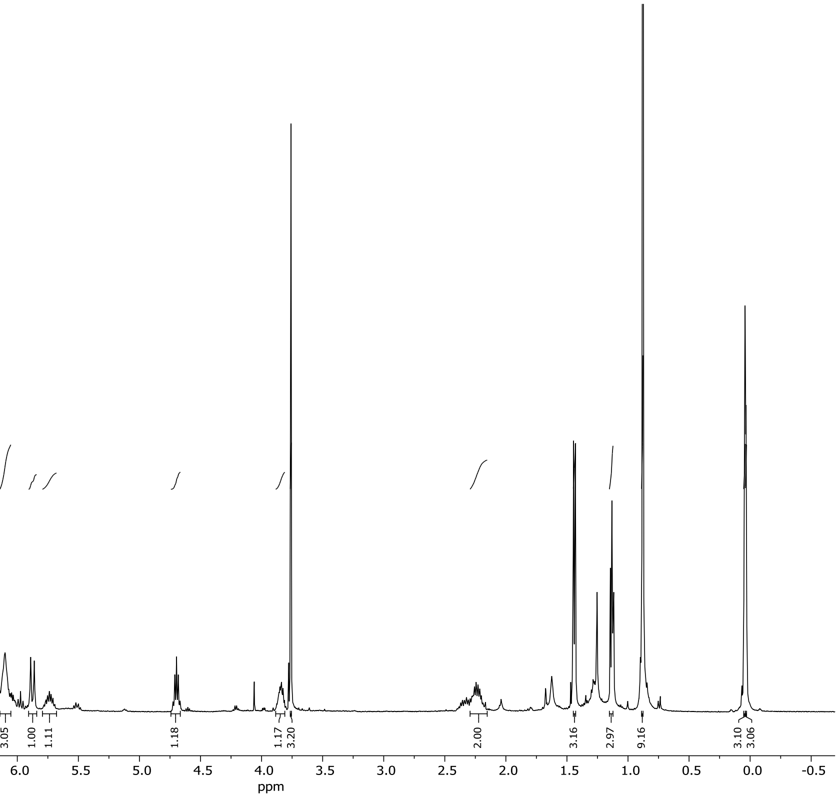

^{13}C NMR **22** (126 MHz, $CDCl_3$)

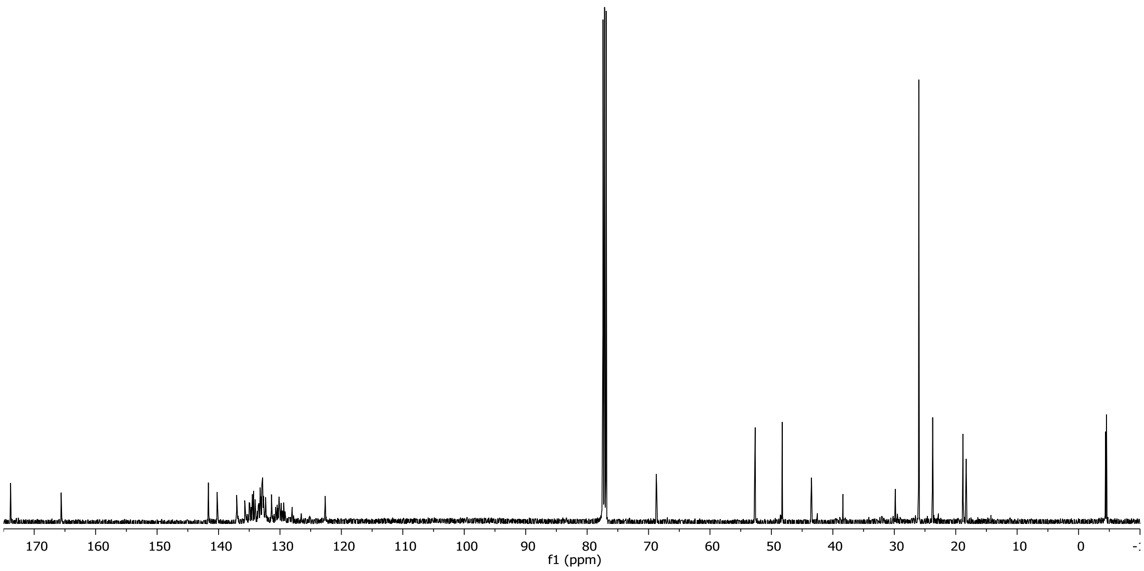

^{1}H NMR **pP9** (500 MHz, Methanol-*d_4*)

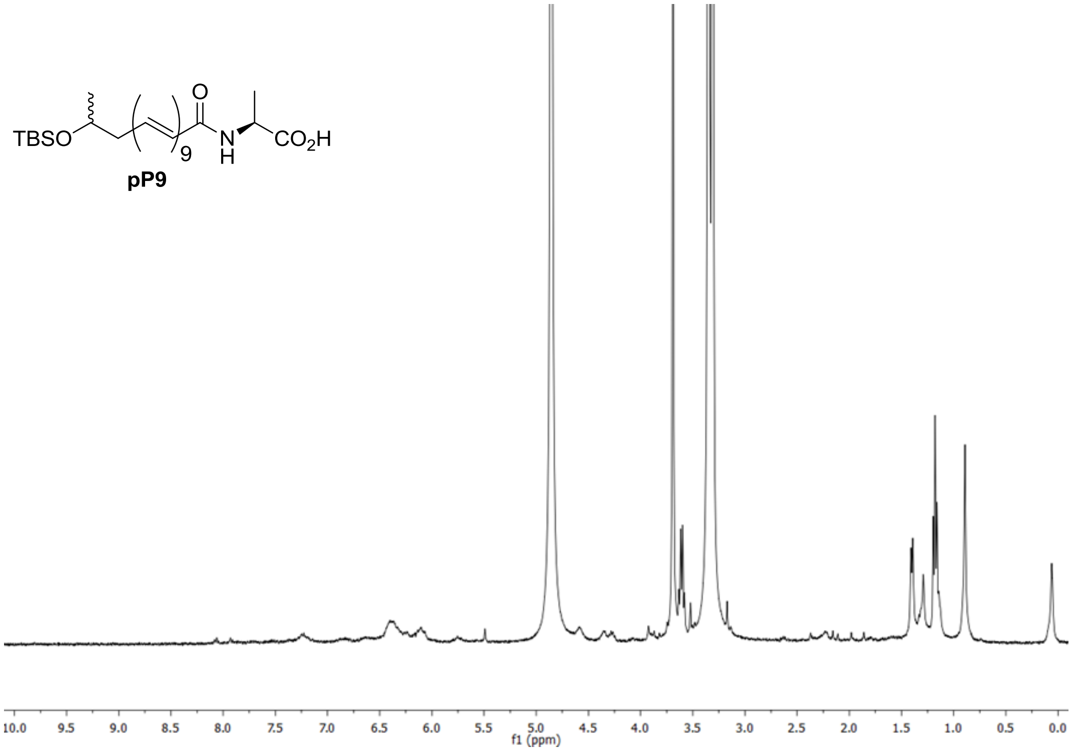

^{1}H NMR **pP9** (500 MHz, DMSO-d_6)

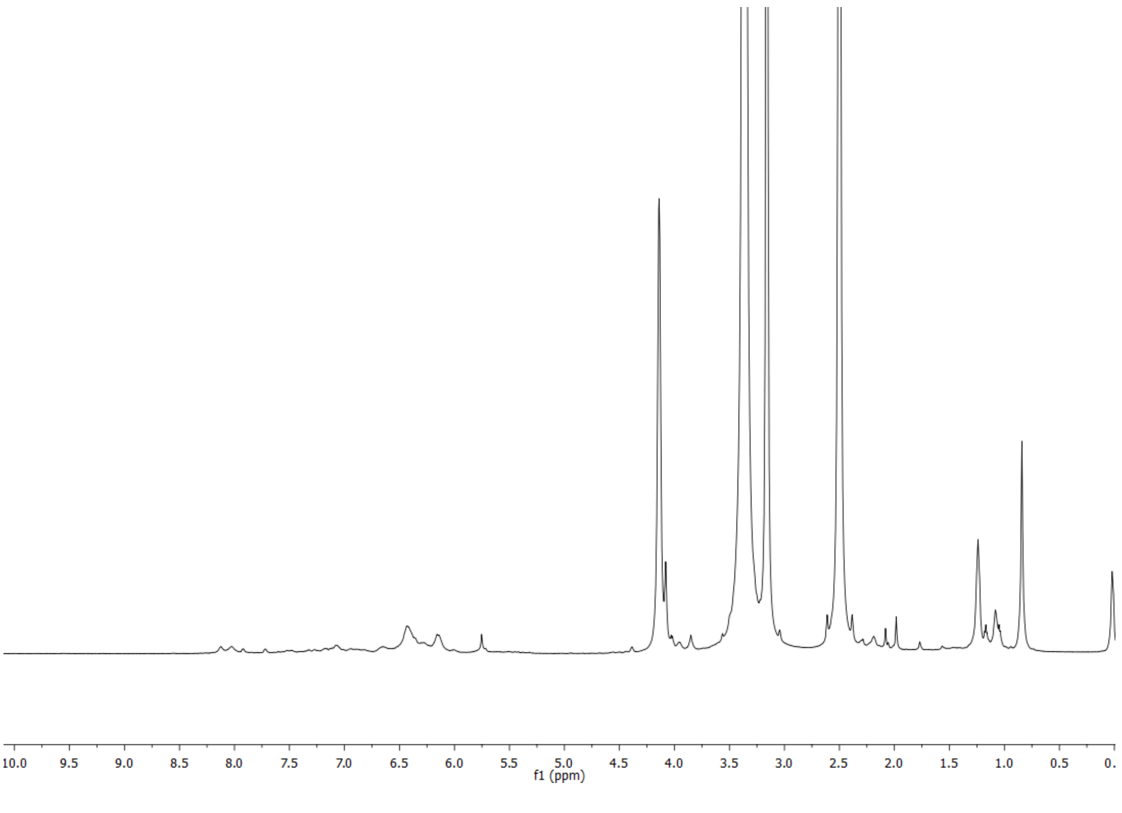

^{1}H NMR **pP9** (500 MHz, DMSO-d_6 + 0.1% TFA-d)

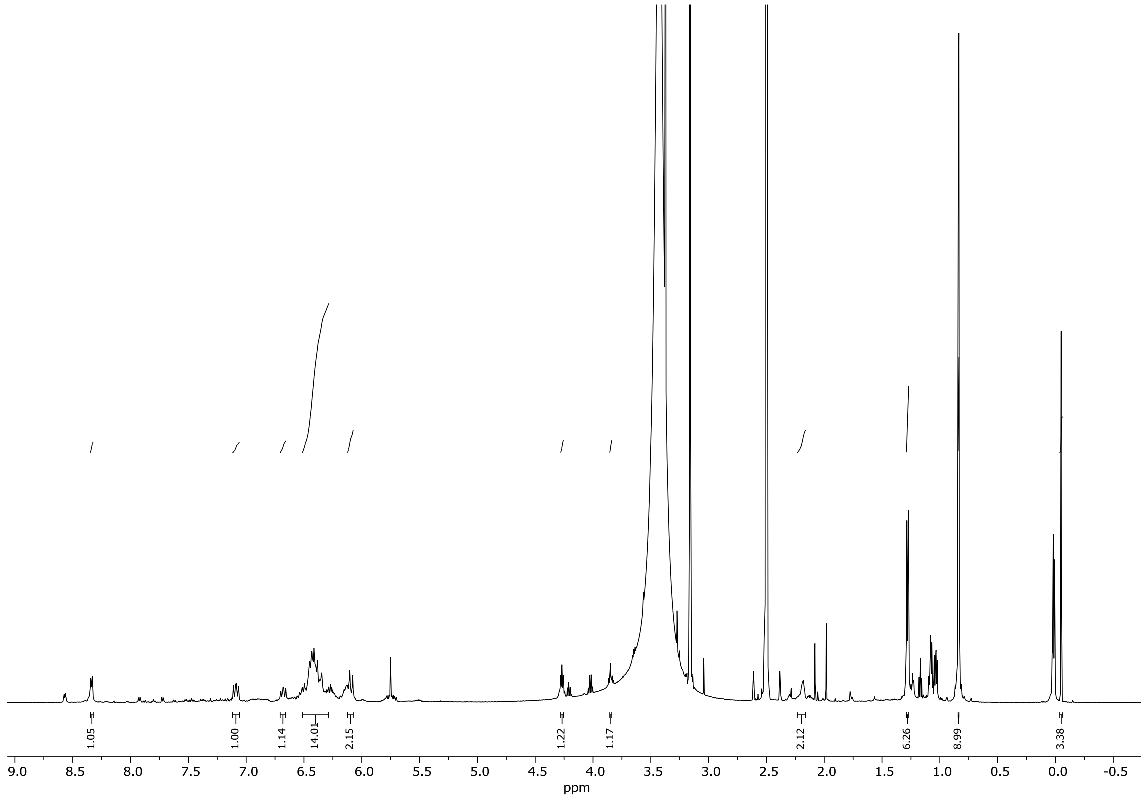

Solubility test of compound **pP9** in different deuterated solvents

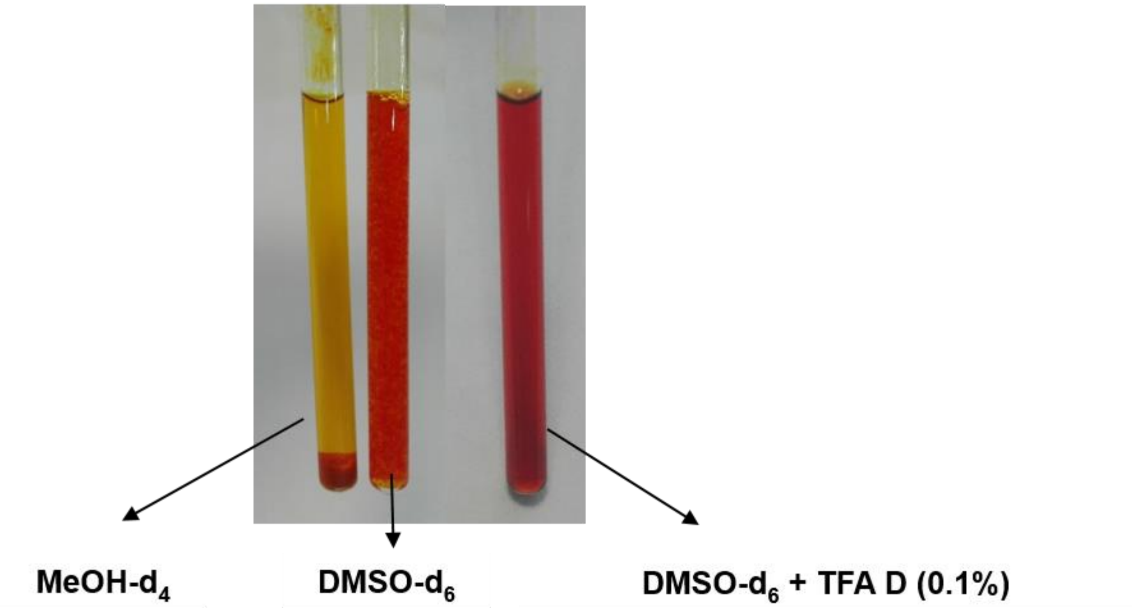

^{1}H NMR **P9** (400 MHz, Methanol-d_4)

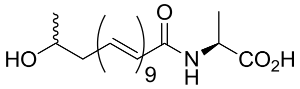

P9

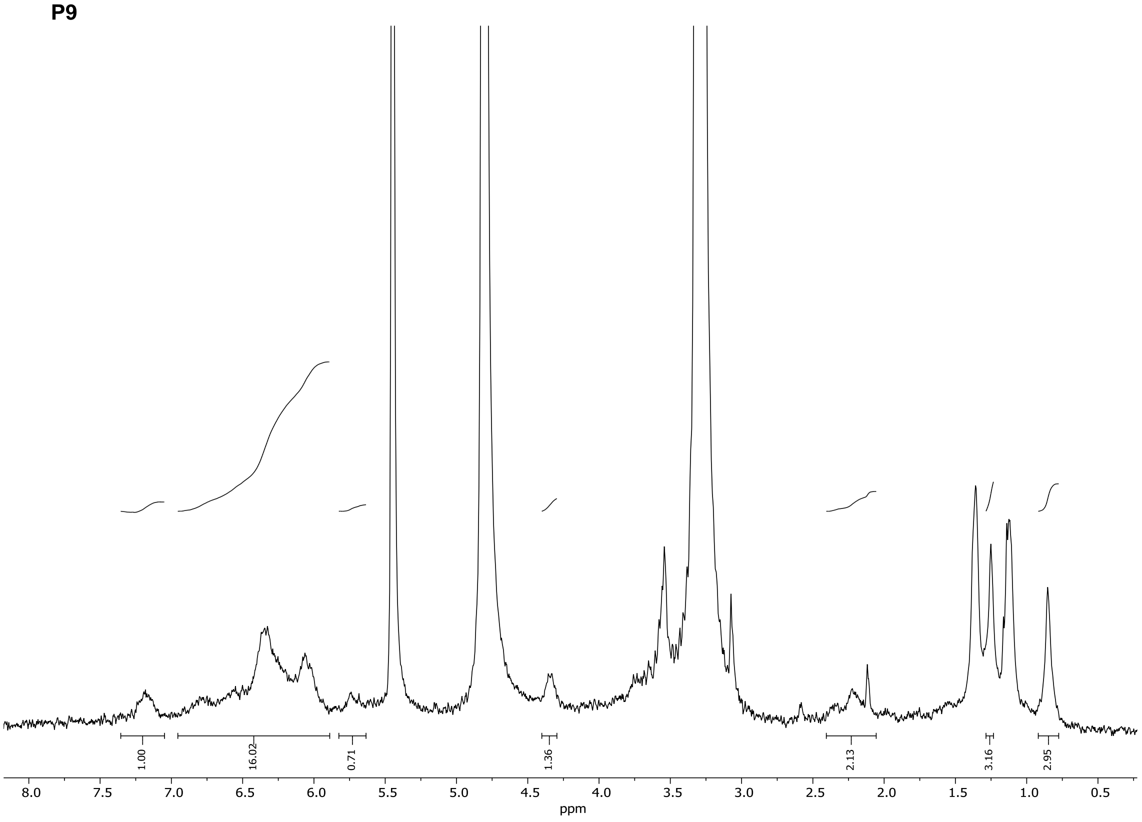

Supplementary Methods

Chemical synthesis of granadaene analogs

*Synthesis of L-alanine derivative **3***

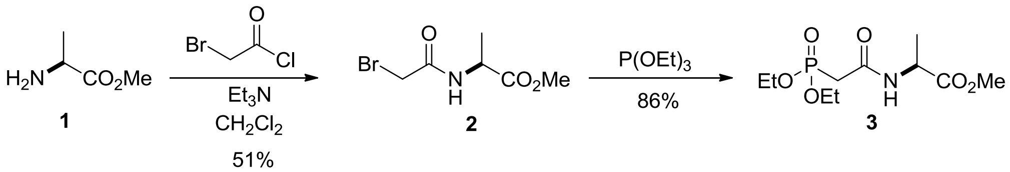

Scheme 1. Preparation of *L*-alanine derivative **3**.

<u>Synthesis of α-bromoamide **2**:</u> To a solution of *L*-alanine (**1**) (1.2 g, 8.6 mmol) in CH_2Cl_2 (50 mL), Et_3N (2.61 g, 25.8 mmol), and bromoacetyl chloride (2.03 g, 12.9 mmol) were added at 0 ºC. The mixture was stirred at room temperature for 2 h. Then, EtOAc was added and the mixture was washed with 10% aqueous HCl solution, sat. aq. $NaHCO_3$, and brine. The organic layer was dried over anhyd. Na_2SO_4, and the solvent removed. The residue was purified by flash chromatography (EtOAc/Hexane 3:7) to yield bromoamide **2** (0.99 g, 51%). Its ^{1}H and ^{13}C NMR spectra matched with those previously described[1].

<u>Preparation of phosphonate **3**:</u> To a sample of bromoamide **2** (0.99 g, 4.4 mmol), $P(OEt)_3$ (0.81 g, 4.9 mmol) was added, and the mixture was stirred at 100 ºC for 12 h. Phosphonate **3** was obtained without further purification (1.05 g, 86%). Brown oil. **^{1}H NMR (400 MHz, $CDCl_3$)** δ 7.22 (d, J = 7.3 Hz, 1H), 4.56 (quint, J = 7.2 Hz, 1H), 4.21–4.10 (m, 4H), 3.73 (s, 3H), 2.87 (d, $^2J_{H\text{-}P}$ = 20.7 Hz, 2H), 1.41 (d, J = 7.2 Hz, 3H), 1.33 (td, $^3J_{H\text{-}H}$ = 7.1; $^3J_{H\text{-}P}$ = 1.6 Hz, 6H). **^{13}C NMR (101 MHz, $CDCl_3$)** δ 172.9 (C), 163.7 (d, $^2J_{C\text{-}P}$ = 3.9 Hz, C), 62.9 (d, $^2J_{C\text{-}P}$ = 6.1 Hz, CH_2), 62.8 (d, $^2J_{C\text{-}P}$ = 6.2 Hz, CH_2), 52.4 (CH_3), 48.4 (CH), 35.1 (d, $^1J_{C\text{-}P}$ = 130.9 Hz, CH_2), 18.0 (CH_3), 16.3 (d, $^3J_{C\text{-}P}$ = 6.0 Hz, CH_3). **^{31}P NMR (202 MHz, $CDCl_3$)** δ 22.25. **HRMS (ESI):** $[M+Na]^+$ calcd. $C_{10}H_{20}NPO_6Na$: 304.0920, found: 304.0928.

Synthesis of pP1

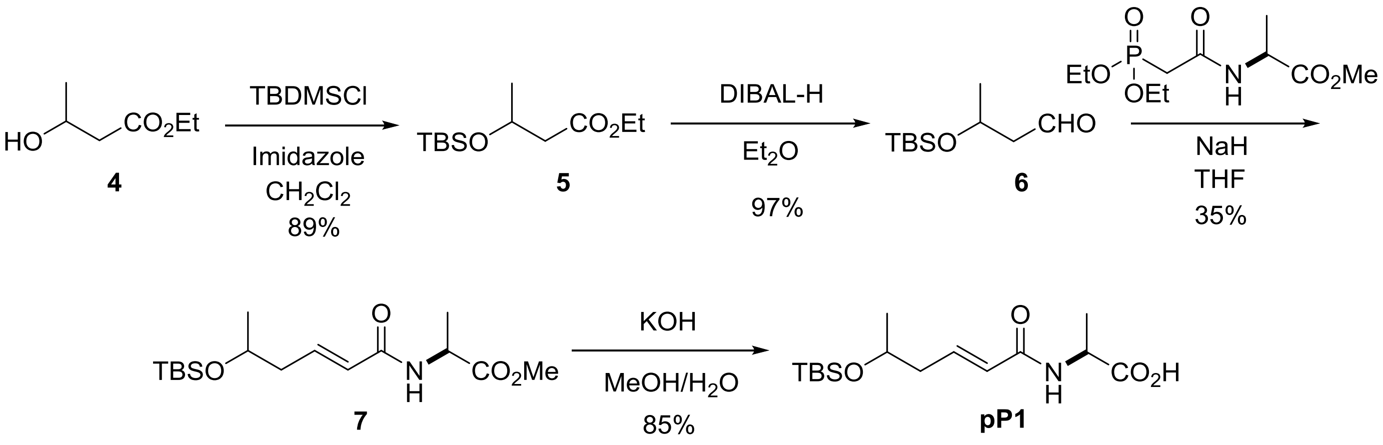

Scheme 2. Preparation of **pP1.**

Preparation of aldehyde **6**: To a solution of ester **5** (500 mg, 2.00 mmol) in Et_2O (2 mL), DIBAL-H (2.2 mL, 2.2 mmol, 1M in THF) was added, and the mixture was stirred at -78 ºC for 40 min. Then, the reaction was quenched with H_2O, diluted with EtOAc, and washed with 10% aqueous HCl solution, and brine. The mixture was dried over anhyd. Na_2SO_4 and the solvent removed. The residue was purified by flash chromatography (EtOAc/Hexane 1:9) to yield **6** (400 mg, 97%). Its ^{1}H and ^{13}C NMR spectra matched with those previously described[2].

Synthesis of ester **7**: According to GP-1, ester **7** was synthesized from aldehyde **6**, with a 35% yield. (EtOAc/Hexane 6:4). Colorless oil; **^{1}H NMR (500 MHz, $CDCl_3$)** δ 6.82 (dt, *J* = 15.2, 7.5 Hz, 1H), 6.15-6.04 (m, 1H), 5.83 (d, *J* = 15.3 Hz, 1H), 4.68 (quint, *J* = 7.2, 1H), 3.95–3.87 (m, 1H), 3.75 (s, 3H), 2.39–2.22 (m, 2H), 1.43 (d, *J* = 7.1 Hz, 3H), 1.15 (d, *J* = 6.0 Hz, 3H), 0.87 (s, 9H), 0.04 (s, 6H); **^{13}C NMR (125 MHz, $CDCl_3$)** δ 173.8 (C), 165.2 (C), 142.2 (CH), 125.3 (CH), 67.8 (CH), 52.6 (CH_3), 48.1 (CH), 42.6 (CH_2), 26.0 (CH_3), 23.8 (CH_3), 18.7 (CH_3), 18.2 (C), –4.42 (CH_3), –4.44 (CH_3); **HRMS (ESI):** $[M+H]^+$ calcd. $C_{16}H_{32}NO_4Si$: 330.2095, found: 330.2087.

Deprotection of compound **7**: According to GP-4, acid **pP1** was synthesized from compound **7**, with a 85% yield. (MeOH/CH_2Cl_2 1:9). Light brown oil; **^{1}H NMR (500 MHz, $CDCl_3$)** δ 9.49 (bs, 1H), 6.82 (dt, *J* = 15.2, 7.5 Hz, 1H), 6.57–6.36 (m, 1H), 5.82 (d, *J* = 15.3 Hz, 1H), 4.73–4.51 (m, 1H), 3.95–3.86 (m, 1H), 2.38–2.21 (m, 2H), 1.46 (d, *J* = 7.1 Hz, 3H), 1.13 (d, *J* = 6.1 Hz, 3H), 0.86 (s, 9H), 0.03 (s, 3H), 0.02 (s, 3H). **^{13}C NMR (126 MHz, $CDCl_3$)** δ 175.9 (C), 166.2 (C), 143.1 (CH), 124.9 (CH), 67.8 (CH), 48.5 (CH), 42.6 (CH_2), 26.0 (CH_3), 23.8 (CH_3), 18.3 (CH_3), 18.2 (C), –4.4 (CH_3), –4.6 (CH_3). **HRMS (ESI)**: $[M+H]^+$ calcd. $C_{15}H_{30}NO_4Si$: 316.1938, found: 316.1933. **Solubility**: CH_2Cl_2, $CHCl_3$ and DMSO among others.

Synthesis of R-P4

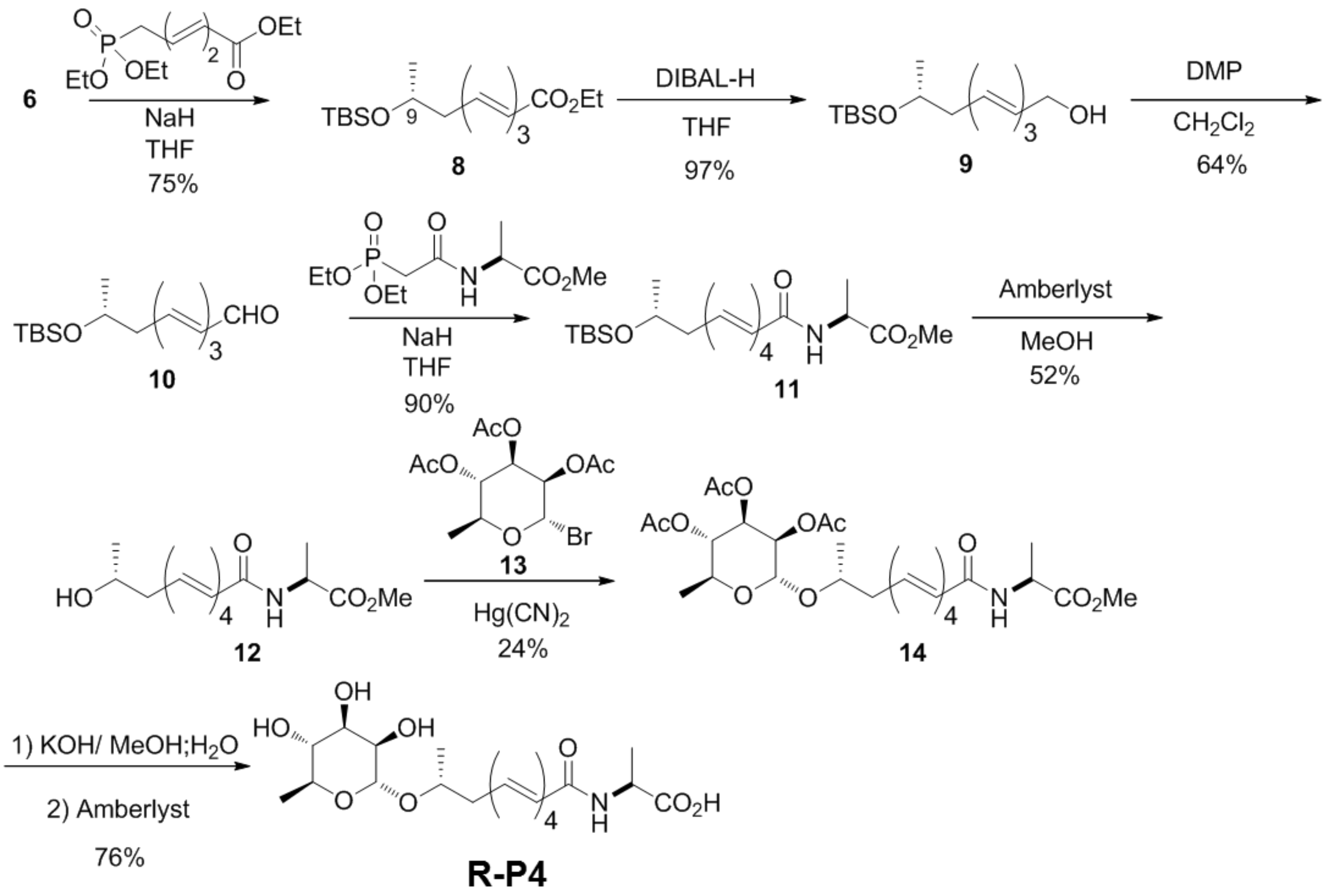

Scheme 3. Preparation of **R-P4.**

Synthesis of ester **(9*R*)-8**: According to GP-1, ester **(9*R*)-8** was synthesized from aldehyde **(3*R*)-6**, with a 75% yield. Colorless oil; $[\alpha]^{25}_{D}$ = + 2.6 (c = 0.01, $CHCl_3$). Its ^{1}H and ^{13}C NMR spectra matched with previously described[1,3].

Reduction of ester **(9*R*)-8** with DIBAL-H: According to GP-2, alcohol **(9*R*)-9** was prepared from ester **(9*R*)-8**, with a 97% yield (EtOAc/Hexane 1.5:8.5). Light yellow oil; $[\alpha]^{25}_{D}$ = +5.6 (c = 0.01, $CHCl_3$).

The mixture of isomers were separated by prep-HPLC ((EtOAc/Hexane 2:8)) and each isomer was isolated and characterized by ^{1}H- and ^{13}C-NMR. Signals were assigned using 2D-NMR spectra.

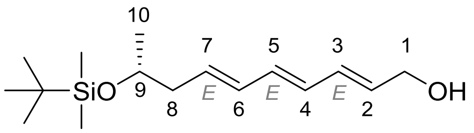

Major *E,E,E*-isomer, **^{1}H NMR (500 MHz, $CDCl_3$)** δ 6.27 (ddt, *J* = 15.2, 10.2, 1.4 Hz, 1H, 3-CH), 6.20 (dd, *J* = 14.8, 10.2 Hz, 1H, 4-CH), 6.13 (dd, *J* = 14.9, 10.4 Hz, 1H, 5-CH), 6.07 (ddt, *J* = 15.0, 10.2, 1.4 Hz, 1H, 6-CH), 5.82 (dt, *J* = 15.2, 6.0 Hz, 1H, 2-CH), 5.71 (dt, *J* = 15.0, 7.5 Hz, 1H, 7-CH), 4.19 (t, *J* = 5.2 Hz, 2H, 1-CH_2), 3.83 (h, *J* = 6.0 Hz, 1H, 9-CH), 2.29–2.15 (m, 2H, 8-CH_2), 1.13 (d, *J* = 6.1 Hz, 3H, 10-CH_3), 0.88 (s, 9H, $OSi(CH_3)_2C(CH_3)_3$), 0.04 (s, 3H, $OSi(CH_3)_2C(CH_3)_3$), 0.03 (s, 3H, $OSi(CH_3)_2C(CH_3)_3$). **^{13}C NMR (126 MHz, $CDCl_3$)** δ 133.7 (4-CH), 132.5 (7-CH), 132.3 (6-CH), 132.0 (3-CH), 131.4 (2-CH), 130.0 (5-CH), 68.7 (9-CH), 63.7 (1-CH_2), 43.3 (8-CH_2), 26.0 (CH_3, $OSi(CH_3)_2C(CH_3)_3$), 23.7 (10-CH_3), 18.3 (C, $OSi(CH_3)_2C(CH_3)_3$), –4.4 (CH_3, $OSi(CH_3)_2C(CH_3)_3$), –4.5 (CH_3, $OSi(CH_3)_2C(CH_3)_3$). **HRMS (ESI)**: $[M]^+$ calcd. $C_{16}H_{30}O_2Si$: 282.2015, found: 282.2026.

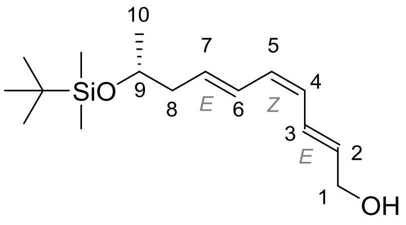

Minor *E,Z,E*-isomer, **^{1}H NMR (500 MHz, $CDCl_3$)** δ 6.71 (dd, *J* = 15.3, 10.6 Hz, 1H, 3-CH), 6.51 (dd, *J* = 15.1, 11.4 Hz, 1H, 6-CH), 5.98 (t, *J* = 10.9 Hz, 1H, 5-CH), 5.91 (t, *J* = 11.0 Hz, 1H, 4-CH), 5.84 (dt, *J* = 15.0, 5.9 Hz, 1H, 2-CH), 5.74 (dt, *J* = 15.0, 7.5 Hz, 1H, 7-CH), 4.24 (t, *J* = 5.2 Hz, 2H, 1-CH_2), 3.85 (h, *J* = 6.1 Hz, 1H, 9-CH), 2.37–2.19 (m, 2H, 8-CH_2), 1.14 (d, *J* = 6.1 Hz, 3H, 10-CH_3), 0.88 (s, 9H, $OSi(CH_3)_2C(CH_3)_3$), 0.04 (s, 3H, $OSi(CH_3)_2C(CH_3)_3$), 0.04 (s, 3H, $OSi(CH_3)_2C(CH_3)_3$). Carbon was assigned

from the HSQC (quaternary carbon is not shown): **^{13}C NMR (126 MHz, $CDCl_3$)**: δ 133.3 (7-CH), 132.5 (2-CH), 130.4 (5-CH), 127.6 (6-CH), 126.9 (3-CH), 126.8 (4-CH), 68.5 (9-CH), 63.5 (1-CH_2), 43.4 (8-CH_2), 25.9 (CH_3, $OSi(CH_3)_2C(\mathit{CH}_3)_3$), 23.6 (10-$CH_3$), –4.5 ($CH_3$, $OSi(\mathit{CH}_3)_2C(CH_3)_3$).

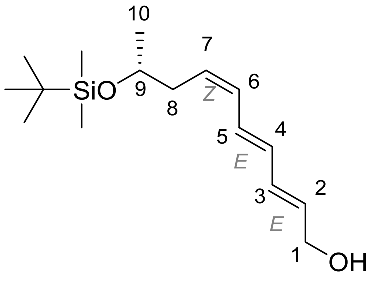

Minor *Z*,*E*,*E*-isomer **^{1}H NMR (500 MHz, $CDCl_3$)** δ 6.49 (dd, *J* = 14.8, 11.2 Hz, 1H, 5-CH), 6.31 (ddt, *J* = 15.1, 10.9, 1.8 Hz, 1H, 3-CH), 6.21 (dd, J = 15.0, 11.0 Hz, 1H, 4-CH), 6.09 (t, *J* = 10.3 Hz, 1H, 6-CH), 5.85 (dt, *J* = 15.3, 5.9 Hz, 1H, 2-CH), 5.51 (dt, *J* = 10.7, 5.9 Hz, 1H, 7-CH), 4.20 (t, *J* = 6.0 Hz, 2H, 1-CH_2), 3.85 (h, *J* = 6.0 Hz, 1H, 9-CH), 2.33 (m, 2H, 8-CH_2), 1.14 (d, *J* = 6.1 Hz, 3H, 10-CH_3), 0.88 (s, 9H, $OSi(CH_3)_2C(C\mathit{H}_3)_3$), 0.05 (s, 3H, $OSi(C\mathit{H}_3)_2C(CH_3)_3$), 0.04 (s, 3H, $OSi(C\mathit{H}_3)_2C(CH_3)_3$). Carbon was assigned from the HSQC (quaternary carbon is not shown): **^{13}C NMR (126 MHz, $CDCl_3$)**: δ 132.1 (4-CH), 132.0 (3-CH), 131.9 (2-CH), 129.9 (6-CH), 129.6 (7-CH), 129.2 (5-CH), 68.6 (9-CH), 63.5 (1-CH_2), 38.1 (8-CH_2), 26.0 (CH_3, $OSi(CH_3)_2C(\mathit{CH}_3)_3$), 23.6 (10-$CH_3$), –4.5 ($CH_3$, $OSi(\mathit{CH}_3)_2C(CH_3)_3$).

<u>Preparation of aldehyde **(9*R*)-10**</u>: According to GP-3, aldehyde **(9*R*)-10** was synthesized from alcohol **(9*R*)-9**, with a 64% yield (EtOAc/Hexane 0.5:9.5). Deep yellow oil; $[\alpha]^{25}_D$ = +9.1 (c = 0.01, $CHCl_3$); *E,E,E*-isomer: **^{1}H NMR (400 MHz, $CDCl_3$)** δ 9.55 (d, *J* = 7.9 Hz, 1H), 7.12 (dd, *J* = 15.3, 11.1 Hz, 1H), 6.65 (dd, *J* = 14.9, 10.6 Hz, 1H), 6.36 (dd, *J* = 14.9, 11.1 Hz, 1H), 6.24–6.10 (m, 2H), 6.03 (dt, *J* = 15.0, 7.4 Hz, 1H), 3.89 (h, *J* = 6.1 Hz, 1H), 2.32–2.26 (m, 2H), 1.15 (d, *J* = 6.1 Hz, 3H), 0.88 (s, 9H), 0.05 (s, 3H), 0.04 (s, 3H). **^{13}C NMR (101 MHz, $CDCl_3$)** δ 193.7 (CH), 152.4 (CH), 143.1 (CH), 139.0 (CH), 131.9 (CH), 131.0 (CH), 128.3 (CH), 68.4 (CH), 43.5 (CH_2), 26.0 (CH_3), 23.9 (CH_3), 18.3 (C), -4.3 (CH_3), -4.6 (CH_3). **HRMS (ESI):** $[M+Na]^+$ calcd. $C_{16}H_{28}O_2SiNa$: 303.1750, found: 303.1752.

<u>Synthesis of ester **(10*R*)-11**</u>: According to GP-1, ester **(*10R*)-11** was synthesized from aldehyde **(9*R*)-10** with a 90% yield (EtOAc/Hexane 2:8). The major isomer was isolated by prep-HPLC (EtOAc/Hexane 2:8). Light brown oil; $[\alpha]^{25}_D$ = +2.1 (c = 0.01, $CHCl_3$); *E,E,E,E isomer*: **^{1}H NMR (500 MHz, $CDCl_3$)** δ 7.27 (dd, *J* = 14.9, 11.3

Hz, 1H), 6.55 (dd, *J* = 14.8, 11.0 Hz, 1H), 6.36 (dd, *J* = 14.9, 10.7 Hz, 1H), 6.27 (dd, *J* = 14.8, 11.3 Hz, 1H), 6.19 (dd, *J* = 14.9, 11.0 Hz, 1H), 6.12 (dd, *J* = 15.1, 10.7 Hz, 1H), 6.02 (d, *J* = 7.5 Hz, 1H), 5.85 (d, *J* = 14.9 Hz, 1H), 5.83–5.78 (m, 1H), 4.70 (quint, *J* = 7.2 Hz, 1H), 3.85 (h, *J* = 6.0 Hz, 1H), 3.76 (s, 3H), 2.30–2.18 (m, 2H), 1.44 (d, *J* = 7.1 Hz, 3H), 1.13 (d, *J* = 6.1 Hz, 3H), 0.88 (s, 9H), 0.04 (s, 3H), 0.03 (s, 3H). **^{13}C NMR (126 MHz, $CDCl_3$)** δ 173.8 (C), 165.6 (C), 141.8 (CH), 140.4 (CH), 137.0 (CH), 134.7 (CH), 132.4 (CH), 130.3 (CH), 129.4 (CH), 122.3 (CH), 68.6 (CH), 52.7 (CH_3), 48.2 (CH), 43.5 (CH_2), 26.0 (CH_3), 23.8 (CH_3), 18.9 (CH_3), 18.3 (C), –4.4 (CH_3), –4.5 (CH_3). **HRMS (ESI):** $[M+Na]^+$ calcd. $C_{22}H_{37}NO_4SiNa$: 430.2384, found: 430.2399.

<u>Preparation of alcohol **(10*R*)-12**:</u> To a solution of ester **(*10R*)-11** (292 mg, 0.717 mmol) in MeOH (2 mL), amberlyst 15(H) was added (up to pH 5), and the mixture was stirred at room temperature for 90 min. Then, the residue was purified by flash chromatography (MeOH/CH_2Cl_2 1:9) to yield **(*10R*)-12** (110 mg, 52%). Light yellow oil; $[\alpha]^{25}_D$ = –5.3 (c = 0.09, $CHCl_3$); The major isomer (all-*E*) was isolated by prep-HPLC (EtOAc/CH_2Cl_2 7:3): **^{1}H NMR (500 MHz, $CDCl_3$)** δ 7.27 (dd, *J* = 14.9, 11.3 Hz, 1H), 6.54 (dd, *J* = 14.8, 11.0 Hz, 1H), 6.37 (dd, *J* = 15.0, 10.5 Hz, 1H), 6.29 (dd, *J* = 14.8, 11.4 Hz, 1H), 6.26–6.17 (m, 2H), 6.04 (d, *J* = 7.4 Hz, 1H), 5.87 (d, *J* = 15.1 Hz, 1H), 5.84–5.79 (m, 1H), 4.70 (quint, *J* = 7.2 Hz, 1H), 3.88 (h, *J* = 6.4 Hz, 1H), 3.76 (s, 3H), 2.36–2.22 (m, 2H), 1.44 (d, *J* = 7.2 Hz, 3H), 1.21 (d, *J* = 6.2 Hz, 3H). **^{13}C NMR (126 MHz, $CDCl_3$)** δ 173.8 (C), 165.6 (C), 141.7 (CH), 140.1 (CH), 136.4 (CH), 133.5 (CH), 133.1 (CH), 131.0 (CH), 129.9 (CH), 122.6 (CH), 67.5 (CH), 52.7 (CH_3), 48.3 (CH), 43.0 (CH_2), 23.1 (CH_3), 18.9 (CH_3). **HRMS (ESI):** $[M+Na]^+$ calcd. $C_{16}H_{23}NO_4Na$: 316.1519, found: 316.1507.

<u>Synthesis of rhamnose derivative **(10*R*)-14**</u>**:** To a solution of alcohol **12** (114 mg, 0.389 mmol) and $Hg(CN)_2$ (98 mg, 0.39 mmol) in dry CH_3CN (2 mL), bromorhamnose **13** (206 mg, 0.585 mmol) was added in three portions during 3 h, and the mixture was stirred for additional 4 h. Then, the solvent was removed. The residue was solved in AcOEt and washed with 1M solution of KBr, saturated solution of $NaHCO_3$ and water. The organic layer was dried (anhyd Na_2SO_4) and the solvent was removed. The residue was submitted to flash

chromatography (EtOAc/Hexane 3:7) to yield ester ***(10R)*-14** (52 mg, 24%). Brown oil; $[\alpha]^{25}_{D}$ -36.6 (c = 0.01, $CHCl_3$); *E,E,E,E* isomer: **^{1}H NMR (500 MHz, $CDCl_3$)** δ 7.26 (dd, *J* = 14.9, 11.3 Hz, 1H), 6.54 (dd, *J* = 14.4, 11.2 Hz, 1H), 6.40–6.14 (m, 4H), 6.05 (d, *J* = 7.4 Hz, 1H), 5.87 (d, *J* = 14.9 Hz, 1H), 5.79 (dt, *J* = 15.4, 7.6 Hz, 1H), 5.27 (dd, *J* = 10.2, 3.6 Hz, 1H), 5.17 (bs, 1H), 5.04 (t, *J* = 10.2 Hz, 1H), 4.83 (d, *J* = 1.8 Hz, 1H), 4.75–4.65 (quint, *J* = 7.2 Hz, 1H), 4.02–3.86 (m, 1H), 3.83–3.78 (m, 1H), 3.76 (s, 3H), 2.44–2.36 (m, 1H), 2.34–2.27 (m, 1H), 2.14 (s, 3H), 2.01 (s, 3H), 1.98 (s, 3H), 1.44 (d, *J* = 7.2 Hz, 3H), 1.17 (d, *J* = 6.3 Hz, 3H), 1.15 (d, *J* = 6.2 Hz, 3H). **^{13}C NMR (126 MHz, $CDCl_3$)** δ 173.7 (C), 170.2 (C), 170.0 (C), 165.4 (C), 141.5 (CH), 140.0 (CH), 136.3 (CH), 133.1 (CH), 132.9 (CH), 130.7 (CH), 129.6 (CH), 122.4 (CH), 95.1 (CH, $^1J_{C-H}$ = 170 Hz), 72.8 (CH), 71.1 (CH), 70.5 (CH), 69.2 (CH), 66.5 (CH), 52.5 (CH_3), 48.1 (CH), 40.4 (CH_2), 20.9 (CH_3), 20.8 (CH_3), 20.7 (CH_3), 18.9 (CH_3), 18.7 (CH_3), 17.3 (CH_3). **HRMS (ESI):** $[M+H]^+$ calcd $C_{28}H_{40}NO_{11}$: 566.2595, found: 566.2607.

Coupling constant values ($^3J_{H-H}$ and $^1J_{C-H}$) of the anomeric proton matched with those previously described for other α-rhamnopyranosyl derivatives [4-7].

<u>Preparation of **R-P4**:</u> To a solution of ester ***(10R)*-14** (27 mg, 0.05 mmol) in MeOH (1 mL), KOH 2M aqueous solution (0.29 mL, 0.57 mmol) was added and the mixture was stirred for 5 h. Then, washed amberlyst was added until pH 5, the mixture was submitted to flash chromatography (MeOH/CH_2Cl_2 1:1) to yield **R-P4** (15 mg, 76%). Light brown oil; $[\alpha]^{25}_{D}$ = –16.4 (c = 0.04, $CHCl_3$); Mixture of isomers. Data of major isomer are given: **^{1}H NMR (500 MHz, CD_3OD)** δ 7.18 (dd, *J* = 15.1, 11.0 Hz, 1H), 6.59 (dd, *J* = 14.8, 10.9 Hz, 1H), 6.44–6.32 (m, 2H), 6.27 (dd, *J* = 14.9, 10.9 Hz, 1H), 6.21 (dd, *J* = 15.2, 10.6 Hz, 1H), 6.07 (d, *J* = 14.8 Hz, 1H), 5.86 (dt, *J* = 14.8, 7.2 Hz, 1H), 4.34 (q, *J* = 7.6 Hz, 1H), 3.83 (m, 1H), 3.74 (bs, 1H), 3.68–3.61 (m, 2H), 3.37 (t, *J* = 9.5 Hz, 2H), 2.45–2.28 (m, 2H), 1.38 (d, *J* = 7.1 Hz, 3H), 1.23 (d, *J* = 6.4 Hz, 3H), 1.15 (d, *J* = 6.1 Hz, 3H). **^{13}C NMR (126 MHz, CD_3OD)** δ 179.4 (C), 168.0 (C), 141.7 (CH), 140.9 (CH), 137.6 (CH), 134.3 (CH), 134.0 (CH), 131.9 (CH), 131.1 (CH), 124.5 (CH), 99.3 (CH), 74.0 (CH), 73.4 (CH), 72.84 (CH), 72.4 (CH), 70.1 (CH), 51.6 (CH), 42.9 (CH_2), 19.19 (CH_3), 19.17 (CH_3), 17.9 (CH_3). **HRMS (ESI):** $[M+Na]^+$ calcd. $C_{21}H_{31}NO_8Na$: 448.1941, found: 448.1932. **Solubility**: MeOH, DMSO.

Synthesis of pP7X, pP7, and P7

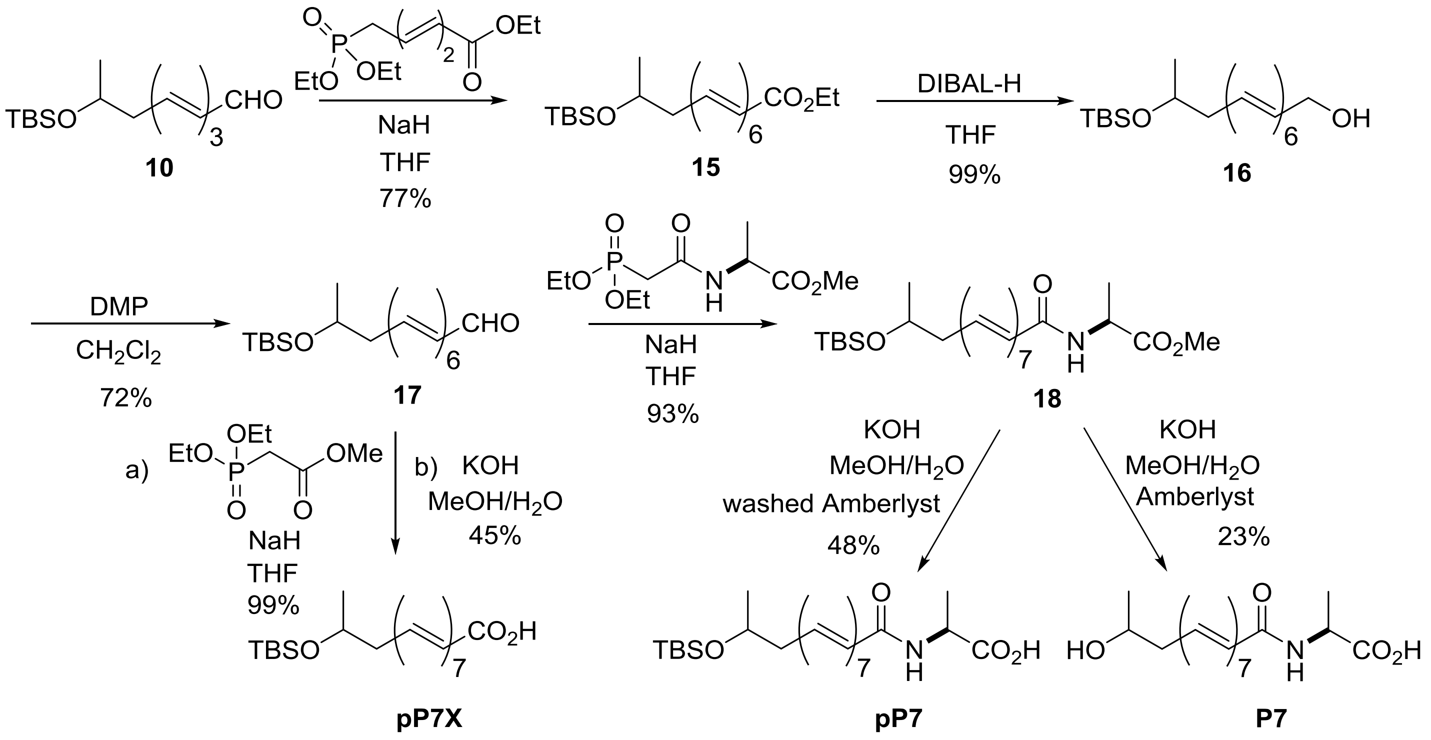

Scheme 4. Preparation of **pP7X**, **pP7** and **P7**.

Preparation of ester **15**: According to GP-1, polyene ester **15** was synthesized from racemic aldehyde **10**, with a 77% yield (EtOAc/Hexane 1:9). Yellow oil; *all-E* isomer: **^{1}H NMR (500 MHz, $CDCl_3$)** δ 7.31 (dd, *J* = 15.3, 11.0 Hz, 1H), 6.59 (dd, *J* = 14.8, 11.0 Hz, 1H), 6.44 (dd, *J* = 14.9, 10.7 Hz, 1H), 6.40–6.23 (m, 5H), 6.19 (dd, *J* = 14.7, 10.6 Hz, 1H), 6.18–6.05 (m, 1H), 5.85 (d, *J* = 15.2 Hz, 1H), 5.76 (dt, *J* = 15.1, 7.6 Hz, 1H), 4.20 (q, *J* = 7.1 Hz, 2H), 3.89–3.77 (m, 1H), 2.29–2.14 (m, 2H), 1.29 (t, *J* = 7.2 Hz, 3H), 1.12 (d, *J* = 6.2 Hz, 3H), 0.88 (s, 9H), 0.04 (s, 3H), 0.03 (s, 3H). **^{13}C NMR (126 MHz, $CDCl_3$)** δ 167.3 (C), 144.5 (CH), 140.9 (CH), 137.5 (CH), 136.0 (CH), 135.1 (CH), 133.4 (CH), 132.7 (CH), 132.0 (CH), 131.6 (CH), 131.0 (CH), 129.9 (CH), 120.5 (CH), 68.7 (CH), 60.4 (CH_2), 43.5 (CH_2), 26.0 (CH_3), 23.8 (CH_3), 18.3 (C), 14.5 (CH_3), –4.4 (CH_3), –4.6 (CH_3). **HRMS (ESI):** $[M+Na]^+$ calcd. $C_{24}H_{38}O_3SiNa$: 425.2482, found: 425.2486.

Reduction of ester **15** with DIBAL-H: According to GP-2, alcohol **16** was prepared from ester **15**, with a 99% yield (EtOAc/Hexane 3:7). Yellow oil; Mixture of isomers. Data of major isomer are given. **^{1}H NMR (400 MHz, $CDCl_3$)** δ 6.85–6.62 (m, 1H), 6.41–6.16 (m, 7H), 6.13–5.92 (m, 2H), 5.94–5.80 (m, 1H), 5.79–5.59 (m, 1H), 4.27–4.18 (bs, 2H), 3.93–3.79 (m, 1H), 2.33–2.17 (m, 2H), 1.15 (d, *J* = 6.2 Hz, 3H), 0.90 (s, 9H), 0.06 (s, 3H), 0.06 (s, 3H).**^{13}C NMR (101 MHz, $CDCl_3$)** δ 133.8 (CH), 133.7 (CH), 133.6 (CH), 133.5 (CH), 132.7 (CH), 132.5 (CH), 132.4 (CH), 132.3 (CH), 132.2 (CH), 131.9 (CH), 131.7 (CH), 131.2 (CH), 68.6 (CH), 63.4 (CH_2), 43.3 (CH_2), 25.9 (CH_3), 23.6 (C), 18.2 (CH_3), –4.5 (CH_3), –4.7 (CH_3). Due to the instability of this compound, a good mass spectrum could not be obtained.

Synthesis of aldehyde **17**: According to GP-3, aldehyde **17** was synthesized from alcohol **16**, with a 72% yield (EtOAc/Hexane 0.5:9.5). Yellow oil; *all-E* isomer: **^{1}H NMR (500 MHz, $CDCl_3$)** δ 9.56 (d, *J* = 8.0, 1H), 7.14 (dd, *J* = 15.1, 11.3 Hz, 1H), 6.71 (dd, *J* = 14.7, 11.2 Hz, 1H), 6.52 (dd, *J* = 14.7, 11.0 Hz, 1H), 6.50–6.39 (m, 2H), 6.38–6.35 (m, 1H), 6.34–6.26 (m, 2H), 6.21 (dd, *J* = 14.9, 10.8 Hz, 1H), 6.17–6.10 (m, 2H), 5.79 (dt, *J* = 15.1, 7.6 Hz, 1H), 3.85 (dt, *J* = 12.0, 5.9 Hz, 1H), 2.31–2.21 (m, 2H), 1.13 (d, *J* = 6.1 Hz, 3H), 0.88 (s, 9H), 0.04 (s, 3H), 0.03 (s, 3H). **^{13}C NMR (126 MHz, $CDCl_3$)** δ 193.6 (CH), 152.0 (CH), 143.0 (CH), 139.2 (CH), 137.1 (CH), 135.8 (CH), 134.0 (CH), 132.7 (CH), 131.8 (CH), 131.3 (CH), 130.87 (CH), 129.7 (CH), 68.7 (CH), 43.5 (CH_2), 26.0 (CH_3), 23.8 (CH_3), 18.3 (C), –4.4 (CH_3), –4.6 (CH_3). **HRMS (ESI):** $[M+Na]^+$ calcd. $C_{22}H_{34}O_2SiNa$: 381.2220, found 381.2229.

Preparation of derivative **pP7X**: According to GP-1, a polyene ester precursor was synthesized from aldehyde **17**, with a 99% yield (EtOAc/Hexane 0.5:9.5). Deep yellow oil; *all-E* isomer isolated by prep-HPLC (EtOAc/Hexane 1:9): **^{1}H NMR (500 MHz, $CDCl_3$)** δ 7.35 (dd, *J* = 15.3, 11.5 Hz, 1H), 6.63 (dd, *J* = 14.8, 11.2 Hz, 1H), 6.48 (dd, *J* = 14.7, 11.1 Hz, 1H), 6.43–6.21 (m, 8H), 6.14 (dd, *J* = 15.0, 10.3 Hz, 1H), 5.89 (d, *J* = 15.2 Hz, 1H), 5.77 (dt, *J* = 15.1, 7.6 Hz, 1H), 3.86 (h, *J* = 6.3 Hz, 1H), 3.77 (s, 3H), 2.3–2.22 (m, 2H), 1.15 (d, *J* = 6.0 Hz, 3H), 0.91 (s, 9H), 0.07 (s, 3H), 0.06 (s, 3H). **^{13}C NMR (126 MHz, $CDCl_3$)** δ 167.6 (C), 144.7 (CH), 141.0

(CH), 137.6 (CH), 136.0 (CH), 135.1 (CH), 134.5 (CH), 133.0 (CH), 132.6 (CH), 132.2 (CH), 132.2 (CH), 131.7 (CH), 131.1 (CH), 129.8 (CH), 119.9 (CH), 68.6 (CH), 51.5 (CH_3), 43.3 (CH_2), 25.9 (CH_3), 23.6 (CH_3), 18.2 (C), –4.5 (CH_3), –4.7 (CH_3). **HRMS (ESI):** $[M+H]^+$ calcd. $C_{25}H_{39}O_3Si$: 415.2663, found 415.2671. According to **GP-4** (using 24 mL of THF), this ester intermediate was transformed into **pP7X**, with a 45% yield. Preparative TLC ($MeOH/CH_2Cl_2$ 1:9). Prep-HPLC (EtOAc/Hexane 3:7). Light yellow oil; **^{1}H NMR (500 MHz, $CDCl_3$)** δ 7.40 (dd, *J* = 15.1, 11.4 Hz, 1H), 6.65 (t, *J* = 13.1 Hz, 1H), 6.56–6.19 (m, 9H), 6.11 (t, *J* = 11.7 Hz, 1H), 5.87 (d, *J* = 15.0 Hz, 1H), 3.89–3.81 (m, 1H), 2.35–2.18 (m, 2H), 1.15 (d, *J* = 6.1 Hz, 3H), 0.88 (s, 9H), 0.04 (s, 3H), 0.04 (s, 3H). **^{13}C NMR (126 MHz, $CDCl_3$)** δ 170.5 (C), 146.6 (CH), 141.9 (CH), 138.2 (CH), 136.2 (CH), 135.3 (CH), 133.1 (CH), 132.8 (CH), 132.6 (CH), 132.1 (CH), 131.7 (CH), 131.0 (CH), 130.2 (CH), 129.5 (CH), 118.9 (CH), 68.6 (CH), 43.3 (CH_2), 25.9 (CH_3), 23.6 (CH_3), 18.1 (C), –4.6 (CH_3), –4.7 (CH_3); **HRMS (ESI):** $[M–H]^+$ calcd. $C_{24}H_{35}O_3Si$: 399.2355, found 399.2361. **Solubility**: CH_2Cl_2, $CHCl_3$ and DMSO among others.

Synthesis of ester **18**: According to GP-1, polyene ester **18** was synthesized from aldehyde **17**, with a 93% yield (EtOAc/Hexane 3:7). Prep-HPLC (EtOAc/Hexane 1:1). Light orange oil; **^{1}H NMR (500 MHz, $CDCl_3$)** δ 7.31 (dd, *J* = 15.3, 11.5 Hz, 1H), 6.69–6.52 (m, 2H), 6.50–6.39 (m, 2H), 6.39–6.27 (m, 6H), 6.13–6.08 (m, 1H), 6.01 (dd, *J* = 16.3, 10.9 Hz, 1H), 5.90 (d, *J* = 14.9 Hz, 1H), 4.72 (quint, *J* = 7.2 Hz, 1H), 3.93–3.83 (m, 1H), 3.79 (s, 3H), 2.44–2.23 (m, 2H), 1.47 (d, *J* = 7.1 Hz, 3H), 1.16 (d, *J* = 6.1 Hz, 3H), 0.90 (s, 9H), 0.07 (s, 3H), 0.06 (s, 3H). **^{13}C NMR (126 MHz, $CDCl_3$)** 173.7 (C), 165.5 (C), 141.5 (CH), 140.1 (CH), 136.8 (CH), 135.5 (CH), 133.7 (CH), 133.0 (CH), 132.8 (CH), 132.1 (CH), 130.9 (CH), 130.0 (CH), 129.9 (CH), 127.9 (CH), 127.7 (CH), 122.5 (CH), 68.5 (CH), 52.5 (CH_3), 48.1 (CH), 43.4 (CH_2), 25.9 (CH_3), 23.7 (CH_3), 18.7 (CH_3), 18.2 (C), –4.5 (CH_3), –4.7 (CH_3). **HRMS (ESI):** $[M+Na]^+$ calcd. $C_{28}H_{43}NO_4SiNa$: 508.2853, found 508.2869.

Preparation of **pP7**: According to GP-4 (using 12 mL of THF), **pP7** was synthesized from **18**, with a 48% yield ($MeOH/CH_2Cl_2$ 3:7). Prep-HPLC (EtOAc/Hexane 8:2). Deep orange oil. **^{1}H NMR (500 MHz, CD_3OD)** δ 7.24 (dd, *J* = 15.0, 11.4 Hz, 1H), 6.67 (dd, *J* = 14.7, 10.8 Hz, 1H), 6.52 (dd, J = 14.8, 10.3 Hz, 1H), 6.47–6.34 (m, 7H),

6.31–6.22 (m, 1H), 6.19–6.12 (m, 1H), 6.09 (d, *J* = 14.9 Hz, 1H), 5.77 (dt, *J* = 15.2, 7.6 Hz, 1H), 4.49 (bs, 1H), 4.00–3.86 (m, 1H), 2.25 (dd, *J* = 7.7, 5.8 Hz, 2H), 1.44 (d, *J* = 7.1 Hz, 3H), 1.15 (d, *J* = 6.0 Hz, 3H), 0.91 (s, 9H), 0.08 (s, 3H), 0.07 (s, 3H). **^{13}C NMR (126 MHz, CD_3OD)** δ 174.7 (C), 167.2 (C), 140.96 (CH), 140.94 (CH), 140.0 (CH), 136.8 (CH), 135.4 (CH), 134.5 (CH), 133.9 (CH), 132.8 (CH), 132.33 (CH), 132.28 (CH), 132.0 (CH), 131.8 (CH), 131.2 (CH), 129.9 (CH), 68.61 (CH), 42.93 (CH_2), 24.95 (CH_3) , 22.66 (CH_3), 17.55 (C), 16.40 (CH_3), -5.77 (CH_3), -5.91 (CH_3).One CH was not observed. **HRMS (ESI):** $[M+Na]^+$ calcd. $C_{27}H_{41}O_4NSiNa$: 494.2697, found 494.2706. **Solubility**: MeOH, DMSO.

<u>Synthesis of **P7**:</u> According to GP-4 (using 12 mL of THF, and adding non-washed Amberlyst), **P7** was synthesized from **18**, with a 23% yield. . Preparative TLC (EtOAc/Hexane 8:2). Yellow oil. **^{1}H NMR (500 MHz, CD_3OD)** δ 7.24 (dd, *J* = 14.7, 11.4 Hz, 1H), 6.67 (dd, *J* = 14.7, 11.1 Hz, 1H), 6.58–6.25 (m, 10H), 6.18 (t, *J* = 11.2 Hz, 1H), 6.08 (d, *J* = 14.9 Hz, 1H), 4.54–4.42 (m, 1H), 3.87–3.77 (m, 1H), 2.46–2.22 (m, 2H), 1.44 (d, *J* = 7.3 Hz, 3H), 1.19 (d, *J* = 6.2 Hz, 3H). **HRMS (ESI):** $[M–H]^+$ calcd. $C_{21}H_{26}O_4N$: 356.1867, found 356.1866. Owing to the unusual physical properties of this kind of compounds in terms of solubility, a clear ^{13}C NMR could not be obtained even using long acquisition times. HSQC 2D-NMR experiment has been carried out and attached to the ESI. **Solubility**: MeOH (partially), DMSO (0.02 M, fully soluble).

Synthesis of pP9 and P9

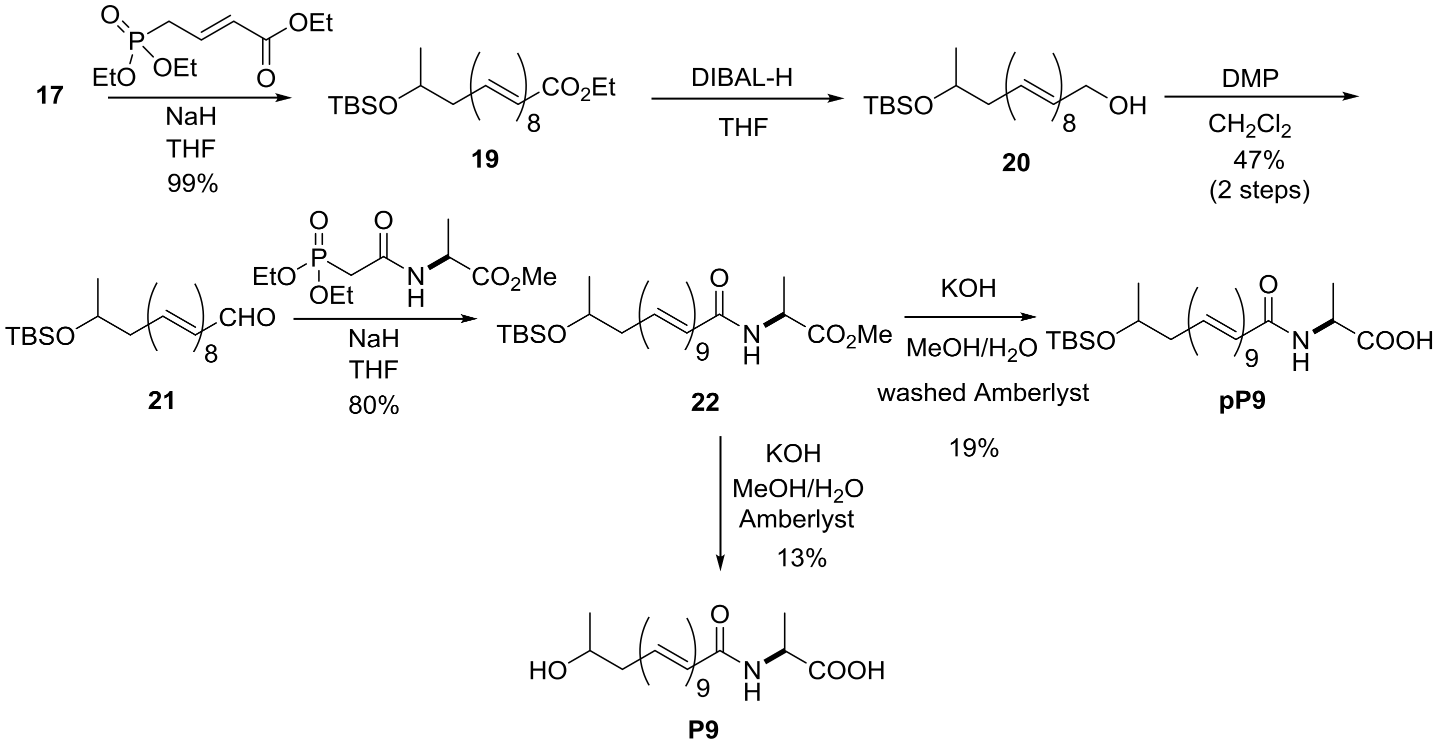

Scheme 5. Synthesis of **pP9** and **P9**.

Synthesis of ester **19**: According to GP-1, polyene ester **19** was synthesized from aldehyde **17**, with a 99% yield (EtOAc/Hexane 1:9). Deep orange oil; Mixture of isomers. Data of major isomer are given **^{1}H NMR (500 MHz, $CDCl_3$)** δ 7.35 (dd, *J* = 15.4, 11.5 Hz, 1H), 6.63 (dd, *J* = 14.7, 11.1 Hz, 1H), 6.53–6.44 (m, 1H), 6.44–6.20 (m, 10H), 6.13 (dd, *J* = 15.0, 10.1 Hz, 1H), 5.89 (dd, *J* = 15.2, 3.2 Hz, 1H), 5.76 (dt, *J* = 15.0, 7.5 Hz, 1H), 4.23 (q, *J* = 7.1 Hz, 2H), 3.86 (h, *J* = 6.3 Hz, 1H), 2.33–2.18 (m, 2H), 1.32 (t, *J* = 7.1 Hz, 3H), 1.15 (d, *J* = 6.1 Hz, 3H), 0.90 (s, 9H), 0.07 (s, 3H), 0.06 (s, 3H). **^{13}C NMR (125 MHz, $CDCl_3$)** δ 167.6 (C), 144.7 (CH), 141.0 (CH), 137.6 (CH), 136.0 (CH), 135.1 (CH), 134.6 (CH), 134.2 (CH), 132.8 (CH), 132.7 (CH), 132.5 (CH), 132.4 (CH), 131.8 (CH), 131.2 (CH), 129.9 (CH), 120.0 (CH), 68.6 (CH), 60.3 (CH_2), 51.5 (CH_3), 43.4 (CH_2), 25.9 (CH_3), 18.2 (C), 14.3 (CH_3), –4.5 (CH_3), –4.7 (CH_3). **HRMS (ESI)** m/z calcd for $C_{28}H_{42}O_3SiNa$ $[M+Na]^+$ 477.2795, found 477.2790.

Reduction of ester **19** with DIBAL-H: According to GP-2, alcohol **20** was prepared from ester **19**. This compound is highly unstable, and it was immediately used in the next step.

Synthesis of aldehyde **21**: According to GP-3, aldehyde **21** was synthesized from alcohol **20**, with a 47% yield (2 steps) (EtOAc/Hexane 1:9). Orange-red oil; Data of the major isomer (*all-E*-**21**) is given: **^{1}H NMR (500 MHz, $CDCl_3$)** δ 9.58 (d, *J* = 8.0 Hz, 1H), 7.16 (dd, *J* = 15.1, 11.3 Hz, 1H), 6.74 (dd, *J* = 14.7, 11.3 Hz, 1H), 6.56 (dd, *J* = 14.7, 11.1 Hz, 1H), 6.50–6.09 (m, 12H), 5.77 (dt, *J* = 15.1, 7.6 Hz, 1H), 3.86 (h, *J* = 6.1 Hz, 1H), 2.33–2.20 (m, 2H), 1.15 (d, *J* = 6.1 Hz, 3H), 0.90 (s, 9H), 0.07 (s, 3H), 0.06 (s, 3H).**^{13}C NMR (126 MHz, $CDCl_3$)** δ 193.5 (C), 151.9 (CH), 142.9 (CH), 139.2 (CH), 137.1 (CH), 135.9 (CH),135.1 (CH), 134.6 (CH), 133.2 (CH), 132.8 (CH), 132.5 (CH), 132.4 (CH), 132.3 (CH), 131.6 (CH), 131.2 (CH), 130.9 (CH), 129.9 (CH), 68.7 (CH), 43.5 (CH_2), 26.0 (CH_3), 23.8 (CH_3), 18.3 (C), –4.4 (CH_3), –4.5 (CH_3). **HRMS (EI):** $[M]^+$ calcd. $C_{26}H_{38}O_2Si$: 410.2641, found 410.2648.

Preparation of amide **22**: According to GP-1, polyene amide **22** was synthesized from aldehyde **21**, with an 80% yield (EtOAc/Hexane 3.5:6.5). Orange-red oil; *all-E* isomer: **^{1}H NMR (500 MHz, $CDCl_3$)** δ 7.28 (dd, *J* = 14.9, 11.5 Hz, 1H), 6.63–6.50 (m, 2H), 6.50–6.16 (m, 10H), 6.16–6.05 (m, 3H), 5.88 (d, *J* = 14.9 Hz, 1H), 5.81–5.67 (m, 1H), 4.70 (quint, *J* = 7.2 Hz, 1H), 3.91–3.79 (m, 1H), 3.76 (s, 3H), 2.34–2.14 (m, 2H), 1.44 (d, *J* = 7.1 Hz, 3H), 1.14 (d, *J* = 6.2 Hz, 3H), 0.88 (s, 9H), 0.05 (s, 3H), 0.04 (s, 3H). **^{13}C NMR (101 MHz, $CDCl_3$)** δ 173.7 (C), 165.4 (C), 141.5 (CH), 140.1 (CH), 136.9 (CH), 135.6 (CH), 134.8 (CH), 134.3 (CH), 134.1 (CH), 133.9 (CH), 133.0 (CH), 132.8 (CH), 132.7 (CH), 132.5 (CH), 132.1 (CH), 131.2 (CH), 130.0 (CH), 129.2 (CH), 127.9 (CH), 122.5 (CH), 68.6 (CH), 52.5 (CH_3), 48.1 (CH), 43.3 (CH_2), 25.9 (CH_3), 23.7 (CH_3), 18.7 (CH_3). 18.1 (C), -4.5 (CH_3), -4.7 (CH_3); HRMS (APCI) m/z calcd for $C_{32}H_{48}NO_4Si$ $[M+H]^+$ 538.3347, found 538.3351.

Saponification of amide **22** with KOH/MeOH: According to GP-4 (using 12 mL of THF), **pP9** was synthesized from **22**, with a 19% yield (MeOH/CH_2Cl_2 4:6). Orange-red oil; **^{1}H NMR (500 MHz, DMSO-*d*$_6$+0.1% TFA-*d*)** δ 8.34 (d, *J* = 7.5 Hz, 1H), 7.09 (dd, *J* = 14.9, 11.3 Hz, 1H), 6.75–6.64 (m, 1H), 6.61–6.22 (m, 14H), 6.18–6.06 (m, 2H), 4.31–4.23 (m, 1H), 3.91–3.81 (m, 1H), 2.25–2.12 (m, 2H), 1.28 (d, *J* = 7.4 Hz, 6H), 0.83 (s, 9H), –0.05 (s, 6H). **HRMS (ESI):** $[M–H]^+$ calcd. $C_{31}H_{44}NO_4Si$: 522.3045, found 522.3050. Owing to the unusual physical properties of this kind of compounds in terms of solubility, a clear ^{13}C NMR could not be obtained even using long acquisition times. **Solubility**: DMSO +0.1% TFA (0.02 M, fully soluble)

Synthesis of **P9**: According to GP-4 (using 12 mL of THF, and adding non-washed Amberlyst), **P9** was synthesized from **22**, with a 13% yield (MeOH/CH_2Cl_2 7:3). Deep orange oil; **^{1}H NMR (600 MHz, CD_3OD)** δ 7.32–7.08 (m, 1H), 6.91–5.89 (m, 16H), 5.82–5.69 (m, 1H), 4.41–4.28 (m, 1H), 2.39–2.11 (m, 2H), 1.25 (bs, 3H), 0.85 (bs, 3H); Owing to the unusual physical properties of this kind of compound in terms of solubility, a clear ^{13}C NMR could not be obtained in any solvent even using long acquisition times. **HRMS (ESI):** $[M–H]^+$ calcd. $C_{25}H_{30}NO_4$: 408.2180, found 408.2198. **Solubility**: DMSO +0.1% TFA (0.02 M)

General protocol for the solubilization of compounds. 0.02 M solutions were prepared using DMSO (for **pP1, R-P4, pP7x, pP7** and **P7**) or DMSO + 0.1% TFA (for **pP9** and **P9**) as solvent. Generally 0.04 mmol of compound were dissolved in 2 mL of solvent. At this concentration, clear solutions were obtained in every case. Solutions were stored at –20 °C, under Ar atmosphere and protected from light.

Supplementary References

1 Sivasubramanian, K., Kaanumalle, L. S., Uppili, S. & Ramamurthy, V. Value of zeolites in asymmetric induction during photocyclization of pyridones, cyclohexadienones and naphthalenones. *Organic & biomolecular chemistry* **5**, 1569-1576, doi:10.1039/b702572f (2007).

2 Fortunati, T., D'Acunto, M., Caruso, T. & Spinella, A. Chemoenzymatic preparation of musky macrolactones. *Tetrahedron* **71**, 2357-2362, doi:10.1016/j.tet.2015.03.007 (2015).

3 Duplantier, A. J. & Masamune, S. Pimaricin - Stereochemistry and Synthesis of Its Aglycon (Pimarolide) Methyl-Ester. *J Am Chem Soc* **112**, 7079-7081, doi:DOI 10.1021/ja00175a063 (1990).

4 Wang, G., Huang, Z. & Negishi, E. Highly stereoselective and efficient synthesis of ω-heterofunctional di- and trienoic esters for Horner–Wadsworth–Emmons reaction via alkyne hydrozirconation and Pd-catalyzed alkenylation. *Tetrahedron Letters* **50**, 3220-3223, doi:10.1016/j.tetlet.2009.02.023 (2009).

5 Nishizawa, M. *et al.* Synthesis and glycosylation shift of 1,1'-disaccharides. *Chem Pharm Bull (Tokyo)* **42**, 982-984, doi:10.1248/cpb.42.982 (1994).

6 de Bruyn, A., Anteunis, M., de Gussem, R. & Dutton, G. G. S. 1H-N.m.r. study of l-rhamnose, methyl α-l-rhamnopyranoside, and 4- O-β- d-galactopyranosyl- l-rhamnose in deuterium oxide. *Carbohydrate Research* **47**, 158-163 (1976).

7 Bebault, G. M., G.G.S., D. & Warfield, C. K. Synthesis of 4-O-α-l-rhamnopyranosyl-l-rhamnopyranose. *Carbohydrate Research* **34**, 174-179 (1974).
