# Supplementary material for: Lipid analogs reveal features critical for hemolysis and diminish granadaene mediated Group B Streptococcus infection
Source: Nat Commun. 2020 Mar 20;11:1502. doi: 10.1038/s41467-020-15282-0 (PMC7083881; doi:10.1038/s41467-020-15282-0)
Supplement: Supplementary file 2 — Reporting Summary [file 41467_2020_15282_MOESM2_ESM.pdf]

nature research

Corresponding author(s): Lakshmi Rajagopal

Last updated by author(s): February 13, 2020

Reporting Summary

Nature Research wishes to improve the reproducibility of the work that we publish. This form provides structure for consistency and transparency in reporting. For further information on Nature Research policies, see Authors & Referees and the Editorial Policy Checklist .

Statistics

For all statistical analyses, confirm that the following items are present in the figure legend, table legend, main text, or Methods section.

n/a	Confirmed	
☐	☒	The exact sample size (n) for each experimental group/condition, given as a discrete number and unit of measurement
☒	☐	A statement on whether measurements were taken from distinct samples or whether the same sample was measured repeatedly
☐	☒	The statistical test(s) used AND whether they are one- or two-sided<br>*Only common tests should be described solely by name; describe more complex techniques in the Methods section.*
☒	☐	A description of all covariates tested
☐	☒	A description of any assumptions or corrections, such as tests of normality and adjustment for multiple comparisons
☐	☒	A full description of the statistical parameters including central tendency (e.g. means) or other basic estimates (e.g. regression coefficient) AND variation (e.g. standard deviation) or associated estimates of uncertainty (e.g. confidence intervals)
☐	☒	For null hypothesis testing, the test statistic (e.g. F, t, r) with confidence intervals, effect sizes, degrees of freedom and P value noted<br>*Give P values as exact values whenever suitable.*
☒	☐	For Bayesian analysis, information on the choice of priors and Markov chain Monte Carlo settings
☒	☐	For hierarchical and complex designs, identification of the appropriate level for tests and full reporting of outcomes
☒	☐	Estimates of effect sizes (e.g. Cohen's d, Pearson's r), indicating how they were calculated

Our web collection on statistics for biologists contains articles on many of the points above.

Software and code

Policy information about availability of computer code

Data collection	Flow cytometry data were collected using FACS Diva Software version 8.0.1 (BD Biosciences). Scans of spot blots were obtained using ImageStudio version 5.2 (LI-COR). LabSolutions software version 5.8.4 (Shimadzu) was used during HPLC purification of granadaene. Mnova NMR software was used during NMR data collection, and JEOL Acquisition System software was used to capture SEM images.
Data analysis	GraphPad Prism version 7.03 was used to compute all statistical tests. Flow cytometry data were analyzed using FlowJo version 10.1 (FlowJo). ImageJ version 1.52a (National Institutes of Health, USA) was used to quantify signal intensity of spot blots. All compounds were drawn in ChemDraw Ultra software version 12 (Perkin Elmer). Structures underwent energy minimization in Chem3D software (ChemOffice Professional, Perkin Elmer) and were visualized in Mercury software 2019 (The Cambridge Crystallographic Data Centre), where the polyene chain lengths were estimated. Photographs of blood agar plates were processed using Photoshop CC version 19.0 (Adobe). Mnova NMR version 12.0.4 (Mestrelab) software was used to visualize and analyze NMR data.

For manuscripts utilizing custom algorithms or software that are central to the research but not yet described in published literature, software must be made available to editors/reviewers. We strongly encourage code deposition in a community repository (e.g. GitHub). See the Nature Research guidelines for submitting code & software for further information.

Data

Policy information about availability of data

All manuscripts must include a data availability statement. This statement should provide the following information, where applicable:

- Accession codes, unique identifiers, or web links for publicly available datasets
- A list of figures that have associated raw data
- A description of any restrictions on data availability

The source data underlying Fig. 1b, c, d, e; Fig. 3a, d; Fig. 4a, b, c; Fig. 5b, c, d; and Supplementary Fig. 3 b, c and Supplementary Fig 4 are provided as a Source Data file. All other relevant data supporting the key findings of this study are available within the article and its Supplementary Information file or from the corresponding authors upon request.

Field-specific reporting

Please select the one below that is the best fit for your research. If you are not sure, read the appropriate sections before making your selection.

☒ Life sciences ☐ Behavioural & social sciences ☐ Ecological, evolutionary & environmental sciences

For a reference copy of the document with all sections, see nature.com/documents/nr-reporting-summary-flat.pdf

Life sciences study design

All studies must disclose on these points even when the disclosure is negative.

Sample size	Sample sizes for animal experiments were estimated based on means and standard deviations observed in preliminary studies (alpha level = 0.05 with 80% power). For all in vitro studies, a sample size of n = 3 (i.e. performed three independent times in technical triplicate) was predefined during the formulation of hypotheses. This was based on preliminary studies (alpha level = 0.05 with 80% power).
Data exclusions	No data exclusions
Replication	All in vitro experiments were repeated three times in technical triplicate, unless otherwise noted. In addition, in vitro replicates involving human blood or human cells were performed with different donors on different days to ensure reproducibility of the findings. Three replications of SEM experiments (Fig. c, f), red blood agar spotting, and PI/AV experiments were consistent, and representative data are shown in the manuscript. Otherwise, all replicates for in vitro studies are depicted in the manuscript. Attempts at reproducing in vivo have been successful.
Randomization	Animals were randomly assigned to experimental groups. For experiments involving human blood or human cells, children (under 18 years), pregnant adults, and non-healthy adults (e.g. immunocompromized, showing cold/flu symptoms, etc) were excluded per our IRB protocol since they can exhibit susceptibilities. Different adult human donors were used for replicates of each experiment involving human blood or human cells to control for variability among donors.
Blinding	Investigators were blinded during data collection.

Reporting for specific materials, systems and methods

We require information from authors about some types of materials, experimental systems and methods used in many studies. Here, indicate whether each material, system or method listed is relevant to your study. If you are not sure if a list item applies to your research, read the appropriate section before selecting a response.

Materials & experimental systems

n/a	Involved in the study	
☐	☒	Antibodies
☒	☐	Eukaryotic cell lines
☒	☐	Palaeontology
☐	☒	Animals and other organisms
☐	☒	Human research participants
☒	☐	Clinical data

Methods

n/a	Involved in the study	
☒	☐	ChIP-seq
☐	☒	Flow cytometry
☒	☐	MRI-based neuroimaging

Antibodies

Antibodies used	CD19-PerCP/Cy5.5 (clone HIB19, BioLegend cat# 302230, lot# B217065), CD3-epsilon-FITC (clone SP34, BD cat# 556611, lot# 7011528), CD4-V450 (clone L200, BD cat # 560811, lot# 9108984), CD8-PerCP/Cy5.5 (clone SK1, BioLegend cat# 344710, lot# B217065), CD69-PE/Cy7 (clone FN50, cat# 557745, lot# 9031836), Alexa Fluor 680 goat anti-mouse IgG (H+L) (Invitrogen cat# A21057, lot# 927077), anti-human CD3-epsilon (clone SP34, BD cat# 556610, lot# 6138553), anti-human CD40 (clone mAb 89, Enzo cat# ENZ-ABS148-0100, lot# 03231809)
Validation	CD19-PerCP/Cy5.5 (from manufacturer website): Each lot of this antibody is quality control tested by immunofluorescent taining with flow cytometric analysis.<br>CD3-epsiolon-FITC (from manufacturer website): Clone SP34 reacts with human form of the 20 kDa epsilon subunit of the CD3 antigen/T-cell receptor (TCR) complex...This antibody is routinely tested by flow cytometric analysis.<br>CD4-V450 (from manufacturer website): The L200 monoclonal antibody specifically binds to the human form of the 56 kDa transmembrane glycoprotein, CD4, which is present on the T-helper/inducer subset of normal human donor peripheral blood lymphocytes. The antibody is conjugated to BD Horizon™ V450, which has been developed for use in multicolor flow cytometry experiments and is available exclusively from BD Biosciences.<br>CD8-PerCP/Cy5.5 (from manufacturer website): Each lot of this antibody is quality control tested by immunofluorescent staining with flow cytometric analysis.

CD69-PE/Cy7 (from manufacturer website): The FN50 monoclonal antibody specifically binds to human CD69. CD69 is also known as activation-induced molecule (AIM), early activation antigen (EA-1), very early activation antigen (VEA), C-type lectin domain family 2 member C (CLEC2C), MLR-3, GP32/28 and Leu-23...Upon activation, CD69 antigen expression increases on lymphocytes. Antibody has undergone human reactivity QC testing by manufacturer.

Alexa Fluor 680 goat anti-mouse IgG: References for Western blot application are as follows: Vanaveski, T, et al. (2017). Frontiers in Neuroscience, 11(38); Vieweg, S., et al. (2016). J Biol Chem, 291(23); Capitanio, J.S., Montpetit, B., and Wozniak, R.W. (2017). eLife, doi: 10.7554/eLife.18825.

anti-human CD3-epsilon: References for T cell activation include: Dumont, et al. (1998). Journal of Immunology, 160; Bjorndahl, et al. (1989). European Journal of Immunology, 19. Also confirmed by data in the manuscript.

anti-human CD40: Reference for B cell stimulation: Van Belle, et al. (2016). Journal of Immunology Research, doi:10.1155/2016/5281823

Animals and other organisms

Policy information about studies involving animals; ARRIVE guidelines recommended for reporting animal research

Laboratory animals	Mice, (Mus musculus), C57BL6/J male and female, 6-12 weeks age
Wild animals	No wild animals were used in the study.
Field-collected samples	No field-collected samples were used in the study.
Ethics oversight	IACUC, Seattle Childrens Research Institute

Note that full information on the approval of the study protocol must also be provided in the manuscript.

Human research participants

Policy information about studies involving human research participants

Population characteristics	Adult humans 18yrs and older and include males or females. Not pregnant and not immunocompromised.
Recruitment	Email and verbal. All donors were residents of the United States. We do not expect that this would introduce a bias on susceptibility to GBS factors given that multiple countries have report adverse outcomes due to GBS infections.
Ethics oversight	IRB, Seattle Childrens Research Institute

Note that full information on the approval of the study protocol must also be provided in the manuscript.

Flow Cytometry

Plots

Confirm that:

- [x] The axis labels state the marker and fluorochrome used (e.g. CD4-FITC).
- [x] The axis scales are clearly visible. Include numbers along axes only for bottom left plot of group (a 'group' is an analysis of identical markers).
- [x] All plots are contour plots with outliers or pseudocolor plots.
- [x] A numerical value for number of cells or percentage (with statistics) is provided.

Methodology

Sample preparation

For isolation of CD4+ T cells and B cells from blood: Approximately 1 x 10^6 cells from the purification preparation or 1 x 10^6 cells from whole blood (following two RBC lysis steps, as described above) were incubated with Fc receptor block (1:200, BD Biosciences) for 15 minutes at room temperature. Then, immunofluorescent antibodies were added to the cells and cells incubated for 30 minutes at room temperature. B cell preparations were stained with CD19-PerCP/Cy5.5 (3:100, BioLegend); CD4+ T cell preparations were stained with CD3-FITC (1:10, BD Biosciences), CD4-V450 (1:10, BD Biosciences) and CD8-PerCP/Cy5.5 (1:10, BD Biosciences). Stained cells were washed twice in FACS (fluorescence-activated cell sorting) buffer (1mM EDTA, 25mM HEPES, 1% BSA (w/v) in PBS).

For CD4+ T cell and B cell stimulation analyses: Cells were seeded at approximately 1 x 10^6 cells/mL in RPMI-G on a TC-treated 96-well plate (180 µL/well). CD4+ T cells were stimulated with immobilized anti-human CD3ε (0.5 µg, BD Biosciences) and PMA (10 ng/mL, Sigma), as previously described (41,42). B cells were stimulated with human IL-4 (20 ng/mL, Sigma) and anti-human CD40 monoclonal antibody (5 µg/mL, Enzo, clone mAb 89), as previously described (43). Immediately following stimulation, cells were treated with either PBS or R-P4 (20 µM) in technical triplicate. As controls, a group of CD4+ T cells and B cells received no stimulus and no treatment (designated as "PBS (unstimulated)"). After incubating for 48 hours at 37 °C, cells were treated with human Fc block (1:200, BD Biosciences), stained with anti-CD69-PE/Cy7 (10 µL/test, BD Biosciences, clone FN50), washed, and resuspended in DAPI (0.5 µM, Thermo).

	For PI/Annexin V studies: PI uptake and AV staining were measured concurrently, as previously described (23.) Briefly, T cells and B cells were isolated as described above and were washed in PBS and resuspended in AV binding buffer (10mM HEPES, 140 mM NaCl, 2.5 mM CaCl2 in PBS) at a concentration of approximately 3.33 x10^6 cells/mL. Cells were then incubated with AV-Alexa Fluor 488 (1:20, Invitrogen) and PI (12.5 µg/mL, Life Technologies) for 15 minutes at room temperature, protected from light. Cells were then diluted by a factor of 5 in AV binding buffer into FACS tubes (BD Biosciences) and treated with hyper-hemolytic GBS or non-hemolytic GBS at an MOI of 10 or granadaene (0.5 µM) or an equivalent volume of GBSΔcylE extract.
Instrument	LSR II (BD Biosciences)
Software	Data collected using BD FACSDiva software and analyzed using FlowJo v. 10.1
Cell population abundance	Isolation of CD4+ T cells and B cells: After gating on size (SSC-A x FSC-A) and single cells (FSC-H x FSC-A), CD4+ T cell preparations were >90% CD3+/CD4+/CD8-. After gating on size (SSC-A x FSC-A) and single cells (FSC-H x FSC-A), B cell preparations were >90% CD19+.<br><br>CD4+ T cell and B cell simulation analyses: See Supplementary Fig. 3 b, c for abundance of cells that were DAPI- (after gating on size (SSC-A x FSC-A) and single cells (FSC-H x FSC-A). See Fig. 4 b, c for abundance of DAPI- cells that were CD69+ (after gating on size and DAPI).<br><br>PI/Annexin V studies: All events were included in PI/Annexin V staining over time. See manuscript and Fig. 3 b and e and Supplementary Fig. 2 for cell population abundance in PI/Annexin V gates.
Gating strategy	Isolation of CD4+ T cells and B cells: Cells were gated on size (SSC-A x FSC-A) and single cells (FSC-H x FSC-A). Fluorochrom gates were determined using unstained cells or fluorescence minus one controls following compensation with single-stained controls. CD4+ T cells were identified as CD3+/CD4+.<br><br>CD4+ T cell and B cell simulation analyses: See Supplementary Fig. 3 a for gating strategy. Fluorochrom gates were determined using unstained cells or fluorescence minus one controls following compensation with single-stained controls.<br><br>PI/Annexin V studies: PI/Annexin V gates were determined using unstained cells following compensation with single-stained controls.

☒ Tick this box to confirm that a figure exemplifying the gating strategy is provided in the Supplementary Information.
